# Supplementary material for: Myo-inositol nutritional supplement for prevention of gestational diabetes (EMmY): a randomised, placebo-controlled, double-blind pilot trial with nested qualitative study
Source: BMJ Open. 2022 Mar 10;12(3):e050110. doi: 10.1136/bmjopen-2021-050110 (PMC8919454; doi:10.1136/bmjopen-2021-050110)
Supplement: Supplementary data [file bmjopen-2021-050110supp001.pdf]

Appendix 1 Clinical maternal and perinatal outcomes assessed in the EMmY study.

**Clinical outcomes**	
**Maternal**	**Fetal and neonatal**
Gestational diabetes	Hypocalcaemia
Pre-eclampsia	Hypoglycaemia
Postpartum haemorrhage	Birth weight
Gestational age at delivery	Macrosomia (birth weight >4.5Kg),
Preterm delivery (< 34 and < 37 weeks)	small for gestational age (<10th centile)
Mode of delivery	Large for gestational age (>90th Centile)
Perineal trauma	Respiratory distress syndrome
Admission to the ITU	Shoulder dystocia
Admission to HDU	Apgar score at 10 minutes
Maternal infection	Birth trauma
Maternal death	Hyperbilirubinaemia
	Septicaemia
	Admission to NICU
	Still birth
	Neonatal death.

NB: Gestational diabetes is diagnosed based on the National Institute for Care and Excellence (NICE) and International Association of Diabetes and Pregnancy Study Groups (IADPSG) criteria.
ITU - intensive care unit, HDU - High dependency unit, NICU - neonatal intensive care unit.

Appendix 2 Criteria for progression to a full-scale trial

**Feasibility objectives and related data to be collected**	**GREEN zone**	**AMBER zone**	**RED zone**
i) Consent rates of eligible women	At least 25% of eligible women agreeing to participate.	Rate between 11 and 24% women agreeing to participate	Rate <10% of eligible women agreeing to participate
ii) Attrition rate after randomisation by 28 weeks, and by delivery (withdrawal or loss to follow up)	No more than 20% of randomised women lost or withdrawn	21-50% of randomised women lost or withdrawn	More than 50% of randomised women lost or withdrawn
iii) Rate of women who take more than 75% of sachets by 28 weeks and by delivery (of those who remain on the study)	Adherence to allocated treatment in >80% of women remaining on the study.	Adherence to allocated treatment in between 51-79% of women remaining on the study.	Adherence to allocated treatment in <50% of women
iv) Collection of data on clinical outcomes.	Complete data available of >80% of study sample.	Missing data between 21-50% of study sample.	Data missing of >50% of study sample.

Appendix 3 Characteristics of the 15 women interviewed

Ethnicity	Educational level	History of GDM (Y/N)	Adherence level (%)
Middle Eastern	Higher	N	50-75
South Asian	Higher	Y	>75
Middle Eastern	Higher	N	>75
White European	Higher	Y	>75
White European	Secondary	N	<50
South Asian	Higher	N	>75
South Asian	Higher	Y	>75
Black African/ Caribbean	Higher	N	<50
South Asian	Higher	N	>75
South Asian	Secondary	N	<50
White European	Higher	N	<50
White European	Secondary	N	>75
White European	Higher	N	>75
White European	Higher	N	<50
Middle Eastern	Higher	Y	>75

Appendix 4 Proportion of screened, recruited and randomised women in each participating site

Sites	Women screened (N)	Eligible of those screened n(%)	Recruited of those eligible n(%)	Randomised of those recruited n(%)
MRI	85	78 (91.8%)	46 (59%)	46 (100%)
NUH	144	115 (79.9%)	22 (19.1%)	22 (100%)
RLH	320	192 (60%)	53 (27.6%)	49 (92.5%)
STH	177	145 (81.9%)	41 (28.3%)	38 (92.7%)
WXH	600	243 (40.5%)	43 (17.7%)	43 (100%)
Overall	1326	773 (58.3%)	205	198

NB: MRI- Manchester Royal Infirmary (St Mary's Hospital) in Manchester, NUH- Newham Hospital, London, RLH- Royal London Hospital, London, STH- St George's Hospital, London, WXH- Whipps Cross Hospital, London.

Appendix 5 Impact of adherence at 28 weeks on glycaemia related outcomes

**Glycaemia related outcomes (unit) (intervention; control)***			
**≥ 50%**			
OGTT fasting (mmol/l) (40;40)	4.57 (0.6)	4.57 (0.5)	0.00 (0.2;0.2)
OGTT 2 hours (mmol/l) (39;40)	6.15 (2.2)	6.27 (1.5)	-0.11 (-0.9;0.7)
C-peptide (µg/L) (53;63)	1608.65 (723.2)	1907.71 (868.4)	-288.79 (-644.1;59.7)
Leptin (µg/L) (53;63)	42.81 (23.4)	39.31 (15.0)	4.09 (-4.8;12.7)
Insulin (mlU/L) (37;35)	11.23 (6.74)	14.81 (9.67)	**-3.34 (-6.61; -0.12)**
Adiponectin (mg/L) (53;63)	6.5 (3.6)	6.16 (3.6)	0.21 (-1.3;2.0)
Urinary inositol (mg/L) (53;65)	261 (164.6)	191.02 (117.7)	69.52 (3.2;136.8)
HOMA-IR (50;62)	2.25 (1.4)	3.06 (2.3)	**-0.8 (-1.6;-0.0)**
**≥ 75%**			
OGTT fasting (mmol/l) (34;32)	4.58 (0.6)	4.58 (0.5)	0.00 (0.3;0.3)
OGTT 2 hours (mmol/l) (33;32)	6.12 (2.35)	6.22 (1.53)	-0.1 (-1;0.9)
C-peptide (µg/L) (31;28)	1517.42 (573.9)	1937.86 (932.9)	**-425.4 (-808.8; -45.6)**
Leptin (µg/L) (31;28)	41.92 (24.4)	37.51 (11.9)	4.8 (-5.6;14.4)
Insulin (mlU/L) (31;28)	10.75 (5.78)	15.15 (10.4)	-4.57 (-8.2;0.99)
Adiponectin (mg/L) (31;28)	6.57 (3.6)	5.7 (3.4)	0.87 (-0.9;2.7)
Urinary inositol (mg/L) (32;27)	249.41 (152.7)	189.17 (110.3)	57.2 (-8.7;129.1)
HOMA-IR (28;27)	2.12 (1.1)	3.12 (2.5)	**-1.07 (-2; -0.2)**

NB: *GDM- Gestational Diabetes Mellitus, NICE- National Institute for Health and Care Excellence, IADPSG- International Association of Diabetes and Pregnancy Study Groups, GA- Gestational Age*
**Number of women in the intervention and control group respectively.*

Appendix 6 Costs and QALYS in both intervention and control groups

**Resource use costs**	**Intervention (*myo*-inositol) (£)**	**Control (placebo) (£)**
Supplements	2527	492
Laboratory tests	518	574
- Average costs (n)	5.51 (94)	6.17 (93)
Clinic visits	31,815	39,995
- Average costs per woman (n. of visits)	321.4 (390)	404 (389)
Adverse events	n/a	n/a
Delivery	309,268	303,753
- Average costs per woman (n)	3,514 (88)	3,472 (89)
**Total costs**	**312,121**	**344,814**
QALYs		
- Average QALY per woman (n)	0.51 (56)	0.53 (62)
**Total QALYs**	**28.4**	**32.6**

NB: QALYs – Quality Adjusted Life Years

Appendix 7 Completeness of clinical outcome data

**Proportion of data missing**			
**Clinical outcomes**	**Intervention (Myo-inositol) N = 99 n/N (%)**	**Control (Placebo) n = 99 n/N (%)**	**Total n = 198 n/N (%)**
GDM (NICE)- 28 weeks	14 (14.1%)	17 (17.2%)	31 (15.7%)
GDM (IADPSG)- 28 weeks	15 (15.2%)	17 (17.2%)	32 (16.2%)
GDM (all definitions)- by delivery	8 (8.1%)	7 (7.1%)	15 (7.6%)

***NB**: some GDM diagnosis were made between 28 weeks gestation and delivery, so would be missing at 28 weeks but not at delivery*
