# Supplementary material for: Myo-inositol nutritional supplement for prevention of gestational diabetes (EMmY): a randomised, placebo-controlled, double-blind pilot trial with nested qualitative study
Source: BMJ Open. 2022 Mar 10;12(3):e050110. doi: 10.1136/bmjopen-2021-050110 (PMC8919454; doi:10.1136/bmjopen-2021-050110)
Supplement: Author's manuscript [file bmjopen-2021-050110.draft_revisions.pdf]

BMJ Open

BMJ Open is committed to open peer review. As part of this commitment we make the peer review history of every article we publish publicly available.

When an article is published we post the peer reviewers' comments and the authors' responses online. We also post the versions of the paper that were used during peer review. These are the versions that the peer review comments apply to.

The versions of the paper that follow are the versions that were submitted during the peer review process. They are not the versions of record or the final published versions. They should not be cited or distributed as the published version of this manuscript.

BMJ Open is an open access journal and the full, final, typeset and author-corrected version of record of the manuscript is available on our site with no access controls, subscription charges or pay-per-view fees (http://bmjopen.bmj.com).

If you have any questions on BMJ Open's open peer review process please email info.bmjopen@bmj.com

BMJ Open

Myo-inositol nutritional supplement for prevention of gestational diabetes (EMmY): a randomised, placebo-controlled, double-blind pilot trial with nested qualitative study

Journal:	*BMJ Open*
Manuscript ID	bmjopen-2021-050110
Article Type:	Original research
Date Submitted by the Author:	09-Feb-2021

Complete List of Authors:	Amaefule, Chiamaka; Queen Mary University of London Barts and The London School of Medicine and Dentistry, BARC (Barts Research Centre for Women's Health), Institute of Population Health Sciences<br>Drymoussi, Zoe; Queen Mary University of London Barts and The London School of Medicine and Dentistry, (BARC) Barts Research Centre for Women's Health, Institute for Population Health Sciences<br>Gonzalez Carreras, Francisco; Queen Mary University of London Barts and The London School of Medicine and Dentistry, BARC (Barts Research Centre for Women's Health), Institute of Population Health Sciences<br>Pardo Llorente , Maria del Carmen; Complutense University of Madrid, Department of Statistics and Operational Research<br>Lanz, Doris; Queen Mary University of London Barts and The London School of Medicine and Dentistry, BARC (Barts Research Centre for Women's Health), Institute of Population Health Sciences<br>Dodds, Julie; Queen Mary University of London Barts and The London School of Medicine and Dentistry, BARC (Barts Research Centre for Women's Health), Institute of Population Health Sciences<br>Sweeney, Lorna; University of East London, Institute for Health and Human Development<br>Pizzo, Elena; University College London, NIHR ARC (Applied Health Collaborations) for North Thames London, Department of Applied Health Research<br>Thomas, Amy; Queen Mary University of London Barts and The London School of Medicine and Dentistry, BARC (Barts Research Centre for Women's Health), Institute of Population Health Sciences; The Royal London Hospital, Women's and Children's Health, Barts Health NHS Trust<br>Heighway, James; Queen Mary University of London Barts and The London School of Medicine and Dentistry, BARC (Barts Research Centre for Women's Health), BARC (Barts Research Centre for Women's Health), Institute of Population Health Sciences<br>Daru, Jahnavi; Queen Mary University of London Barts and The London School of Medicine and Dentistry, (BARC) Barts Research Centre for Women's Health, Institute for Population Health Sciences<br>Sobhy, Soha ; Queen Mary University of London Barts and The London School of Medicine and Dentistry, (BARC) Barts Research Centre for Women's Health, Institute for Population Health Sciences; The Royal London Hospital, Women's and Children's Health, Barts Health NHS Trust<br>Poston, Lucilla; King's College London, Department of Women and

Children's Health, Faculty of Life Sciences and Medicine
Khalil, Asma; St George's Hospital, Department of Obstetrics and Gynaecology; St George's University of London, Vascular Biology Research Centre, Molecular and Clinical Sciences Research Institute
Myers, Jenny; The University of Manchester, Maternal and Fetal Health Research Centre, Manchester Academic Health Science Centre
Harden, Angela; University of East London, Institute for Health and Human Development
Hitman, Graham; Queen Mary University of London Barts and The London School of Medicine and Dentistry, Centre for Genomics and Child Health, Blizard Institute
Khan , Khalid; Universidad de Granada, Department of Preventive Medicine and Public Health
Zamora, Javier; IRYCIS, Clinical Biostatistics Unit and CIBER Epidemiology and Public Health; University of Birmingham, WHO Collaborating Centre for Global Women's Health, Institute of Metabolism and Systems Research
Pérez, Teresa ; Queen Mary University of London Barts and The London School of Medicine and Dentistry, BARC (Barts Research Centre for Women's Health), Institute of Population Health Sciences; Complutense University of Madrid, Department of Statistics and Data Science
Huda, Mohammed SB; The Royal London Hospital Diabetes Diabetes and Metabolism, Barts Health NHS Trust
Thangaratinam, Shakila; University of Birmingham, WHO Collaborating Centre for Global Women's Health, Institute of Metabolism and Systems Research; Birmingham Women's and Children's NHS Foundation Trust

Keywords: Diabetes in pregnancy < DIABETES & ENDOCRINOLOGY, HEALTH ECONOMICS, Maternal medicine < OBSTETRICS, QUALITATIVE RESEARCH, PUBLIC HEALTH

SCHOLARONE™
Manuscripts

I, the Submitting Author has the right to grant and does grant on behalf of all authors of the Work (as defined in the below author licence), an exclusive licence and/or a non-exclusive licence for contributions from authors who are: i) UK Crown employees; ii) where BMJ has agreed a CC-BY licence shall apply, and/or iii) in accordance with the terms applicable for US Federal Government officers or employees acting as part of their official duties; on a worldwide, perpetual, irrevocable, royalty-free basis to BMJ Publishing Group Ltd ("BMJ") its licensees and where the relevant Journal is co-owned by BMJ to the co-owners of the Journal, to publish the Work in this journal and any other BMJ products and to exploit all rights, as set out in our licence.

The Submitting Author accepts and understands that any supply made under these terms is made by BMJ to the Submitting Author unless you are acting as an employee on behalf of your employer or a postgraduate student of an affiliated institution which is paying any applicable article publishing charge ("APC") for Open Access articles. Where the Submitting Author wishes to make the Work available on an Open Access basis (and intends to pay the relevant APC), the terms of reuse of such Open Access shall be governed by a Creative Commons licence – details of these licences and which Creative Commons licence will apply to this Work are set out in our licence referred to above.

Other than as permitted in any relevant BMJ Author's Self Archiving Policies, I confirm this Work has not been accepted for publication elsewhere, is not being considered for publication elsewhere and does not duplicate material already published. I confirm all authors consent to publication of this Work and authorise the granting of this licence.

For peer review only

Myo-inositol nutritional supplement for prevention of gestational diabetes (EMmY): a randomised, placebo-controlled, double-blind pilot trial with nested qualitative study

Chiamaka Esther Amaefule[1] Email: c.e.amaefule@qmul.ac.uk; Zoe Drymoussi[1] Email: z.drymoussi@qmul.ac.uk; Francisco Jose Gonzalez Carreras [1] Email: f.j.gonzalezcarreras@qmul.ac.uk; Maria del Carmen Pardo [1,2] Email: mcapardo@mat.ucm.es; Doris Lanz[1] Email: d.lanz@qmul.ac.uk; Julie Dodds[1] Email: jpdodds247@gmail.com; Lorna Sweeney[3] Email: lorna.christine@gmail.com; Elena Pizzo[4] Email: e.pizzo@ucl.ac.uk; Amy Thomas[1,5] Email: amy.thomas28@nhs.net; James Heighway[1] Email: j.heighway@qmul.ac.uk; Jahnavi Daru[1] Email: j.daru@qmul.ac.uk; Soha Sobhy[1,5] Email: soha.sobhy@qmul.ac.uk; Lucilla Poston[6] Email: lucilla.poston@kcl.ac.uk; Asma Khalil[7,8] Email: akhalil@sgul.ac.uk; Jenny Myers[9] Email: Jenny.Myers@manchester.ac.uk; Angela Harden[3] Email: a.harden@uel.ac.uk; Graham A Hitman[10] Email: g.a.hitman@qmul.ac.uk; Khalid Saeed Khan[11] Email: profkkhan@gmail.com; Javier Zamora [12, 13] Email: javier.zamora@hrc.es; Teresa Pérez [1,14] Email: teperez@estad.ucm.es; Mohammed SB Huda[15] Email: bobby.huda1@nhs.net; Shakila Thangaratinam [13,16]* Email: s.thangaratinam.1@bham.ac.uk

* Corresponding author

[1] BARC (Barts Research Centre for Women's Health), Institute of Population Health Sciences, Barts and the London School of Medicine and Dentistry, Queen Mary University of London, London, UK

[2] Department of Statistics and Operational Research, Complutense University of Madrid, Madrid Spain.

[3]Centre for Effectiveness Services, Harcourt Street, Dublin 2, Ireland

[4] NIHR ARC (Applied Research Collaborations) for North Thames London, Department of Applied Health Research, University College London, London, UK.

For peer review only - http://bmjopen.bmj.com/site/about/guidelines.xhtml

[5]Women's and Children's Health, Royal London Hospital, Barts Health NHS Trust, London UK.

[6] Department of Women and Children's Health, Faculty of Life Sciences and Medicine, King's College London, London UK.

[7] Department of Obstetrics and Gynaecology, St George's Hospital, London UK.

[8] Vascular Biology Research Centre, Molecular and Clinical Sciences Research Institute, St George's University of London, Cranmer Terrace, London UK.

[9] Maternal and Fetal Health Research Centre, University of Manchester, Manchester Academic Health Science Centre, UK.

[10] Centre for Genomics and Child Health, Blizard Institute, Barts and the London School of Medicine and Dentistry, Queen Mary University of London, London, UK.

[11] Department of Preventive Medicine and Public Health, Universidad de Granada, Granada, Spain.

[12] Clinical Biostatistics Unit (IRYCIS) and CIBER Epidemiology and Public Health, Madrid, Spain.

[13] WHO Collaborating Centre for Global Women's Health, Institute of Metabolism and Systems Research, University of Birmingham, Birmingham, UK.

[14] Department of Statistics and Data Science, Complutense University of Madrid, Madrid, Spain.

[15] Department of Diabetes & Metabolism, Barts Health NHS Trust, Royal London Hospital, London, UK.

[16] Birmingham Women's and Children's NHS Foundation Trust, Birmingham, UK.

WORD COUNT: 4076

For peer review only

For peer review only - http://bmjopen.bmj.com/site/about/guidelines.xhtml

ABSTRACT

Aim: To determine the feasibility and acceptability of conducting a randomised trial on the effects of *myo*-inositol in preventing gestational diabetes in high-risk pregnant women.

Methods: A multicentre, double-blind, placebo-controlled, pilot randomised trial with nested qualitative evaluation. Pregnant women were recruited from five inner city UK National Health Service hospitals at 12^{+0} and 15^{+6} weeks gestation with risk factors for gestational diabetes. Women took 2g of *myo*-inositol or placebo, both included 200µg folic acid, twice daily until delivery.

Primary outcome measures: Rates of recruitment, randomisation, adherence and follow-up.

Secondary outcome measures: Glycaemic indices (including homeostatic model assessment-insulin resistance HOMA-IR), gestational diabetes (diagnosed using oral glucose tolerance test at 28 weeks and by delivery), maternal, perinatal outcomes, acceptability of intervention, and costs.

Results: Of the 1326 women screened, 58% (773/1326) were potentially eligible, and 27% (205/773) were recruited. We randomised 97% (198/205) of all recruited women (99 each in intervention and placebo arms), and ascertained outcomes in 90% of women (178/198) by delivery. The mean adherence was 52% (SD 44) at 28weeks' and 34% (SD 41) at 36 weeks' gestation. HOMA-IR and serum insulin levels were lower in the myo-inositol vs placebo arm (Mean Difference -0.6, 95% CI -1.2 to 0.0 and -2.69, 95% CI -5.26 to -0.18 respectively). The study procedures were acceptable to women and healthcare professionals. Women who perceived themselves at high risk of gestational diabetes were more likely to participate and adhere to the intervention. The powder form of *myo*-inositol and placebo, along with nausea in pregnancy were key barriers to adherence.

Conclusions: A future trial on *myo*-inositol vs placebo to prevent gestational diabetes is feasible. The intervention will need to be delivered in a non-powder form to improve adherence. There is a signal for efficacy in reducing insulin resistance in pregnancy with *myo*-inositol.

Trial registration: ISRCTN48872100

For peer review only - http://bmjopen.bmj.com/site/about/guidelines.xhtml

Funding: Funded by Barts Charity, grant number MGU0373

Key words: diabetes in pregnancy, maternal medicine, qualitative research, public health, health economics

Abbreviations: CONSORT (Consolidated Standards of Reporting Trials); EQ-5D-5L (European Quality of life 5-Dimensions 5-Level scale); gestational diabetes; IADPSG (International Association of Diabetes and Pregnancy Study Groups); LGA (Large for Gestational Age); LTFU (Lost To Follow-Up); NHS (National Health Service); NICE (National Institute for Health and Care Excellence); NIHR (National Institute for Health Research); QALY (Quality-Adjusted Life-Year)

STRENGTHS AND LIMITATIONS

- The EMmY study is the first multi-centre trial involving ethnically diverse high-risk women from inner city NHS hospitals in the UK, looking at *myo*-inositol vs placebo during pregnancy.
- It is feasible to complete a full scale RCT on the effect of *myo*-inositol vs placebo in preventing gestational diabetes in high-risk women.
- Women do not prefer the powder of the intervention and may find a tablet form more acceptable.
- *Myo*-inositol use in pregnancy may reduce insulin resistance.
- If *myo*-inositol is shown to prevent gestational diabetes, this simple intervention could be easily implemented in clinical practice to improve maternal and perinatal outcomes.

For peer review only

INTRODUCTION

Primary prevention of gestational diabetes, a condition with high glucose levels first identified in pregnancy, is key to minimising maternal and perinatal complications associated with the condition.[1] Prevention of gestational diabetes can also reduce the risk of type 2 diabetes in mothers and their children in the long term. [2,3] Simple, easy to administer, and acceptable interventions are needed to prevent gestational diabetes.

Myo-inositol, a nutritional supplement that is present in fruits and fibre rich food and available as over-the-counter nutritional supplement, has the potential to prevent gestational diabetes through its insulin sensitising action. Few small trials have shown a promising role for *myo*-inositol with reductions in rates of gestational diabetes by up to 60%.[4, 5] But, in addition to small sample sizes (< 300 women), these trials were from a single country (Italy) and involved only Caucasian women.[5] The generalisability of these findings to high risk multi-ethnic populations who are most at risk of gestational diabetes is not known. A Cochrane review has called for further large trials on the effects of *myo*-inositol on gestational diabetes and complications in multi-ethnic populations. We also need to determine the costs and health service use associated with the intervention.[6]

Prior to undertaking a full-scale definitive trial on the effect of *myo*-inositol in preventing gestational diabetes, which requires substantial resources, we need to assess the feasibility of conducting such a trial beforehand. We undertook a pilot randomised trial comparing *myo*-inositol vs placebo, with a nested qualitative study, to assess the feasibility of the trial design, explore the acceptability and contextual issues around intervention delivery and trial procedures, and the potential effects on glycaemic, maternal, perinatal, cost, and quality of life outcomes.

For peer review only - http://bmjopen.bmj.com/site/about/guidelines.xhtml

METHODS

The EMmY trial was a multicentre, randomised, placebo-controlled, double-blind pilot trial with nested qualitative study and economic evaluation conducted with a prospective protocol and reported using CONSORT (Consolidated Standards of Reporting Trials) guidelines.[7] The full details of the study protocol are published elsewhere.[8]

Participants and setting

We screened pregnant women attending five inner city maternity units in London and Manchester. Women were eligible for recruitment if they were at least 16 years of age with a viable singleton pregnancy between 12^{+0} and 15^{+6} weeks gestation, were able to provide written informed consent in English, and were considered to be at high risk of gestational diabetes as per the nice (national institute for health and Care Excellence) criteria.[9] We excluded women with type 2 diabetes, and those taking metformin or corticosteroids. Recruited women with previous history of gestational diabetes underwent additional testing with OGTT (oral glucose tolerance test) or HbA1c, and were randomised after ruling out undiagnosed type 2 diabetes (OGTT fasting ≥5.6 mmol/l, 2hour glucose ≥7.8 mol/l or HbA1c ≥48 mmol/mol).

Intervention and control group allocation

The intervention group were provided with *myo*-inositol 2g supplement with 200μg folic acid, to be taken twice a day in a powder form mixed in water, approximately 1 hour before or after a meal. The placebo was an identical looking and tasting powder of Xylitol filler with 200μg folic acid to be taken twice a day.

Outcomes

The primary outcomes were the proportion of screened women who were eligible, the proportion of eligible women who were recruited and randomised, rates of adherence and follow-up. The

For peer review only

For peer review only - http://bmjopen.bmj.com/site/about/guidelines.xhtml

secondary outcomes were acceptability of the intervention and trial to women and health professionals; laboratory outcomes related to glycaemia such as fasting and 2-hour plasma glucose levels in 75g OGTT, HOMA-IR, serum insulin, adiponectin, leptin, maternal and cord blood c-peptide, and urinary inositol. Other secondary outcomes included diagnosis of gestational diabetes at 28 weeks' gestation by modified IADPSG (International Association of Diabetes and Pregnancy Study Groups) and NICE criteria, maternal and perinatal morbidity and mortality (Appendix 1). The economic outcomes included costs (NHS health and social care resources use) and quality of life. Other process outcomes included deviations from study protocol, completeness of data collection and the level of support required by the site team for trial delivery.

Sample size

We expected that 1500 women would be booked for antenatal care each month at the participating hospitals, and at least 300 of those would be eligible. Assuming 1000 eligible women were approached, we expect 25% (250/1000) to be consented. We expected that 20% (50) of these would be women with a previous history of gestational diabetes, with abnormal HbA1C and/ or OGTT and would be excluded. This would result in 200 women randomised.

Study conduct

We approached pregnant women at booking and screened them for eligibility. After obtaining written informed consent from eligible women, we collected baseline information on demographic and clinical characteristics, and women completed the validated European Quality of life 5-Dimensions 5-Level scale (EQ-5D-5L) questionnaire on quality of life measures. Women were randomised between 12^{+0} and 15^{+6} weeks' gestation using an online randomisation system (administered by epiGenesys, University of Sheffield) to either the *myo*-inositol or placebo treatment arm. We used a randomisation scheme based on permuted blocks of random

For peer review only

For peer review only - http://bmjopen.bmj.com/site/about/guidelines.xhtml

block size (sizes 4, 6 and 8) stratified by participating site. No adaptive or minimisation strategies were used. Participants, healthcare providers and researchers were blinded to the group allocation. We followed-up women in person or by phone at 20, 28- and 36-weeks' gestation and at delivery. All women were offered OGTT at 28 weeks' gestation to screen for gestational diabetes. women self-reported adherence to the intervention using a paper-based diary, or an optional mobile application. Where possible, we supplemented this information with a count of unused sachets. The trial was overseen monthly by a Trial Management Group and biannually by the Project Steering Committee (combining functions from a more traditional set up of a separate Data Monitoring Committee and Trial Steering Committee) reviewing trial progress and conduct. We prespecified the criteria for progression to a full-scale trial (Appendix 2).

Analysis

We summarised the feasibility outcomes using proportions with 95% CI (confidence interval) and other descriptive statistics. For continuous laboratory outcomes, we calculated the effect sizes (e.g. mean differences) with 95% CI. We reported the rates of dichotomous clinical outcomes and costs in the two arms of the study. The impact of a previous history of gestational diabetes and the use of the mobile app on adherence were conducted as a post-hoc exploratory analysis. All analyses were performed using R, version 3.6.1.

Qualitative study

We explored the experiences and perspectives of women and healthcare professionals on the intervention and trial procedures, using observations and interview methods. Recruitment appointments were observed to ascertain women's specific motivations or concerns regarding participating in the trial, as well as contextual factors that may optimise or discourage participation. We invited eligible women who declined trial participation to give their reasons verbally to the researcher or complete an open-ended questionnaire for reasons for declining

For peer review only - http://bmjopen.bmj.com/site/about/guidelines.xhtml

consent. We interviewed women and healthcare professionals involved in trial delivery to explore the barriers and facilitators of participation, adherence and retention in the study as well as trial delivery in interviews with health professionals. Interview data was transcribed and analysed using a thematic analytical approach.[10] Purposive sampling was used to ensure the women interviewed were from diverse age groups, ethnic backgrounds, clinical characteristics and levels of adherence (Appendix 3). We contacted women who withdrew from the trial, and midwives to discuss in depth the reasons for withdrawal.

Economic evaluation

A cost-utility analysis of *myo*-inositol supplementation versus placebo "within trial" period was undertaken. The cost utility measures were the incremental cost per unit of change per Quality-Adjusted Life-Year (QALY) gained. Cost was assessed from the perspective of the NHS and personal social services. The unit costs were retrieved from published sources, calculated in pound sterling based on 2018 costs. Resource use data was collected retrospectively. For each participant, their EQ-5D-5L health state was converted to a single summary index (utility value), constructing a utility profile based on the assumption of a straight-line relation between their utility values and each measurement point. For each participant, QALYs from baseline to 28 weeks were calculated as the area under the utility profile.

Patient and Public Involvement (PPI)

We sought input from Katie's Team, a women's health and childbirth PPI advisory group[12], who contributed to the study design and patient-facing documents for the pilot trial, and the interview schedule for the qualitative study.[8] Patients and members of the public were not involved in the recruitment to and conduct of the study. We plan to disseminate results from the EMmY study to participants and members of Katie's Team, who can then circulate further through their networks and social media platforms.

For peer review only

RESULTS

Over six months (Feb - Sep 2018), we screened 1326 women across all five participating sites, and randomised 198 women to either intervention or control arm (Figure 1). The majority of participants in both arms were of ethnic minority origin (73% intervention; 60% control), over a third were obese (37% intervention; 36% control), and around half (51% intervention; 40% control) were nulliparous. About 60% of all women had a first degree relative with type 2 diabetes, and one in ten (8% intervention; 11% control) had a history of previous gestational diabetes (Table 1). Urinary inositol levels were higher in the *myo*-inositol than the placebo arm (MD 70.76 mg/L, 95% CI 11.2 to 130.8) (Table 2).

PRIMARY OUTCOMES

Of the 773 eligible women, 27% (205/773) consented to be involved in the study. Of these, 98% (198/205) were randomised to the trial after ruling out type 2 diabetes (Figure 1). Figure 2 and Appendix 4 show the rate of recruitment and randomisation across all and individual participating sites respectively. The commonest reason for women declining consent was a lack of interest in research (48%, 271/568) (Figure 1). Outcomes were assessed in 75% (148/198) of women at 28 weeks, and in 90% (178/198) of women were followed-up at delivery. The rates of adherence to the intervention in the *myo*-inositol (mean 53%, SD 45) and placebo (mean 50%, SD 43) groups were similar at 28 weeks. A sensitivity analysis showed that 75% (12/16) of women with gestational diabetes in previous pregnancies took more than 75% of *myo*-inositol sachets compared to 41% of women without a history of this condition (60/145). Six of the eight App users also took more than 75% of *myo*-inositol sachets.

SECONDARY OUTCOMES

Glycaemic outcomes

For peer review only - http://bmjopen.bmj.com/site/about/guidelines.xhtml

We found significant reductions in HOMA-IR (MD -0.6; 95% CI -1.2 to 0.0) and levels of insulin (MD -2.69; 95% CI -5.26 to -0.18) in the intervention group vs control. There were no differences between the groups in other glycaemic estimates (Table 2). Sensitivity analyses of women with over 50% and over 75% adherence to the intervention showed that larger reductions in HOMA-IR and insulin levels (Appendix 5).

Clinical outcomes

The proportion of women diagnosed as gestational diabetes were 14.1% (14/99) and 13.1% (13/99) in the intervention and control arms respectively at 28 weeks by modified IADPSG criteria. The overall diagnosis of gestational diabetes by delivery were 24.2% (24/99) and 20.2% (20/99) using any criteria (Table 3). The rates of preterm birth in the *myo*-inositol and placebo groups were 6% (6/99) and 10.2% (10/99) respectively, and large-for-gestational age (LGA) were 6.1% (6/99) in intervention and 11.2% (11/99) in control arm. There were no reports of any maternal anaphylactic reaction or maternal death (Table 3).

Adverse outcomes

Vomiting was reported by 15.1% (15/99) of women in the intervention arm and by 9.1% (9/99) in the control arm. Other gastrointestinal symptoms were similar between groups: diarrhoea was reported by 5.1% (5/99) and 4% (4/99) in the intervention and placebo arms respectively, and constipation by 5.1% (10/198) in both groups.

Cost outcomes

Baseline EQ-5D-5L questionnaires were completed by all women randomised, and end of trial EQ-5D-5L questionnaires by 57% (56/99) in the intervention arm and 63% (62/99) in the control group. The total cost of trial (including the supplements, diagnostic tests, clinic visits and cost of birth/delivery) was very similar in both groups (£344,127 versus £344,814 in the intervention

For peer review only

and control group respectively) and on average around £3500 per woman. The QALYs were similar in both groups with 0.53 QALYs over 28 weeks in the control and 0.51 QALYs in the intervention group. With no major difference in costs and QALYs between groups, an incremental cost-effectiveness ratio (ICER) was not calculated and further sensitivity analysis was not explored. The costs of resources used by women in both groups as well as QALYs are reported in Appendix 6.

QUALITATIVE FINDINGS

We observed the recruitment of 28 participants across three sites. Nine women who did not want to participate in the study provided input on reasons for declining consent. Fifteen women consented to a qualitative research interview. Fourteen healthcare professionals were interviewed, including research midwives with responsibility for recruitment at each participating site, the academic trial coordinator, the research coordinator at participating sites and a clinical academic.

Recruitment and participation

Women responded positively in the interviews about their experience of the recruitment process. They felt they received adequate information about the study to make a decision about participating, and that their questions and concerns were addressed. From our observations and interviews with women and research midwives, perceived risk of developing gestational diabetes was identified as a key factor influencing the decision about whether or not to participate in the trial. Women were more motivated to join the study if they believed they were at risk of developing gestational diabetes in their pregnancy, either due to a previous experience with the condition in an earlier pregnancy, or due to their awareness of gestational diabetes risk factors such as family history of diabetes or weight gain in pregnancy. Women without a previous history of gestational diabetes, or a family history of diabetes and those who felt they already

For peer review only

For peer review only - http://bmjopen.bmj.com/site/about/guidelines.xhtml

engaged in healthy lifestyle practices did not typically view themselves to be at risk of developing gestational diabetes and were more likely to decline consent (even if the study had identified them as at-risk due to their ethnic background).

> *"... in my first pregnancy I suffered a lot. I wasn't able to have anything…even if I had an apple…I used to climb the stairs up and down,…to…bring the sugar low…it's been a nightmare for me…I don't want that to happen again…I'm just following it religiously, just to make sure…"* (Farihah, South Asian with previous gestational diabetes)

> *"I've never had a patient with previous diabetes who declined the study."* (Midwife A, Site 1)

The "natural" make-up of *myo*-inositol as a vegetarian food supplement, rather than a drug, appeared to encourage trial participation. Many women felt reassured that it was safe to take the supplement in pregnancy and research midwives said it was ideal for the predominantly Muslim target population of the study, who only eat Halal foods.

> *"... It was something natural…a natural ingredient, nothing too chemical or new or anything. So it didn't worry me".* (Amber, White European with higher education level).

Nevertheless, some women cited *"fear of supplements"* on the decliners' questionnaire as their reason for declining consent and some were concerned that participating in research involved testing the effect of new drugs, which could potentially harm the baby. Partners with these concerns sometimes discouraged participation even when the woman had indicated interest in being part of the research. High staff turnover rates in most participating sites affected study conduct. The midwives, research coordinator and trial coordinator suggested an increase in staff capacity or provision of support staff during more intense periods of study recruitment and follow-up. Research midwives highlighted that recruitment success was dependent on dissemination of trial information and engagement with the wider antenatal team.

For peer review only - http://bmjopen.bmj.com/site/about/guidelines.xhtml

For peer review only

Adherence and retention

Several of the women interviewed experienced prolonged nausea and vomiting in pregnancy and discontinued the supplements when they felt these symptoms were made worse by drinking the supplement (several mouthfuls of *"sweet"*-tasting, *"sandy"*-textured liquid). Research midwives also cited pregnancy sickness as a major reason for participant non-adherence and withdrawal. A tablet form of the supplement was suggested by women and midwives as a preferable option in future studies. The twice-daily intake of the supplement and the time restrictions on taking it one hour before or after a meal were also major barriers to long-term adherence, as women struggled to embed the regimen into their busy daily routines.

> *"…if it was in tablet form it would be easier for them to just swallow it, just like the Pregnacare... But because it's in liquid they have to take a mouthful and mouthful and mouthful... And the main reason for the withdrawal again is the fact that they were having very bad morning sickness".* (Midwife G, Site 4)

Notably, adherence was reduced for some women after a normal or abnormal OGTT at the 28 weeks' visit. Those with a normal result felt the supplements were no longer needed and those with an abnormal result felt the strict regimen they had been following had not been effective. This was less of an issue for women who felt most at risk of developing gestational diabetes.

We were not able to fully evaluate the use of the App, due to difficulties in the NHS Wi-Fi connectivity, which in many cases prevented the download of the app at the point of randomisation. The few women who downloaded the App felt the automated reminders were helpful with the regimen and critical to their adherence, while other participants without the app felt they would have adhered to the sachets more consistently if they had been supported with App reminders. They also felt they would have been more consistent in recording their adherence if they could have done so on their personal mobile phones via an App, rather than in

For peer review only - http://bmjopen.bmj.com/site/about/guidelines.xhtml

a diary, which was likened to administrative work. By contrast, some women who were not as comfortable with using technology felt the diary was easier and more straightforward.

> *"… I think an App or something would have been better… something that reminded me to take it… I just saw the booklet, it's just like an admin thing. That didn't help me. So an App might have done because I've got all the pregnancy Apps on my phone to remind me to take other things …* (Amber, in full time employment)

DISCUSSION

Summary of key findings

Our multicentre pilot trial showed that it is feasible to recruit, randomise, adequately follow-up, and collect the relevant outcomes in a multi-ethnic inner-city pregnant population comprising of high-risk women, to evaluate the effects of *myo*-inositol on gestational diabetes. The trial procedures are acceptable to women and healthcare professionals. The powder form of the intervention was a barrier to adherence and women requested alternative forms such as a tablet. We observed a positive signal for efficacy in the *myo*-inositol arm than placebo for insulin resistance.

Strengths and limitations

Our pilot randomised trial was based on a clear prospective protocol,[8] and we reported the findings using recommended guidelines.[7] We showed that it is feasible to recruit the required numbers of participants within planned time frames, and to cost, across multiple maternity units. More than half of our participants were from black and ethnic minority backgrounds, who are at most risk of developing gestational diabetes. We identified the barriers and facilitators to recruitment as reported by participating mothers and healthcare professionals. The trial procedures were robustly carried out, with good accuracy in data collection (Appendix 7). The outcomes were ascertained in over 80% of women in the control and intervention arms. The

For peer review only - http://bmjopen.bmj.com/site/about/guidelines.xhtml

qualitative study identified the main factors to sub-optimal adherence, which will need to be addressed in a full-scale trial. We showed that other outcomes such as clinical and cost-related outcomes can be collected to inform the main scale trial.

Some of the limitations to the study included varying levels of recruitment rates across sites. Something to consider when planning the full-scale trial will be to increase staffing, to ensure that sites are fully supported during intense periods of participant recruitment and follow-up. There were more women of black and ethnic minority backgrounds in the intervention than control arms, who are more likely to have dysglycaemia in pregnancy. In a future full-scale trial, we plan to minimise by ethnicity to ensure both groups are fully balanced. Some of the qualitative insights suggested that adherence may have been improved if the App reminders had been made available for more women. Another limitation was not being able to assess the cord blood levels of the glycaemic markers in a larger proportion of participants.

Interpretation of findings and comparison with existing literature

Our consent rates were consistent with other pregnancy trials involving multi-ethnic groups, citing similar reasons why eligible participants declined consent.[13, 14] Previous trials on *myo*-inositol supplementation in pregnancy reported consent rates over 70% but had a predominantly Caucasian population.[15, 16] Other pregnancy trials reported similar attrition rates to the EMmY study, [15, 16] and made reference to phone-based reminders and diaries as crucial to adherence.[17, 18] The EMmY study was not powered to detect any statistical differences in clinical or laboratory outcomes, including for gestational diabetes. Hence, we refrained from reporting comparative estimates for dichotomous outcomes. We identified the expected rates of gestational diabetes in the control arm that will inform the sample size calculations for the full-scale trial. We also observed that women continued to be diagnosed with gestational diabetes beyond 28 weeks,

For peer review only - http://bmjopen.bmj.com/site/about/guidelines.xhtml

therefore a definitive trial would need to assess onset of gestational diabetes at any time in pregnancy along with critically important clinical outcomes as main outcomes.

Clinical and research implications

Overall, the pilot study achieved 'green' status in rates of recruitment, attrition, and data collection, and 'amber' in adherence to the intervention at 28 weeks, as markers of progression to full-scale trial. Our qualitative study has identified the main reason for reduced adherence to be dissatisfaction with the powder form of the intervention. The findings were further discussed with the independent Steering Committee and Katie's Team, the patient and public involvement support arm of the study, who recommended use of *myo*-inositol tablets instead of powder when progressing to a full-scale trial.

We explored the differences in the continuous laboratory outcomes for signals of efficacy, as these would inform the choice of main outcomes for the full-scale trial. Despite lower than anticipated overall adherence to the intervention, the higher levels of urinary inositol in the intervention group makes the comparison of outcomes valid. In randomised trials on *myo*-inositol in pregnancy to prevent or treat gestational diabetes, *myo*-inositol showed a significant reduction in levels of fasting and postprandial blood glucose, HOMA-IR and insulinaemia.[19-21] We found a reduction in insulin and HOMA-IR in the intervention arm vs control, indicating a potentially increased insulin sensitivity with *myo*-inositol. Insulin levels and HOMA-IR appeared to reduce further with improved adherence. Our findings are in line with previous observations that *myo*-inositol reduces blood glucose levels by acting as an insulin sensitising agent. [16, 21] We did not observe any differences in the fasting or 2-hour blood glucose levels, but improved adherence showed the potential to reduce 2-hour glucose levels in the intervention group. It is likely that with improved adherence, any potential differences between groups will become clearer in any future studies.

For peer review only - http://bmjopen.bmj.com/site/about/guidelines.xhtml

Although there were no differences in the levels of c-peptide between the two arms, women who adhered to the intervention appeared to have a reduction in c-peptide levels, indicative of a reduction in insulin resistance. Participants in our study continued taking *myo*-inositol even after the diagnosis of gestational diabetes. We observed that fewer women in the intervention arm had large for gestational age (LGA) babies than in the control arm. The small sample size of this pilot trial means no statistical significance can be attributed to these estimates.[22] A future large scale trial will be needed to evaluate if the improvement in insulin sensitivity with *myo*-inositol contributed to the observed reduction in LGA babies.

Conclusions

The EMmY pilot trial is the first UK based multi-centre trial involving ethnically diverse high-risk women from inner city NHS hospitals in a trial of *myo*-inositol vs placebo. We have reported on acceptability, cost and quality outcomes, as well as the potential effect of *myo*-inositol on gestational diabetes. There is an indication of efficacy of *myo*-inositol in lowering insulin resistance, with the potential for preventing gestational diabetes. In order to tackle the observed barriers to adherence, we plan to use a tablet form of *myo*-inositol in a large-scale definitive trial, with an internal pilot first demonstrating the intended levels of adherence. We now need a large-scale definitive trial adequately powered to ascertain the effect of *myo*-inositol in a tablet form on gestational diabetes and adverse maternal and perinatal outcomes.

For peer review only - http://bmjopen.bmj.com/site/about/guidelines.xhtml

References

1. Metzger BE, Lowe LP, Dyer AR, et al. Hyperglycemia and adverse pregnancy outcomes. *N Engl J Med*. May 2008;358(19):1991-2002. doi:10.1056/NEJMoa0707943

2. Bellamy L, Casas JP, Hingorani AD, Williams D. Type 2 diabetes mellitus after gestational diabetes: a systematic review and meta-analysis. *Lancet*. May 2009;373(9677):1773-9. doi:10.1016/S0140-6736(09)60731-5

3. Boney CM, Verma A, Tucker R, Vohr BR. Metabolic syndrome in childhood: association with birth weight, maternal obesity, and gestational diabetes mellitus. *Pediatrics*. Mar 2005;115(3):e290-6. doi:10.1542/peds.2004-1808

4. Rogozińska E, Chamillard M, Hitman GA, Khan KS, Thangaratinam S. Nutritional manipulation for the primary prevention of gestational diabetes mellitus: a meta-analysis of randomised studies. *PLoS One*. 2015;10(2):e0115526. doi:10.1371/journal.pone.0115526

5. Vitagliano A, Saccone G, Cosmi E, et al. Inositol for the prevention of gestational diabetes: a systematic review and meta-analysis of randomized controlled trials. *Arch Gynecol Obstet*. 01 2019;299(1):55-68. doi:10.1007/s00404-018-5005-0

6. Crawford TJ, Crowther CA, Alsweiler J, Brown J. Antenatal dietary supplementation with myo-inositol in women during pregnancy for preventing gestational diabetes. *Cochrane Database Syst Rev*. Dec 2015;(12):CD011507. doi:10.1002/14651858.CD011507.pub2

7. Eldridge SM, Chan CL, Campbell MJ, et al. CONSORT 2010 statement: extension to randomised pilot and feasibility trials. *Pilot Feasibility Stud*. 2016;2:64. doi:10.1186/s40814-016-0105-8

8. Amaefule CE, Drymoussi, Z., Dodds, J., Sweeney, L., Pizzo, E., Daru, J., Robson, J., Poston, L., Khalil, A., Myers, J. and Harden, A.,. Effectiveness and acceptability of myo-inositol nutritional supplement in the prevention of gestational diabetes (EMmY): a protocol for a randomised, placebo-controlled, double-blind pilot trial. *BMJ open*. 2018;8(9):p.e022831.

9. National Institute for Health and Care Excellence. Diabetes in pregnancy: management from pre-conception to the post-natal period. Accessed 26th November, 2019. https://www.nice.org.uk/guidance/ng3/chapter/1-Recommendations#gestational-diabetes-2

10. Braun, V. and Clarke, V. Using thematic analysis in psychology. Qualitative research in psychology. *Qualitative Research in Psychology*. 2006;*3* (2):pp.77-101.

11. Amaefule CE, Sweeney, L., Harden, A., Thangaratinam, S.,. Pilot trial on myo-inositol supplementation in pregnancy: A qualitative study with women and research staff on the feasibility and acceptability of the trial and intervention. *To be published at BMJ open*. 2020;

12. Moss N, Daru J, Lanz D, Thangaratinam S, Khan KS. Involving pregnant women, mothers and members of the public to improve the quality of women's health research. *BJOG*. Feb 2017;124(3):362-365. doi:10.1111/1471-0528.14419

13. Husain S, Allotey J, Drymoussi Z, et al. Effects of oral probiotic supplements on vaginal microbiota during pregnancy: a randomised, double-blind, placebo-controlled trial with microbiome analysis. *BJOG*. 01 2020;127(2):275-284. doi:10.1111/1471-0528.15675

14. Poston L, Bell R, Croker H, et al. Effect of a behavioural intervention in obese pregnant women (the UPBEAT study): a multicentre, randomised controlled trial. *Lancet Diabetes Endocrinol*. Oct 2015;3(10):767-77. doi:10.1016/S2213-8587(15)00227-2

15. D'Anna R, Scilipoti A, Giordano D, et al. myo-Inositol supplementation and onset of gestational diabetes mellitus in pregnant women with a family history of type 2 diabetes: a prospective, randomized, placebo-controlled study. *Diabetes Care*. Apr 2013;36(4):854-7. doi:10.2337/dc12-1371

16. D’Anna R, Di Benedetto A, Scilipoti A, et al. Myo-inositol Supplementation for Prevention of Gestational Diabetes in Obese Pregnant Women: A Randomized Controlled Trial. *Obstet Gynecol*. Aug 2015;126(2):310-5. doi:10.1097/AOG.0000000000000958

17. Laughland AaK, L.,. Should participants be left to their own devices? Comparing paper and smartphone diaries in psychological research. *Journal of applied research in memory and cognition*. 2018;7(4):pp.552-563.

For peer review only - http://bmjopen.bmj.com/site/about/guidelines.xhtml

18. Brennan M, Clarke M, Devane D, Dowling M. A qualitative study of the factors influencing recruitment to a pilot trial on the prevention of striae gravidarum. *BMC Pregnancy Childbirth*. Feb 2020;20(1):103. doi:10.1186/s12884-020-2781-x

19. Fraticelli F, Celentano C, Zecca IA, et al. Effect of inositol stereoisomers at different dosages in gestational diabetes: an open-label, parallel, randomized controlled trial. *Acta Diabetol*. Aug 2018;55(8):805-812. doi:10.1007/s00592-018-1157-4

20. Corrado F, D'Anna R, Di Vieste G, et al. The effect of myoinositol supplementation on insulin resistance in patients with gestational diabetes. *Diabet Med*. Aug 2011;28(8):972-5. doi:10.1111/j.1464-5491.2011.03284.x

21. Miñambres I, Cuixart G, Gonçalves A, Corcoy R. Effects of inositol on glucose homeostasis: Systematic review and meta-analysis of randomized controlled trials. *Clin Nutr*. 06 2019;38(3):1146-1152. doi:10.1016/j.clnu.2018.06.957

22. Arnold DM, Burns KE, Adhikari NK, et al. The design and interpretation of pilot trials in clinical research in critical care. *Crit Care Med*. Jan 2009;37(1 Suppl):S69-74. doi:10.1097/CCM.0b013e3181920e33

Authors' contributions

CEA- wrote the first and final version of the manuscript, collected and analysed the qualitative data.

ZD, DL, JDO, JH- coordinated the study, contributed to the methodology and the final version of the manuscript.

LS and AH- supervised the qualitative evaluation and has contributed to the final version of the manuscript.

EP- developed and conducted the economic evaluation. EP also contributed to the final version of the manuscript.

AT, JDa, SS, LP, AS, JM, GH, KSK and MSH- provided clinical input and contributed to the final version of the manuscript.

JZ, MDCP, TP- developed and lead the statistical evaluation and contributed to the final version of the manuscript.

FJGC- conducted the statistical analysis and contributed to the final version of the manuscript.

ST- designed and developed the trial, reviewed the manuscript and has contributed to the final version of the manuscript.

For peer review only - http://bmjopen.bmj.com/site/about/guidelines.xhtml

Acknowledgements

The authors acknowledge Pharmasure for the donation of *myo*-inositol and placebo tablets to the EMmY study and Montuno software for the discounted Dosecast app. The authors are grateful to the patient advisers and site midwives for their contributions towards the study.

The EMmY pilot trial was supported by the National Institute for Health Research (NIHR) Programme Grants for Applied Research. The views expressed are those of the authors and not necessarily those of the NHS, the NIHR or the Department of Health and Social Care.

KSK is a Distinguished Investigator funded by the Beatriz Galindo (Senior Modality) Program grant given to the University of Granada by the Ministry of Science, Innovation, and Universities of the Spanish Government.

Ethical approval and funding statement

The EMmY trial is sponsored by Queen Mary University of London and funded by Barts Charity, grant number MGU0373. Ethical approval was obtained from the London Queen Square Research Ethics Committee (17/LO/1741). The sponsor and the funder have no role in the study conduct, analysis and interpretation of the findings, and dissemination of the results. EP and AH are also supported by the NIHR Collaboration for Leadership in Applied Health Research at Barts Health NHS Foundation Trust (NIHR ARC North Thames). Professor Khalid S. Khan is Distinguished Investigator funded by the Beatriz Galindo (senior modality) grant to the University of Granada by the Spanish Ministry of Education.

Data Sharing Statement

Release of data will be subject to a data use agreement between the contractor and the third party requesting the data. The data use agreement must detail the agreed use and appropriate management of the research data to be shared and include a requirement for recognition of the contribution of the researchers who generated the data and the original funder.

Competing interests

None

Figure Legends, Tables and Appendices

Figure 1 CONSORT diagram for the EMmY study

Figure 2 Rate of recruitment and randomisation in all participating sites in the EMmY trial

Table 1 Baseline characteristics of EMmY participants randomised to *myo*-inositol vs placebo

Table 2 Glycaemic outcomes in the intervention and control arms of the EMmY trial

Table 3 Maternal and perinatal clinical outcomes in the EMmY study

Appendix 1 Clinical maternal and perinatal outcomes assessed within the EMmY study

Appendix 2 Criteria for progression to a full-scale trial

Appendix 3 Characteristics of the 15 women interviewed using pseudonyms

Appendix 4 Proportion of screened, recruited and randomised women in each participating site

Appendix 5 Costs and QALYS in both intervention and control groups

For peer review only

577 Appendix 6 Impact of adherence at 28 weeks on key clinical and laboratory outcomes

578 Appendix 7 Completeness of clinical outcome data

For peer review only

For peer review only - http://bmjopen.bmj.com/site/about/guidelines.xhtml

Figure 1 Flow chart of participants in the EMmY study

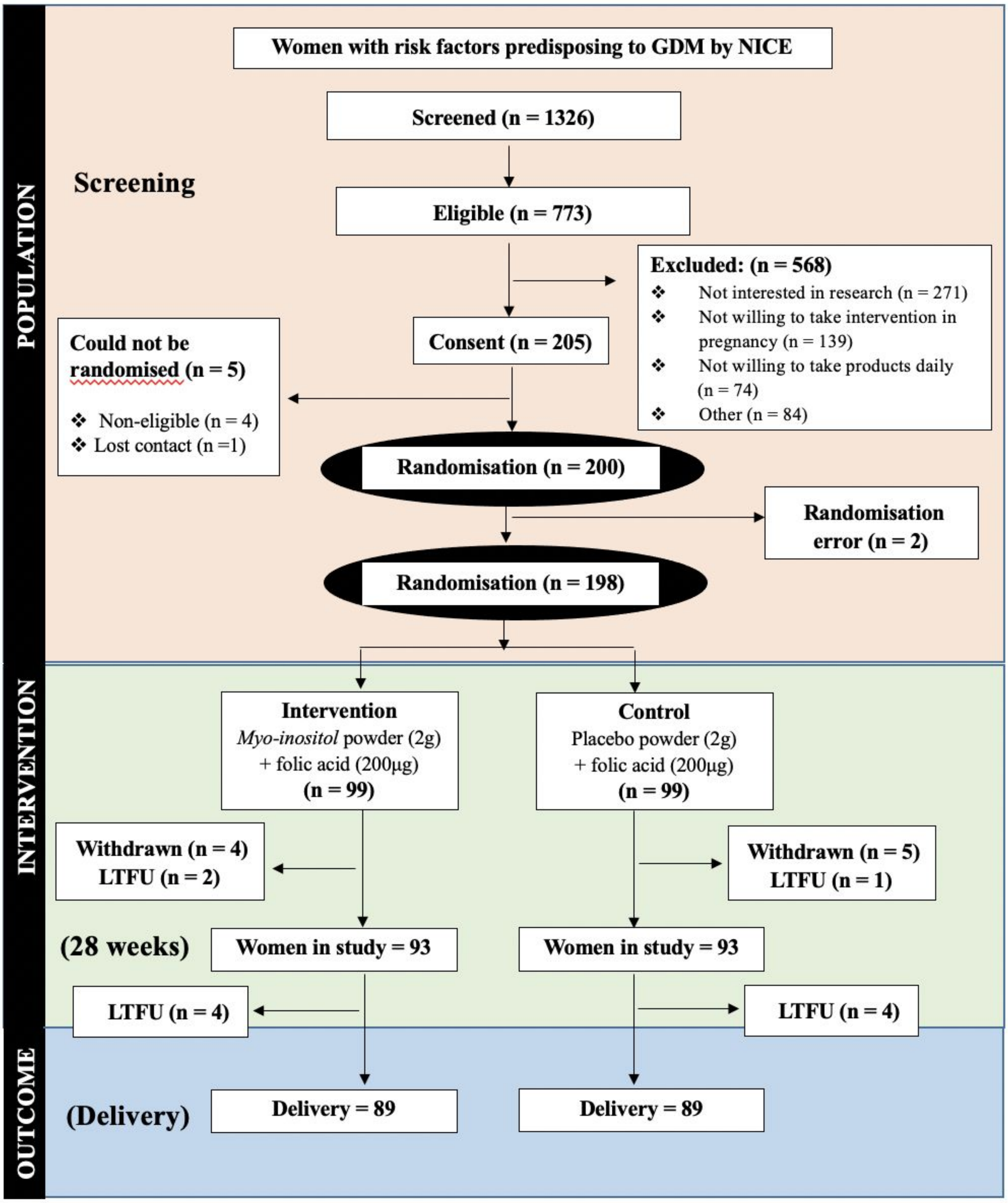

GDM- Gestational Diabetes Mellitus, NICE- National Institute for Health and Care Excellence, LTFU- Loss to Follow-Up

Figure 2 Rate of recruitment and randomisation in all participating sites in the EMmY trial

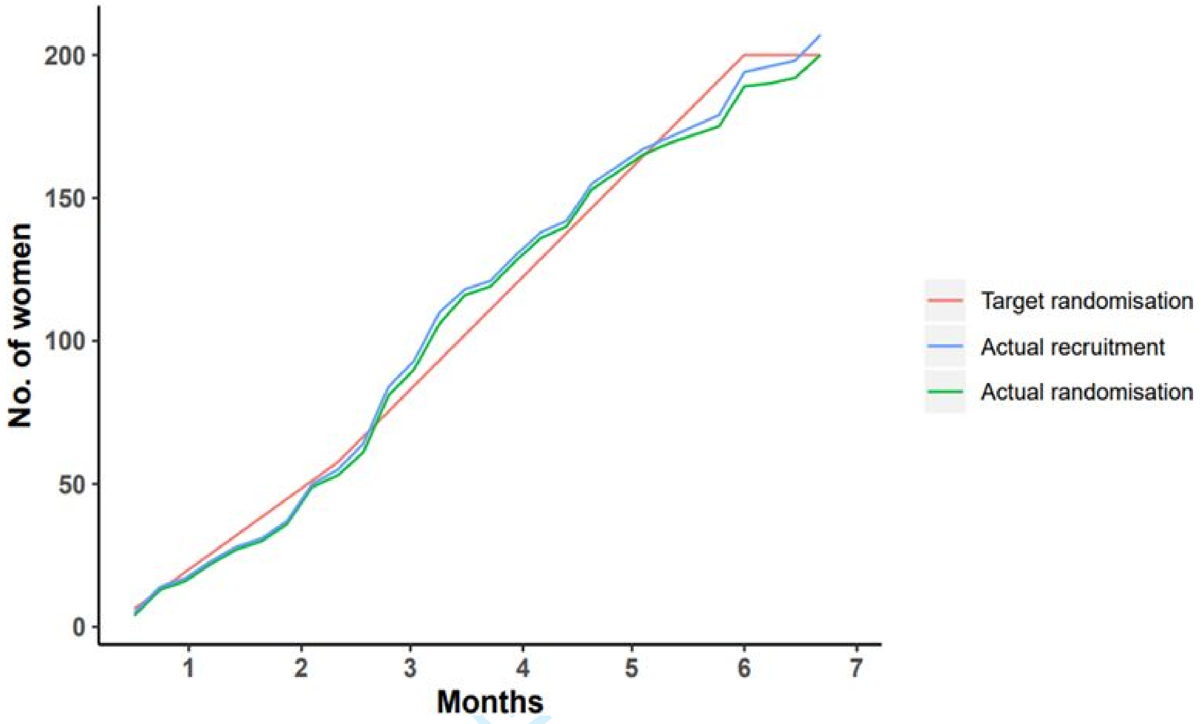

Table 1 Baseline characteristics of EMmY participants randomised to *myo*-inositol vs placebo

**Baseline characteristics**	**Intervention (*Myo*-inositol) N=99 n (%) or Mean(SD)**	**Control (Placebo) N=99 N (%) or Mean(SD)**
**Demography**		
Age (years)	31.4 (5.8)	31.9 (5.6)
Gestational age at recruitment (weeks)	12.4 (1.3)	12.3 (1.5)
BMI (kg/m^2)	28.2 (6.4)	27.9 (5.6)
Higher education	73 (73.7%)	76 (76.8%)
**Ethnicity**		
White	25 (25.3%)	40 (40.4%)
Asian	49 (49.5%)	48 (48.5%)
Black	16 (16.2%)	8 (8.1%)
Mixed/others	9 (9.1%)	3 (3%)
**Risk factors for GDM**		
GDM in previous pregnancy	8 (8.1%)	11 (11.1%)
Obesity (BMI ≥30 kg/m^2)	37 (37.4%)	36 (36.4%)
Minority Ethnic origin	72 (72.7%)	59 (59.6%)
Polycystic Ovary Syndrome	9 (9.1%)	11 (11.1%)
Previous macrosomic baby (>4.5kg)	4 (4%)	2 (2%)
Family history of diabetes (1st degree)	60 (60.6%)	59 (59.6%)
**Current pregnancy**		
Parity	0.8 (1)	0.9 (0.9)
Nulliparous	50 (50.5%)	40 (40.4%)
Alcohol use	1 (1%)	0 (0%)
Tobacco use	1 (1%)	3 (3%)
Current use of supplements	85 (85.9%)	89 (89.9%)
Current use of medication	24 (24.2%)	18 (18.2%)
Aspirin	8 (8.1%)	7 (7.1%)
**General clinical history**		
*Pre-existing medical condition	4 (4%)	4 (4%)
Family history of raised lipids (1st degree)	7 (7.1%)	8 (8.1%)

*GDM- Gestational Diabetes Mellitus, BMI- Body Mass Index; *Pre-existing medical conditions include autoimmune disease, blood/clotting disorder, cardiac problems, liver disease, lung disease, chronic hypertension, and renal disease.*

For peer review only

Table 2 Glycaemia related outcomes in the intervention and control arms of the EMmY trial

Outcomes (unit) (intervention;control)*	Intervention (*myo*-inositol) Mean (SD)	Control (placebo) Mean (SD)	MD (95% CI)
OGTT fasting (mmol/l) (81;81)	4.5 (0.5)	4.6 (0.4)	-0.0 (-0.2;0.1)
OGTT 2 hours (mmol/l) (80;81)	6.1 (2)	6.0 (1.3)	0.0 (-0.5;0.6)
HOMA-IR (50;62)	2.13 (1.32)	2.8 (2.05)	**-0.6 (-1.2;0.0)**
Insulin (mlU/L) (53;63)	10.63 (6.11)	13.72 (8.89)	**-2.69 (-5.26; -0.18)**
Leptin (µg/L) (53;63)	42.7 (21.6)	38.3 (17.1)	4.4 (-2.7;11.4)
Adiponectin (mg/L) (53;63)	6.6 (3.5)	6.1 (3.8)	0.5 (-0.9;1.8)
Urinary inositol (mg/L) (53;65)	265.7 (209.7)	194.7 (118.7)	70.76 (11.2;130.8)
c-peptide (µg/L) (53;63)	1538.5 (679.6)	1804.3 (807.8)	-253.6 (-522.7;10.4)
c-peptide in cord blood (µg/L) (10;6)	457 (268.04)	535 (296.02)	-101.74 (-358.17; 202.17)

**Number of women in the intervention and control group respectively; MD – Mean Difference*

For peer review only

Table 3 Maternal and perinatal clinical outcomes in the EMmY study

Outcomes	Intervention (*myo*-inositol) N = 99 n (%)	Control (placebo) N = 99 n (%)
**Maternal**		
GDM (IADPSG) by 28 weeks	14 (14.1%)	13 (13.1%)
GDM (NICE) by 28 weeks	14 (14.1%)	9 (9.1%)
GDM (all definitions) by delivery	24 (24.2%)	20 (20.2%)
Pre-eclampsia	3 (3%)	9 (9.1%)
Hyperemesis	3 (3%)	3 (3%)
3rd/ 4th degree tear	2 (2%)	1 (1%)
Postpartum haemorrhage	17 (17.2%)	15 (15.2%)
GA at delivery (weeks; mean SD) (88;89)*	38.9 (3.2)	38.8 (2.8)
Preterm delivery (<37 weeks)	6 (6%)	10 (10.2%)
Spontaneous vaginal delivery	44 (44%)	45 (45.9%)
**Perinatal**		
Birth weight (g; mean SD) (87;88)*	3260 (533.3)	3251 (598)
Apgar score at 10 min (69;70)*	10 (0.5)	10 (0.8)
Respiratory distress syndrome	1 (1%)	1 (1%)
Large for gestational age	6 (6.1%)	11 (11.2%)
Small for gestational age	18 (18.4 %)	22 (22.4 %)
Admission to neonatal unit	5 (5.1%)	3 (3%)

**Number of women in the intervention and control group respectively; GDM- Gestational Diabetes Mellitus, NICE- National Institute for Health and Care Excellence, IADPSG- International Association of Diabetes and Pregnancy Study Groups, GA- Gestational Age,*

Appendix 1 Clinical maternal and perinatal outcomes assessed in the EMmY study.

**Clinical outcomes**	
**Maternal**	**Fetal and neonatal**
Gestational diabetes	Hypocalcaemia
Pre-eclampsia	Hypoglycaemia
Postpartum haemorrhage	Birth weight
Gestational age at delivery	Macrosomia (birth weight >4.5Kg),
Preterm delivery (< 34 and < 37 weeks)	small for gestational age (<10th centile)
Mode of delivery	Large for gestational age (>90th Centile)
Perineal trauma	Respiratory distress syndrome
Admission to the ITU	Shoulder dystocia
Admission to HDU	Apgar score at 10 minutes
Maternal infection	Birth trauma
Maternal death	Hyperbilirubinaemia
	Septicaemia
	Admission to NICU
	Still birth
	Neonatal death.

NB: Gestational diabetes is diagnosed based on the National Institute for Care and Excellence (NICE) and International Association of Diabetes and Pregnancy Study Groups (IADPSG) criteria.
ITU - intensive care unit, HDU - High dependency unit, NICU - neonatal intensive care unit.

Appendix 2 Criteria for progression to a full-scale trial

**Feasibility objectives and related data to be collected**	**GREEN zone**	**AMBER zone**	**RED zone**
i) Consent rates of eligible women	At least 25% of eligible women agreeing to participate.	Rate between 11 and 24% women agreeing to participate	Rate <10% of eligible women agreeing to participate
ii) Attrition rate after randomisation by 28 weeks, and by delivery (withdrawal or loss to follow up)	No more than 20% of randomised women lost or withdrawn	21-50% of randomised women lost or withdrawn	More than 50% of randomised women lost or withdrawn
iii) Rate of women who take more than 75% of sachets by 28 weeks and by delivery (of those who remain on the study)	Adherence to allocated treatment in >80% of women remaining on the study.	Adherence to allocated treatment in between 51-79% of women remaining on the study.	Adherence to allocated treatment in <50% of women
iv) Collection of data on clinical outcomes.	Complete data available of >80% of study sample.	Missing data between 21-50% of study sample.	Data missing of >50% of study sample.

Appendix 3 Characteristics of the 15 women interviewed using pseudonyms

Age (years)	Ethnicity	Educational level	History of GDM (Y/N)	Adherence level (%)
≤ 30	Middle Eastern	Higher	N	50-75
> 30	South Asian	Higher	Y	>75
> 30	Middle Eastern	Higher	N	>75
> 30	White European	Higher	Y	>75
≤ 30	White European	Secondary	N	<50
> 30	South Asian	Higher	N	>75
> 30	South Asian	Higher	Y	>75

≤ 30	Black African/ Caribbean	Higher	N	<50
> 30	South Asian	Higher	N	>75
≤ 30	South Asian	Secondary	N	<50
> 30	White European	Higher	N	<50
≤ 30	White European	Secondary	N	>75
> 30	White European	Higher	N	>75
≤ 30	White European	Higher	N	<50
> 30	Middle Eastern	Higher	Y	>75

Appendix 4 Proportion of screened, recruited and randomised women in each participating site

Sites	Women screened (N)	Eligible of those screened n(%)	Recruited of those eligible n(%)	Randomised of those recruited n(%)
MRI	85	78 (91.8%)	46 (59%)	46 (100%)
NUH	144	115 (79.9%)	22 (19.1%)	22 (100%)
RLH	320	192 (60%)	53 (27.6%)	49 (92.5%)
STH	177	145 (81.9%)	41 (28.3%)	38 (92.7%)
WXH	600	243 (40.5%)	43 (17.7%)	43 (100%)
Overall	1326	773 (58.3%)	205	198

NB: MRI- Manchester Royal Infirmary (St Mary's Hospital) in Manchester, NUH- Newham Hospital, London, RLH- Royal London Hospital, London, STH- St George's Hospital, London, WXH- Whipps Cross Hospital, London.

For peer review only

Appendix 5 Impact of adherence at 28 weeks on glycaemia related outcomes

**Glycaemia related outcomes (unit) (intervention; control)***			
**≥ 50%**			
OGTT fasting (mmol/l) **(**40;40**)**	4.57 (0.6)	4.57 (0.5)	0.00 (0.2;0.2)
OGTT 2 hours (mmol/l) (39;40)	6.15 (2.2)	6.27 (1.5)	-0.11 (-0.9;0.7)
C-peptide (µg/L) (53;63)	1608.65 (723.2)	1907.71 (868.4)	-288.79 (-644.1;59.7)
Leptin (µg/L) (53;63)	42.81 (23.4)	39.31 (15.0)	4.09 (-4.8;12.7)
Insulin (mlU/L) (37;35)	11.23 (6.74)	14.81 (9.67)	**-3.34 (-6.61; -0.12)**
Adiponectin (mg/L) (53;63)	6.5 (3.6)	6.16 (3.6)	0.21 (-1.3;2.0)
Urinary inositol (mg/L) (53;65)	261 (164.6)	191.02 (117.7)	69.52 (3.2;136.8)
HOMA-IR (50;62)	2.25 (1.4)	3.06 (2.3)	**-0.8 (-1.6;-0.0)**
**≥ 75%**			
OGTT fasting (mmol/l) (34;32**)**	4.58 (0.6)	4.58 (0.5)	0.00 (0.3;0.3)
OGTT 2 hours (mmol/l) (33;32)	6.12 (2.35)	6.22 (1.53)	-0.1 (-1;0.9)
C-peptide (µg/L) (31;28)	1517.42 (573.9)	1937.86 (932.9)	**-425.4 (-808.8; -45.6)**
Leptin (µg/L) (31;28)	41.92 (24.4)	37.51 (11.9)	4.8 (-5.6;14.4)
Insulin (mlU/L) (31;28)	10.75 (5.78)	15.15 (10.4)	-4.57 (-8.2;0.99)
Adiponectin (mg/L) (31;28)	6.57 (3.6)	5.7 (3.4)	0.87 (-0.9;2.7)
Urinary inositol (mg/L) (32;27)	249.41 (152.7)	189.17 (110.3)	57.2 (-8.7;129.1)
HOMA-IR (28;27)	2.12 (1.1)	3.12 (2.5)	**-1.07 (-2; -0.2)**

NB: *GDM- Gestational Diabetes Mellitus, NICE- National Institute for Health and Care Excellence, IADPSG- International Association of Diabetes and Pregnancy Study Groups, GA- Gestational Age*
**Number of women in the intervention and control group respectively.*

Appendix 6 Costs and QALYS in both intervention and control groups

**Resource use costs**	**Intervention (*myo*-inositol) (£)**	**Control (placebo) (£)**

Supplements	2527	492
Laboratory tests	518	574
- Average costs (n)	5.51 (94)	6.17 (93)
Clinic visits	31,815	39,995
- Average costs per woman (n. of visits)	321.4 (390)	404 (389)
Adverse events	n/a	n/a
Delivery	309,268	303,753
- Average costs per woman (n)	3,514 (88)	3,472 (89)
**Total costs**	**312,121**	**344,814**
QALYs		
- Average QALY per woman (n)	0.51 (56)	0.53 (62)
**Total QALYs**	**28.4**	**32.6**

NB: QALYs – Quality Adjusted Life Years

Appendix 7 Completeness of clinical outcome data

**Proportion of data missing**			
**Clinical outcomes**	**Intervention (Myo-inositol) N = 99 n/N (%)**	**Control (Placebo) n = 99 n/N (%)**	**Total n = 198 n/N (%)**
GDM (NICE)- 28 weeks	14 (14.1%)	17 (17.2%)	31 (15.7%)
GDM (IADPSG)- 28 weeks	15 (15.2%)	17 (17.2%)	32 (16.2%)
GDM (all definitions)- by delivery	8 (8.1%)	7 (7.1%)	15 (7.6%)

***NB**: some GDM diagnosis were made between 28 weeks gestation and delivery, so would be missing at 28 weeks but not at delivery*

For peer review only

CONSORT 2010 checklist of information to include when reporting a pilot or feasibility trial*

Section/Topic	Item No	Checklist item	Reported on page No
**Title and abstract**			
	1a	Identification as a pilot or feasibility randomised trial in the title	1
	1b	Structured summary of pilot trial design, methods, results, and conclusions (for specific guidance see CONSORT abstract extension for pilot trials)	3
**Introduction**			
Background and objectives	2a	Scientific background and explanation of rationale for future definitive trial, and reasons for randomised pilot trial	5
	2b	Specific objectives or research questions for pilot trial	5
**Methods**			
Trial design	3a	Description of pilot trial design (such as parallel, factorial) including allocation ratio	6
	3b	Important changes to methods after pilot trial commencement (such as eligibility criteria), with reasons	n/a
Participants	4a	Eligibility criteria for participants	6
	4b	Settings and locations where the data were collected	6
	4c	How participants were identified and consented	7-8
Interventions	5	The interventions for each group with sufficient details to allow replication, including how and when they were actually administered	6
Outcomes	6a	Completely defined prespecified assessments or measurements to address each pilot trial objective specified in 2b, including how and when they were assessed	6-7
	6b	Any changes to pilot trial assessments or measurements after the pilot trial commenced, with reasons	n/a
	6c	If applicable, prespecified criteria used to judge whether, or how, to proceed with future definitive trial	8
Sample size	7a	Rationale for numbers in the pilot trial	7
	7b	When applicable, explanation of any interim analyses and stopping guidelines	n/a
Randomisation:			
Sequence generation	8a	Method used to generate the random allocation sequence	7
	8b	Type of randomisation(s); details of any restriction (such as blocking and block size)	7-8
Allocation concealment mechanism	9	Mechanism used to implement the random allocation sequence (such as sequentially numbered containers), describing any steps taken to conceal the sequence until interventions were assigned	7-8

For peer review only

Implementation	10	Who generated the random allocation sequence, who enrolled participants, and who assigned participants to interventions	7-8
Blinding	11a	If done, who was blinded after assignment to interventions (for example, participants, care providers, those assessing outcomes) and how	8
	11b	If relevant, description of the similarity of interventions	6
Statistical methods	12	Methods used to address each pilot trial objective whether qualitative or quantitative	8-9
**Results**			
Participant flow (a diagram is strongly recommended)	13a	For each group, the numbers of participants who were approached and/or assessed for eligibility, randomly assigned, received intended treatment, and were assessed for each objective	10
	13b	For each group, losses and exclusions after randomisation, together with reasons	10
Recruitment	14a	Dates defining the periods of recruitment and follow-up	10
	14b	Why the pilot trial ended or was stopped	n/a
Baseline data	15	A table showing baseline demographic and clinical characteristics for each group	10
Numbers analysed	16	For each objective, number of participants (denominator) included in each analysis. If relevant, these numbers should be by randomised group	10-11
Outcomes and estimation	17	For each objective, results including expressions of uncertainty (such as 95% confidence interval) for any estimates. If relevant, these results should be by randomised group	10-11
Ancillary analyses	18	Results of any other analyses performed that could be used to inform the future definitive trial	12-15
Harms	19	All important harms or unintended effects in each group (for specific guidance see CONSORT for harms)	11
	19a	If relevant, other important unintended consequences	n/a
**Discussion**			
Limitations	20	Pilot trial limitations, addressing sources of potential bias and remaining uncertainty about feasibility	15-16
Generalisability	21	Generalisability (applicability) of pilot trial methods and findings to future definitive trial and other studies	17
Interpretation	22	Interpretation consistent with pilot trial objectives and findings, balancing potential benefits and harms, and considering other relevant evidence	16-17
	22a	Implications for progression from pilot to future definitive trial, including any proposed amendments	17-18
**Other information**			
Registration	23	Registration number for pilot trial and name of trial registry	3
Protocol	24	Where the pilot trial protocol can be accessed, if available	n/a
Funding	25	Sources of funding and other support (such as supply of drugs), role of funders	21
	26	Ethical approval or approval by research review committee, confirmed with reference number	21

For peer review only

Citation: Eldridge SM, Chan CL, Campbell MJ, Bond CM, Hopewell S, Thabane L, et al. CONSORT 2010 statement: extension to randomised pilot and feasibility trials. BMJ. 2016;355.
*We strongly recommend reading this statement in conjunction with the CONSORT 2010, extension to randomised pilot and feasibility trials, Explanation and Elaboration for important clarifications on all the items. If relevant, we also recommend reading CONSORT extensions for cluster randomised trials, non-inferiority and equivalence trials, non-pharmacological treatments, herbal interventions, and pragmatic trials. Additional extensions are forthcoming: for those and for up to date references relevant to this checklist, see www.consort-statement.org.

For peer review only

BMJ Open

Myo-inositol nutritional supplement for prevention of gestational diabetes (EMmY): a randomised, placebo-controlled, double-blind pilot trial with nested qualitative study

Journal:	*BMJ Open*
Manuscript ID	bmjopen-2021-050110.R1
Article Type:	Original research
Date Submitted by the Author:	13-Oct-2021

Complete List of Authors:	Amaefule, Chiamaka; Queen Mary University of London Barts and The London School of Medicine and Dentistry, BARC (Barts Research Centre for Women's Health), Institute of Population Health Sciences<br>Drymoussi, Zoe; Queen Mary University of London Barts and The London School of Medicine and Dentistry, (BARC) Barts Research Centre for Women's Health, Institute for Population Health Sciences<br>Gonzalez Carreras , Francisco; Queen Mary University of London Barts and The London School of Medicine and Dentistry, BARC (Barts Research Centre for Women's Health), Institute of Population Health Sciences<br>Pardo Llorente , Maria del Carmen; Complutense University of Madrid, Department of Statistics and Operational Research<br>Lanz, Doris; Queen Mary University of London Barts and The London School of Medicine and Dentistry, BARC (Barts Research Centre for Women's Health), Institute of Population Health Sciences<br>Dodds, Julie; Queen Mary University of London Barts and The London School of Medicine and Dentistry, BARC (Barts Research Centre for Women's Health), Institute of Population Health Sciences<br>Sweeney, Lorna; University of East London, Institute for Health and Human Development<br>Pizzo, Elena; University College London, NIHR ARC (Applied Health Collaborations) for North Thames London, Department of Applied Health Research<br>Thomas, Amy; Queen Mary University of London Barts and The London School of Medicine and Dentistry, BARC (Barts Research Centre for Women's Health), Institute of Population Health Sciences; The Royal London Hospital, Women's and Children's Health, Barts Health NHS Trust<br>Heighway, James; Queen Mary University of London Barts and The London School of Medicine and Dentistry, BARC (Barts Research Centre for Women's Health), BARC (Barts Research Centre for Women's Health), Institute of Population Health Sciences<br>Daru, Jahnavi; Queen Mary University of London Barts and The London School of Medicine and Dentistry, (BARC) Barts Research Centre for Women's Health, Institute for Population Health Sciences<br>Sobhy, Soha ; Queen Mary University of London Barts and The London School of Medicine and Dentistry, (BARC) Barts Research Centre for Women's Health, Institute for Population Health Sciences; The Royal London Hospital, Women's and Children's Health, Barts Health NHS Trust<br>Poston, Lucilla; King's College London, Department of Women and

	Children’s Health, Faculty of Life Sciences and Medicine<br>Khalil, Asma; St George's Hospital, Department of Obstetrics and Gynaecology; St George's University of London, Vascular Biology Research Centre, Molecular and Clinical Sciences Research Institute<br>Myers, Jenny; The University of Manchester, Maternal and Fetal Health Research Centre, Manchester Academic Health Science Centre<br>Harden, Angela; University of East London, Institute for Health and Human Development<br>Hitman, Graham; Queen Mary University of London Barts and The London School of Medicine and Dentistry, Centre for Genomics and Child Health, Blizard Institute<br>Khan , Khalid; Universidad de Granada, Department of Preventive Medicine and Public Health<br>Zamora, Javier; IRYCIS, Clinical Biostatistics Unit and CIBER Epidemiology and Public Health; University of Birmingham, WHO Collaborating Centre for Global Women’s Health, Institute of Metabolism and Systems Research<br>Pérez, Teresa ; Queen Mary University of London Barts and The London School of Medicine and Dentistry, BARC (Barts Research Centre for Women’s Health), Institute of Population Health Sciences; Complutense University of Madrid, Department of Statistics and Data Science<br>Huda, Mohammed SB; The Royal London Hospital Diabetes Diabetes and Metabolism, Barts Health NHS Trust<br>Thangaratinam, Shakila; University of Birmingham, WHO Collaborating Centre for Global Women’s Health, Institute of Metabolism and Systems Research; Birmingham Women's and Children's NHS Foundation Trust
**Primary Subject Heading**:	Obstetrics and gynaecology
Secondary Subject Heading:	Qualitative research, Diabetes and endocrinology
Keywords:	Diabetes in pregnancy < DIABETES & ENDOCRINOLOGY, HEALTH ECONOMICS, Maternal medicine < OBSTETRICS, QUALITATIVE RESEARCH, PUBLIC HEALTH

SCHOLARONE™
Manuscripts

I, the Submitting Author has the right to grant and does grant on behalf of all authors of the Work (as defined in the below author licence), an exclusive licence and/or a non-exclusive licence for contributions from authors who are: i) UK Crown employees; ii) where BMJ has agreed a CC-BY licence shall apply, and/or iii) in accordance with the terms applicable for US Federal Government officers or employees acting as part of their official duties; on a worldwide, perpetual, irrevocable, royalty-free basis to BMJ Publishing Group Ltd ("BMJ") its licensees and where the relevant Journal is co-owned by BMJ to the co-owners of the Journal, to publish the Work in this journal and any other BMJ products and to exploit all rights, as set out in our licence.

The Submitting Author accepts and understands that any supply made under these terms is made by BMJ to the Submitting Author unless you are acting as an employee on behalf of your employer or a postgraduate student of an affiliated institution which is paying any applicable article publishing charge ("APC") for Open Access articles. Where the Submitting Author wishes to make the Work available on an Open Access basis (and intends to pay the relevant APC), the terms of reuse of such Open Access shall be governed by a Creative Commons licence – details of these licences and which Creative Commons licence will apply to this Work are set out in our licence referred to above.

Other than as permitted in any relevant BMJ Author's Self Archiving Policies, I confirm this Work has not been accepted for publication elsewhere, is not being considered for publication elsewhere and does not duplicate material already published. I confirm all authors consent to publication of this Work and authorise the granting of this licence.

For peer review only

Myo-inositol nutritional supplement for prevention of gestational diabetes (EMmY): a randomised, placebo-controlled, double-blind pilot trial with nested qualitative study

Chiamaka Esther Amaefule[1] Email: c.e.amaefule@qmul.ac.uk; Zoe Drymoussi[1] Email: zdrymoussi@gmail.com; Francisco Jose Gonzalez Carreras [1] Email: f.j.gonzalez.carreras@gmail.com; Maria del Carmen Pardo Llorente [1,2] Email: mcapardo@mat.ucm.es; Doris Lanz[1] Email: d.lanz@qmul.ac.uk; Julie Dodds[1] Email: jpdodds247@gmail.com; Lorna Sweeney[3] Email: lorna.christine@gmail.com; Elena Pizzo[4] Email: e.pizzo@ucl.ac.uk; Amy Thomas[1,5] Email: amy.thomas28@nhs.net; James Heighway[1] Email: j.heighway@qmul.ac.uk; Jahnavi Daru[1] Email: j.daru@qmul.ac.uk; Soha Sobhy[1,5] Email: soha.sobhy@qmul.ac.uk; Lucilla Poston[6] Email: lucilla.poston@kcl.ac.uk; Asma Khalil[7,8] Email: akhalil@sgul.ac.uk; Jenny Myers[9] Email: Jenny.Myers@manchester.ac.uk; Angela Harden[3] Email: a.harden@uel.ac.uk; Graham A Hitman[10] Email: g.a.hitman@qmul.ac.uk; Khalid Saeed Khan[11] Email: profkkhan@gmail.com; Javier Zamora [12, 13] Email: javier.zamora@hrc.es; Teresa Pérez [1,14] Email: teperez@estad.ucm.es; Mohammed SB Huda[15] Email: bobby.huda1@nhs.net; Shakila Thangaratinam [13,16]* Email: s.thangaratinam.1@bham.ac.uk

* Corresponding author

[1] BARC (Barts Research Centre for Women's Health), Institute of Population Health Sciences, Barts and the London School of Medicine and Dentistry, Queen Mary University of London, London, UK

[2] Department of Statistics and Operational Research, Complutense University of Madrid, Madrid Spain.

[3]Institute for Health and Human Development, University of East London, London, UK

[4] NIHR ARC (Applied Research Collaborations) for North Thames London, Department of Applied Health Research, University College London, London, UK.

For peer review only - http://bmjopen.bmj.com/site/about/guidelines.xhtml

[5]Women's and Children's Health, Royal London Hospital, Barts Health NHS Trust, London UK.

[6] Department of Women and Children's Health, Faculty of Life Sciences and Medicine, King's College London, London UK.

[7] Department of Obstetrics and Gynaecology, St George's Hospital, London UK.

[8] Vascular Biology Research Centre, Molecular and Clinical Sciences Research Institute, St George's University of London, Cranmer Terrace, London UK.

[9] Maternal and Fetal Health Research Centre, University of Manchester, Manchester Academic Health Science Centre, UK.

[10] Centre for Genomics and Child Health, Blizard Institute, Barts and the London School of Medicine and Dentistry, Queen Mary University of London, London, UK.

[11] Department of Preventive Medicine and Public Health, Universidad de Granada, Granada, Spain.

[12] Clinical Biostatistics Unit (IRYCIS) and CIBER Epidemiology and Public Health, Madrid, Spain.

[13] WHO Collaborating Centre for Global Women's Health, Institute of Metabolism and Systems Research, University of Birmingham, Birmingham, UK.

[14] Department of Statistics and Data Science, Complutense University of Madrid, Madrid, Spain.

[15] Department of Diabetes & Metabolism, Barts Health NHS Trust, Royal London Hospital, London, UK.

[16] Birmingham Women's and Children's NHS Foundation Trust, Birmingham, UK.

WORD COUNT: 4076

For peer review only

ABSTRACT

Objectives: To determine the feasibility and acceptability of conducting a randomised trial on the effects of *myo*-inositol in preventing gestational diabetes in high-risk pregnant women.

Design: A multicentre, double-blind, placebo-controlled, pilot randomised trial with nested qualitative evaluation.

Setting: Five inner city UK National Health Service (NHS) hospitals

Participants: Multi-ethnic pregnant women at 12^{+0} and 15^{+6} weeks' gestation with risk factors for gestational diabetes.

Interventions: 2g of *myo*-inositol or placebo, both included 200µg folic acid, twice daily until delivery.

Primary outcome measures: Rates of recruitment, randomisation, adherence and follow-up.

Secondary outcome measures: Glycaemic indices (including homeostatic model assessment-insulin resistance HOMA-IR), gestational diabetes (diagnosed using oral glucose tolerance test at 28 weeks and by delivery), maternal, perinatal outcomes, acceptability of intervention, and costs.

Results: Of the 1326 women screened, 58% (773/1326) were potentially eligible, and 27% (205/773) were recruited. We randomised 97% (198/205) of all recruited women (99 each in intervention and placebo arms), and ascertained outcomes in 90% of women (178/198) by delivery. The mean adherence was 52% (SD 44) at 28weeks' and 34% (SD 41) at 36 weeks' gestation. HOMA-IR and serum insulin levels were lower in the myo-inositol vs placebo arm (Mean Difference -0.6, 95% CI -1.2 to 0.0 and -2.69, 95% CI -5.26 to -0.18 respectively). The study procedures were acceptable to women and healthcare professionals. Women who perceived themselves at high risk of gestational diabetes were more likely to participate and adhere to the intervention. The powder form of *myo*-inositol and placebo, along with nausea in pregnancy were key barriers to adherence.

For peer review only

Conclusions: A future trial on *myo*-inositol vs placebo to prevent gestational diabetes is feasible. The intervention will need to be delivered in a non-powder form to improve adherence. There is a signal for efficacy in reducing insulin resistance in pregnancy with *myo*-inositol.

Trial registration: ISRCTN48872100

Funding: Funded by Barts Charity, grant number MGU0373

Key words: diabetes in pregnancy, maternal medicine, qualitative research, public health, health economics

Abbreviations: CONSORT (Consolidated Standards of Reporting Trials); EQ-5D-5L (European Quality of life 5-Dimensions 5-Level scale); gestational diabetes; IADPSG (International Association of Diabetes and Pregnancy Study Groups); LGA (Large for Gestational Age); LTFU (Lost To Follow-Up); NHS (National Health Service); NICE (National Institute for Health and Care Excellence); NIHR (National Institute for Health Research); QALY (Quality-Adjusted Life-Year)

STRENGTHS AND LIMITATIONS

- The EMmY study is the first multi-centre trial involving ethnically diverse high-risk women from inner city NHS hospitals in the UK, looking at *myo*-inositol vs placebo during pregnancy.
- It is feasible to complete a full scale RCT on the effect of *myo*-inositol vs placebo in preventing gestational diabetes in high-risk women.
- Women do not prefer the powder of the intervention and may find a tablet form more acceptable.
- *Myo*-inositol use in pregnancy may reduce insulin resistance.

For peer review only

For peer review only - http://bmjopen.bmj.com/site/about/guidelines.xhtml

- If *myo*-inositol is shown to prevent gestational diabetes, this simple intervention could be easily implemented in clinical practice to improve maternal and perinatal outcomes.

For peer review only

For peer review only - http://bmjopen.bmj.com/site/about/guidelines.xhtml

INTRODUCTION

Primary prevention of gestational diabetes, a condition with high glucose levels first identified in pregnancy, is key to minimising maternal and perinatal complications associated with the condition.[1] Prevention of gestational diabetes can also reduce the risk of type 2 diabetes in mothers and their children in the long term. [2,3] Simple, easy to administer, and acceptable interventions are needed to prevent gestational diabetes.

Myo-inositol, a nutritional supplement that is present in fruits and fibre rich food and available as over-the-counter nutritional supplement, has the potential to prevent gestational diabetes through its insulin sensitising action. Few small trials have shown a promising role for *myo*-inositol with reductions in rates of gestational diabetes by up to 60%.[4, 5] But, in addition to small sample sizes (< 300 women), these trials were from a single country (Italy) and involved only Caucasian women.[5] The generalisability of these findings to high risk multi-ethnic populations who are most at risk of gestational diabetes is not known. A Cochrane review has called for further large trials on the effects of *myo*-inositol on gestational diabetes and complications in multi-ethnic populations. We also need to determine the costs and health service use associated with the intervention.[6]

Prior to undertaking a full-scale definitive trial on the effect of *myo*-inositol in preventing gestational diabetes, which requires substantial resources, we need to assess the feasibility of conducting such a trial beforehand. We undertook a pilot randomised trial comparing *myo*-inositol vs placebo, with a nested qualitative study, to assess the feasibility of the trial design, explore the acceptability and contextual issues around intervention delivery and trial procedures, and the potential effects on glycaemic, maternal, perinatal, cost, and quality of life outcomes.

For peer review only - http://bmjopen.bmj.com/site/about/guidelines.xhtml

For peer review only

METHODS

The EMmY trial was a multicentre, randomised, placebo-controlled, double-blind pilot trial with nested qualitative study and economic evaluation conducted with a prospective protocol and reported using CONSORT (Consolidated Standards of Reporting Trials) guidelines.[7] The full details of the study protocol are published elsewhere.[8]

Participants and setting

We screened pregnant women attending five inner city maternity units in London and Manchester. Women were eligible for recruitment if they were at least 16 years of age with a viable singleton pregnancy between 12^{+0} and 15^{+6} weeks' gestation, were able to provide written informed consent in English, and were considered to be at high risk of gestational diabetes as per the NICE (National Institute for health and Care Excellence) criteria.[9] We excluded women with type 2 diabetes, and those taking metformin or corticosteroids. Recruited women with previous history of gestational diabetes underwent additional testing with OGTT (oral glucose tolerance test) or HbA1c, and were randomised after ruling out undiagnosed type 2 diabetes (OGTT fasting ≥5.6 mmol/l, 2hour glucose ≥7.8 mol/l or HbA1c ≥48 mmol/mol).

Intervention and control group allocation

The intervention group were provided with *myo*-inositol 2g supplement with 200µg folic acid, to be taken twice a day in a powder form mixed in water, approximately 1 hour before or after a meal. The placebo was an identical looking and tasting powder of Xylitol filler with 200µg folic acid to be taken twice a day.

Outcomes

The primary outcomes were the proportion of screened women who were eligible, the proportion of eligible women who were recruited and randomised, rates of adherence and follow-up. The

For peer review only - http://bmjopen.bmj.com/site/about/guidelines.xhtml

secondary outcomes were acceptability of the intervention and trial to women and health professionals; laboratory outcomes related to glycaemia such as fasting and 2-hour plasma glucose levels in 75g OGTT, HOMA-IR, serum insulin, adiponectin, leptin and urinary inositol at 28 weeks' gestation, and maternal and cord blood c-peptide at delivery. Other secondary outcomes included diagnosis of gestational diabetes at 28 weeks' gestation by modified IADPSG (International Association of Diabetes and Pregnancy Study Groups) and NICE criteria, maternal and perinatal morbidity and mortality (Appendix 1). The economic outcomes included costs (NHS health and social care resources use) and quality of life. Other process outcomes included deviations from study protocol, completeness of data collection and the level of support required by the site team for trial delivery.

Sample size

We expected that 1500 women would be booked for antenatal care each month at the participating hospitals, and at least 300 of those would be eligible. Assuming 1000 eligible women were approached, we expected 25% (250/1000) to be consented. We expected that 20% (50) of these would be women with a previous history of gestational diabetes, with abnormal HbA1C and/ or OGTT and would be excluded. This would result in 200 women randomised.

Study conduct

We approached pregnant women at booking and screened them for eligibility. After obtaining written informed consent from eligible women, we collected baseline information on demographic and clinical characteristics, and women completed the validated European Quality of life 5-Dimensions 5-Level scale (EQ-5D-5L) questionnaire on quality of life measures. Women were randomised between 12^{+0} and 15^{+6} weeks' gestation using an online randomisation system (administered by epiGenesys, University of Sheffield) to either the *myo*-inositol or placebo treatment arm. We used a randomisation scheme based on permuted blocks of random

For peer review only

For peer review only - http://bmjopen.bmj.com/site/about/guidelines.xhtml

block size (sizes 4, 6 and 8) stratified by participating site. No adaptive or minimisation strategies were used. Participants, healthcare providers and researchers were blinded to the group allocation. We followed-up women in person or by phone at 20, 28- and 36-weeks' gestation and at delivery. All women were offered OGTT at 28 weeks' gestation to screen for gestational diabetes. Women self-reported adherence to the intervention using a paper-based diary, or an optional mobile application. Where possible, we supplemented this information with a count of unused sachets. The trial was overseen monthly by a Trial Management Group and biannually by the Project Steering Committee (combining functions from a more traditional set up of a separate Data Monitoring Committee and Trial Steering Committee) reviewing trial progress and conduct. We prespecified the criteria for progression to a full-scale trial (Appendix 2).

Analysis

Using an intention-to-treat (ITT) approach, we summarised the feasibility outcomes using proportions with 95% CI (confidence interval) and other descriptive statistics. For continuous laboratory outcomes, we calculated the effect sizes (e.g. mean differences) with 95% CI. We reported the rates of dichotomous clinical outcomes and costs in the two arms of the study. The impact of a previous history of gestational diabetes and the use of the mobile app on adherence were conducted as a post-hoc exploratory analysis. All analyses were performed using R, version 3.6.1.

Qualitative study

We explored the experiences and perspectives of women and healthcare professionals on the intervention and trial procedures, using observations and interview methods. Recruitment appointments were observed to ascertain women's specific motivations or concerns regarding participating in the trial, as well as contextual factors that may optimise or discourage participation. We invited eligible women who declined trial participation to give their reasons

For peer review only - http://bmjopen.bmj.com/site/about/guidelines.xhtml

verbally to the researcher or complete an open-ended questionnaire for reasons for declining consent. We interviewed women and healthcare professionals involved in trial delivery to explore the barriers and facilitators of participation, adherence and retention in the study as well as trial delivery in interviews with health professionals. Interview data was transcribed and analysed using a thematic analytical approach.[10, 11] Purposive sampling was used to ensure the women interviewed were from diverse age groups, ethnic backgrounds, clinical characteristics and levels of adherence (Appendix 3). We contacted women who withdrew from the trial, and midwives to discuss in depth the reasons for withdrawal.

Economic evaluation

A cost-utility analysis of *myo*-inositol supplementation versus placebo "within trial" period was undertaken. The cost utility measures were the incremental cost per unit of change per Quality-Adjusted Life-Year (QALY) gained. Cost was assessed from the perspective of the NHS and personal social services. The unit costs were retrieved from published sources, calculated in pound sterling based on 2018 costs. Resource use data was collected retrospectively. For each participant, their EQ-5D-5L health state was converted to a single summary index (utility value), constructing a utility profile based on the assumption of a straight-line relation between their utility values and each measurement point. For each participant, QALYs from baseline to 28 weeks were calculated as the area under the utility profile.

Patient and Public Involvement (PPI)

We sought input from Katie's Team, a women's health and childbirth PPI advisory group[12], who contributed to the study design and patient-facing documents for the pilot trial, and the interview schedule for the qualitative study.[8] Patients and members of the public were not involved in the recruitment to and conduct of the study. We plan to disseminate results from the EMmY study to

For peer review only - http://bmjopen.bmj.com/site/about/guidelines.xhtml

participants and members of Katie's Team, who can then circulate further through their networks and social media platforms.

RESULTS

Over six months (Feb - Sep 2018), we screened 1326 women across all five participating sites, and randomised 198 women to either intervention or control arm (Figure 1). The majority of participants in both arms were of ethnic minority origin (73% intervention; 60% control), over a third were obese (37% intervention; 36% control), and around half (51% intervention; 40% control) were nulliparous. About 60% of all women had a first degree relative with type 2 diabetes, and one in ten (8% intervention; 11% control) had a history of previous gestational diabetes (Table 1). Urinary inositol levels were higher in the *myo*-inositol than the placebo arm (MD 70.76 mg/L, 95% CI 11.2 to 130.8) (Table 2).

Table 1 Baseline characteristics of EMmY participants randomised to *myo*-inositol vs placebo

**Baseline characteristics**	**Intervention (*Myo*-inositol) N=99 n (%) or Mean(SD)**	**Control (Placebo) N=99 N (%) or Mean(SD)**
**Demography**		
Age (years)	31.4 (5.8)	31.9 (5.6)
Gestational age at recruitment (weeks)	12.4 (1.3)	12.3 (1.5)
BMI (kg/m^2)	28.2 (6.4)	27.9 (5.6)
Higher education	73 (73.7%)	76 (76.8%)
**Ethnicity**		
White	25 (25.3%)	40 (40.4%)
Asian	49 (49.5%)	48 (48.5%)
Black	16 (16.2%)	8 (8.1%)
Mixed/others	9 (9.1%)	3 (3%)
**Risk factors for GDM**		
GDM in previous pregnancy	8 (8.1%)	11 (11.1%)
Obesity (BMI ≥30 kg/m^2)	37 (37.4%)	36 (36.4%)
Minority Ethnic origin	72 (72.7%)	59 (59.6%)
Polycystic Ovary Syndrome	9 (9.1%)	11 (11.1%)
Previous macrosomic baby (>4.5kg)	4 (4%)	2 (2%)
Family history of diabetes (1st degree)	60 (60.6%)	59 (59.6%)
**Current pregnancy**		
Parity	0.8 (1)	0.9 (0.9)
Nulliparous	50 (50.5%)	40 (40.4%)
Alcohol use	1 (1%)	0 (0%)
Tobacco use	1 (1%)	3 (3%)
Current use of supplements	85 (85.9%)	89 (89.9%)
Current use of medication	24 (24.2%)	18 (18.2%)
Aspirin	8 (8.1%)	7 (7.1%)
**General clinical history**		

For peer review only - http://bmjopen.bmj.com/site/about/guidelines.xhtml

*Pre-existing medical condition	4 (4%)	4 (4%)
Family history of raised lipids (1st degree)	7 (7.1%)	8 (8.1%)

*GDM- Gestational Diabetes Mellitus, BMI- Body Mass Index; *Pre-existing medical conditions include autoimmune disease, blood/clotting disorder, cardiac problems, liver disease, lung disease, chronic hypertension, and renal disease.*

Table 2 Glycaemia related outcomes in the intervention and control arms of the EMmY trial

**Outcomes (unit) (intervention;control)***	**Intervention (*myo*-inositol) Mean (SD)**	**Control (placebo) Mean (SD)**	**MD (95% CI)**
OGTT fasting (mmol/l) (81;81)	4.5 (0.5)	4.6 (0.4)	-0.0 (-0.2;0.1)
OGTT 2 hours (mmol/l) (80;81)	6.1 (2)	6.0 (1.3)	0.0 (-0.5;0.6)
HOMA-IR (50;62)	2.13 (1.32)	2.8 (2.05)	**-0.6 (-1.2;0.0)**
Insulin (mlU/L) (53;63)	10.63 (6.11)	13.72 (8.89)	**-2.69 (-5.26; -0.18)**
Leptin (µg/L) (53;63)	42.7 (21.6)	38.3 (17.1)	4.4 (-2.7;11.4)
Adiponectin (mg/L) (53;63)	6.6 (3.5)	6.1 (3.8)	0.5 (-0.9;1.8)
Urinary inositol (mg/L) (53;65)	265.7 (209.7)	194.7 (118.7)	70.76 (11.2;130.8)
c-peptide (µg/L) (53;63)	1538.5 (679.6)	1804.3 (807.8)	-253.6 (-522.7;10.4)
c-peptide in cord blood (µg/L) (10;6)	457 (268.04)	535 (296.02)	-101.74 (-358.17; 202.17)

**Number of women in the intervention and control group respectively; MD – Mean Difference*

PRIMARY OUTCOMES

Of the 773 eligible women, 27% (205/773) consented to be involved in the study. Of these, 98% (198/205) were randomised to the trial after ruling out type 2 diabetes (Figure 1). Figure 2 and Appendix 4 show the rate of recruitment and randomisation across all and individual participating sites respectively. The commonest reason for women declining consent was a lack of interest in research (48%, 271/568) (Figure 1). Outcomes were assessed in 75% (148/198) of women at 28 weeks, and 90% (178/198) of women were followed-up at delivery. The rates of adherence to the intervention in the *myo*-inositol (mean 53%, SD 45) and placebo (mean 50%, SD 43) groups were similar at 28 weeks. A sensitivity analysis showed that 75% (12/16) of women with gestational diabetes in previous pregnancies took more than 75% of *myo*-inositol sachets compared to 41% of women without a history of this condition (60/145). Six of the eight App users also took more than 75% of *myo*-inositol sachets.

SECONDARY OUTCOMES

For peer review only

Glycaemic outcomes

We found significant reductions in HOMA-IR (MD -0.6; 95% CI -1.2 to 0.0) and levels of insulin (MD -2.69; 95% CI -5.26 to -0.18) in the intervention group vs control. There were no differences between the groups in other glycaemic estimates (Table 2). Sensitivity analyses of women with over 50% and over 75% adherence to the intervention showed larger reductions in HOMA-IR and insulin levels (Appendix 5).

Clinical outcomes

The proportion of women diagnosed with gestational diabetes were 14.1% (14/99) and 13.1% (13/99) in the intervention and control arms respectively at 28 weeks by modified IADPSG criteria. The overall diagnosis of gestational diabetes by delivery were 24.2% (24/99) and 20.2% (20/99) using any criteria (Table 3). The rates of preterm birth in the *myo*-inositol and placebo groups were 6% (6/99) and 10.2% (10/99) respectively, and large-for-gestational age (LGA) were 6.1% (6/99) in intervention and 11.2% (11/99) in control arm. There were no reports of any maternal anaphylactic reaction or maternal death (Table 3).

For peer review on

Table 3 Maternal and perinatal clinical outcomes in the EMmY study

**Outcomes**	**Intervention (*myo*-inositol) N = 99 n (%)**	**Control (placebo) N = 99 n (%)**
**Maternal**		
GDM (IADPSG) by 28 weeks	14 (14.1%)	13 (13.1%)
GDM (NICE) by 28 weeks	14 (14.1%)	9 (9.1%)
GDM (all definitions) by delivery	24 (24.2%)	20 (20.2%)
Pre-eclampsia	3 (3%)	9 (9.1%)
Hyperemesis	3 (3%)	3 (3%)
3rd/ 4th degree tear	2 (2%)	1 (1%)
Postpartum haemorrhage	17 (17.2%)	15 (15.2%)
GA at delivery (weeks; mean SD) (88;89)*	38.9 (3.2)	38.8 (2.8)
Preterm delivery (<37 weeks)	6 (6%)	10 (10.2%)
Spontaneous vaginal delivery	44 (44%)	45 (45.9%)
**Perinatal**		
Birth weight (g; mean SD) (87;88)*	3260 (533.3)	3251 (598)
Apgar score at 10 min (69;70)*	10 (0.5)	10 (0.8)
Respiratory distress syndrome	1 (1%)	1 (1%)

For peer review only - http://bmjopen.bmj.com/site/about/guidelines.xhtml

Large for gestational age	6 (6.1%)	11 (11.2%)
Small for gestational age	18 (18.4 %)	22 (22.4 %)
Admission to neonatal unit	5 (5.1%)	3 (3%)

**Number of women in the intervention and control group respectively; GDM- Gestational Diabetes Mellitus, NICE- National Institute for Health and Care Excellence, IADPSG- International Association of Diabetes and Pregnancy Study Groups, GA- Gestational Age,*

Adverse outcomes

Vomiting was reported by 15.1% (15/99) of women in the intervention arm and by 9.1% (9/99) in the control arm. Other gastrointestinal symptoms were similar between groups: diarrhoea was reported by 5.1% (5/99) and 4% (4/99) in the intervention and placebo arms respectively, and constipation by 5.1% (10/198) in both groups.

Cost outcomes

Baseline EQ-5D-5L questionnaires were completed by all women randomised, and end of trial EQ-5D-5L questionnaires by 57% (56/99) in the intervention arm and 63% (62/99) in the control group. The total cost of trial (including the supplements, diagnostic tests, clinic visits and cost of birth/delivery) was very similar in both groups (£344,127 versus £344,814 in the intervention and control group respectively) and on average around £3500 per woman. The QALYs were similar in both groups with 0.53 QALYs over 28 weeks in the control and 0.51 QALYs in the intervention group. With no major difference in costs and QALYs between groups, an incremental cost-effectiveness ratio (ICER) was not calculated and further sensitivity analysis was not explored. The costs of resources used by women in both groups as well as QALYs are reported in Appendix 6.

QUALITATIVE FINDINGS

We observed the recruitment of 28 participants across three sites. Nine women who did not want to participate in the study provided input on reasons for declining consent. Fifteen women

For peer review only - http://bmjopen.bmj.com/site/about/guidelines.xhtml

consented to a qualitative research interview. Fourteen healthcare professionals were interviewed, including research midwives with responsibility for recruitment at each participating site, the academic trial coordinator, the research coordinator at participating sites and a clinical academic.

Recruitment and participation

Women responded positively in the interviews about their experience of the recruitment process. They felt they received adequate information about the study to make a decision about participating, and that their questions and concerns were addressed. From our observations and interviews with women and research midwives, perceived risk of developing gestational diabetes was identified as a key factor influencing the decision about whether or not to participate in the trial. Women were more motivated to join the study if they believed they were at risk of developing gestational diabetes in their pregnancy, either due to a previous experience with the condition in an earlier pregnancy, or due to their awareness of gestational diabetes risk factors such as family history of diabetes or weight gain in pregnancy. Women without a previous history of gestational diabetes, or a family history of diabetes and those who felt they already engaged in healthy lifestyle practices did not typically view themselves to be at risk of developing gestational diabetes and were more likely to decline consent (even if the study had identified them as at-risk due to their ethnic background).

> *"… in my first pregnancy I suffered a lot. I wasn't able to have anything…even if I had an apple…I used to climb the stairs up and down,…to…bring the sugar low…it's been a nightmare for me…I don't want that to happen again…I'm just following it religiously, just to make sure…"* (Farihah, South Asian with previous gestational diabetes)

> *"I've never had a patient with previous diabetes who declined the study."* (Midwife A, Site 1)

For peer review only - http://bmjopen.bmj.com/site/about/guidelines.xhtml

The "natural" make-up of *myo*-inositol as a vegetarian food supplement, rather than a drug, appeared to encourage trial participation. Many women felt reassured that it was safe to take the supplement in pregnancy and research midwives said it was ideal for the predominantly Muslim target population of the study, who only eat Halal foods.

> *"... It was something natural…a natural ingredient, nothing too chemical or new or anything. So it didn't worry me".* (Amber, White European with higher education level).

Nevertheless, some women cited *"fear of supplements"* on the decliners' questionnaire as their reason for declining consent and some were concerned that participating in research involved testing the effect of new drugs, which could potentially harm the baby. Partners with these concerns sometimes discouraged participation even when the woman had indicated interest in being part of the research. High staff turnover rates in most participating sites affected study conduct. The midwives, research coordinator and trial coordinator suggested an increase in staff capacity or provision of support staff during more intense periods of study recruitment and follow-up. Research midwives highlighted that recruitment success was dependent on dissemination of trial information and engagement with the wider antenatal team.

Adherence and retention

Several of the women interviewed experienced prolonged nausea and vomiting in pregnancy and discontinued the supplements when they felt these symptoms were made worse by drinking the supplement (several mouthfuls of *"sweet"*-tasting, *"sandy"*-textured liquid). Research midwives also cited pregnancy sickness as a major reason for participant non-adherence and withdrawal. A tablet form of the supplement was suggested by women and midwives as a preferable option in future studies. The twice-daily intake of the supplement and the time restrictions on taking it one hour before or after a meal were also major barriers to long-term adherence, as women struggled to embed the regimen into their busy daily routines.

For peer review only - http://bmjopen.bmj.com/site/about/guidelines.xhtml

"…if it was in tablet form it would be easier for them to just swallow it, just like the Pregnacare... But because it's in liquid they have to take a mouthful and mouthful and mouthful... And the main reason for the withdrawal again is the fact that they were having very bad morning sickness". (Midwife G, Site 4)

Notably, adherence was reduced for some women after a normal or abnormal OGTT at the 28 weeks' visit. Those with a normal result felt the supplements were no longer needed and those with an abnormal result felt the strict regimen they had been following had not been effective. This was less of an issue for women who felt most at risk of developing gestational diabetes.

We were not able to fully evaluate the use of the App, due to difficulties in the NHS Wi-Fi connectivity, which in many cases prevented the download of the app at the point of randomisation. The few women who downloaded the App felt the automated reminders were helpful with the regimen and critical to their adherence, while other participants without the app felt they would have adhered to the sachets more consistently if they had been supported with App reminders. They also felt they would have been more consistent in recording their adherence if they could have done so on their personal mobile phones via an App, rather than in a diary, which was likened to administrative work. By contrast, some women who were not as comfortable with using technology felt the diary was easier and more straightforward.

"… I think an App or something would have been better… something that reminded me to take it… I just saw the booklet, it's just like an admin thing. That didn't help me. So an App might have done because I've got all the pregnancy Apps on my phone to remind me to take other things … (Amber, in full time employment)

DISCUSSION

Summary of key findings

For peer review only - http://bmjopen.bmj.com/site/about/guidelines.xhtml

Our multicentre pilot trial showed that it is feasible to recruit, randomise, adequately follow-up, and collect the relevant outcomes in a multi-ethnic inner-city pregnant population comprising of high-risk women, to evaluate the effects of *myo*-inositol on gestational diabetes. The trial procedures are acceptable to women and healthcare professionals. The powder form of the intervention was a barrier to adherence and women requested alternative forms such as a tablet. We observed a positive signal for efficacy in the *myo*-inositol arm than placebo for insulin resistance.

Strengths and limitations

Our pilot randomised trial was based on a clear prospective protocol,[8] and we reported the findings using recommended guidelines.[7] We showed that it is feasible to recruit the required numbers of participants within planned time frames, and to cost, across multiple maternity units. More than half of our participants were from black and ethnic minority backgrounds, who are at most risk of developing gestational diabetes. We identified the barriers and facilitators to recruitment as reported by participating mothers and healthcare professionals. The trial procedures were robustly carried out, with good accuracy in data collection (Appendix 7). The outcomes were ascertained in over 80% of women in the control and intervention arms. The qualitative study identified the main factors to sub-optimal adherence, which will need to be addressed in a full-scale trial. We showed that other outcomes such as clinical and cost-related outcomes can be collected to inform the full-scale trial.

Some of the limitations to the study included varying levels of recruitment rates across sites. Something to consider when planning the full-scale trial will be to increase staffing, to ensure that sites are fully supported during intense periods of participant recruitment and follow-up. There were more women of black and ethnic minority backgrounds in the intervention than control arms, who are more likely to have dysglycaemia in pregnancy. In a future full-scale trial,

For peer review only - http://bmjopen.bmj.com/site/about/guidelines.xhtml

we plan to minimise by ethnicity to ensure both groups are fully balanced. Some of the qualitative insights suggested that adherence may have been improved if the App reminders had been made available for more women. Another limitation was not being able to assess the cord blood levels of the glycaemic markers in a larger proportion of participants.

Interpretation of findings and comparison with existing literature

Our consent rates were consistent with other pregnancy trials involving multi-ethnic groups, citing similar reasons why eligible participants declined consent.[13, 14] Previous trials on *myo*-inositol supplementation in pregnancy reported consent rates over 70% but had a predominantly Caucasian population.[15, 16] Other pregnancy trials reported similar attrition rates to the EMmY study, [15, 16] and made reference to phone-based reminders and diaries as crucial to adherence.[17, 18] The EMmY study was not powered to detect any statistical differences in clinical or laboratory outcomes, including for gestational diabetes. Hence, we refrained from reporting comparative estimates for dichotomous outcomes. We identified the expected rates of gestational diabetes in the control arm that will inform the sample size calculations for the full-scale trial. We also observed that women continued to be diagnosed with gestational diabetes beyond 28 weeks, therefore a definitive trial would need to assess onset of gestational diabetes at any time in pregnancy along with critically important clinical outcomes as main outcomes.

Clinical and research implications

Overall, the pilot study achieved 'green' status in rates of recruitment, attrition, and data collection, and 'amber' in adherence to the intervention at 28 weeks, as markers of progression to full-scale trial. Our qualitative study has identified the main reason for reduced adherence to be dissatisfaction with the powder form of the intervention. The findings were further discussed with the independent Steering Committee and Katie's Team, the patient and public involvement

For peer review only

support arm of the study, who recommended use of *myo*-inositol tablets instead of powder when progressing to a full-scale trial.

We explored the differences in the continuous laboratory outcomes for signals of efficacy, as these would inform the choice of main outcomes for the full-scale trial. Despite lower than anticipated overall adherence to the intervention, the higher levels of urinary inositol in the intervention group makes the comparison of outcomes valid. In randomised trials on *myo*-inositol in pregnancy to prevent or treat gestational diabetes, *myo*-inositol showed a significant reduction in levels of fasting and postprandial blood glucose, HOMA-IR and insulinaemia.[19-21] We found a reduction in insulin and HOMA-IR in the intervention arm vs control, indicating a potentially increased insulin sensitivity with *myo*-inositol. Insulin levels and HOMA-IR appeared to reduce further with improved adherence. Our findings are in line with previous observations that *myo*-inositol reduces blood glucose levels by acting as an insulin sensitising agent. [16, 21] We did not observe any differences in the fasting or 2-hour blood glucose levels, but improved adherence showed the potential to reduce 2-hour glucose levels in the intervention group. It is likely that with improved adherence, any potential differences between groups will become clearer in any future studies.

Although there were no differences in the levels of c-peptide between the two arms, women who adhered to the intervention appeared to have a reduction in c-peptide levels, indicative of a reduction in insulin resistance. Participants in our study continued taking *myo*-inositol even after the diagnosis of gestational diabetes. We observed that fewer women in the intervention arm had large for gestational age (LGA) babies than in the control arm. The small sample size of this pilot trial means no statistical significance can be attributed to these estimates.[22] A future large scale trial will be needed to evaluate if the improvement in insulin sensitivity with *myo*-inositol contributed to the observed reduction in LGA babies.

For peer review only - http://bmjopen.bmj.com/site/about/guidelines.xhtml

Conclusions

The EMmY pilot trial is the first UK based multi-centre trial involving ethnically diverse high-risk women from inner city NHS hospitals in a trial of *myo*-inositol vs placebo. We have reported on acceptability, cost and quality outcomes, as well as the potential effect of *myo*-inositol on gestational diabetes. There is an indication of efficacy of *myo*-inositol in lowering insulin resistance, with the potential for preventing gestational diabetes. In order to tackle the observed barriers to adherence, we plan to use a tablet form of *myo*-inositol in a large-scale definitive trial, with an internal pilot first demonstrating the intended levels of adherence. We now need a large-scale definitive trial adequately powered to ascertain the effect of *myo*-inositol in a tablet form on gestational diabetes and adverse maternal and perinatal outcomes.

Authors' contributions

CEA- wrote the first and final version of the manuscript, collected and analysed the qualitative data.

ZD, DL, JDO, JH- coordinated the study, contributed to the methodology and the final version of the manuscript.

LS and AH- supervised the qualitative evaluation and has contributed to the final version of the manuscript.

EP- developed and conducted the economic evaluation. EP also contributed to the final version of the manuscript.

AT, JDa, SS, LP, AK, JM, GH, KSK and MSH- provided clinical input and contributed to the final version of the manuscript.

JZ, MDCP, TP- developed and lead the statistical evaluation and contributed to the final version of the manuscript.

FJGC- conducted the statistical analysis and contributed to the final version of the manuscript.

For peer review only - http://bmjopen.bmj.com/site/about/guidelines.xhtml

ST- designed and developed the trial, reviewed the manuscript and has contributed to the final version of the manuscript.

Acknowledgements

The authors acknowledge Pharmasure for the donation of *myo*-inositol and placebo tablets to the EMmY study and Montuno software for the discounted Dosecast app. The authors are grateful to the patient advisers and site midwives for their contributions towards the study.

The EMmY pilot trial was supported by the National Institute for Health Research (NIHR) Programme Grants for Applied Research. The views expressed are those of the authors and not necessarily those of the NHS, the NIHR or the Department of Health and Social Care.

KSK is a Distinguished Investigator funded by the Beatriz Galindo (Senior Modality) Program grant given to the University of Granada by the Ministry of Science, Innovation, and Universities of the Spanish Government.

Ethical approval

Ethical approval was obtained from the London Queen Square Research Ethics Committee (17/LO/1741).

Funding statement

The EMmY trial is sponsored by Queen Mary University of London and funded by Barts Charity, grant number MGU0373. The sponsor and the funder have no role in the study conduct, analysis and interpretation of the findings, and dissemination of the results. EP and AH are also supported by the NIHR Collaboration for Leadership in Applied Health Research at Barts Health NHS Foundation Trust (NIHR ARC North Thames). Professor Khalid S. Khan is Distinguished Investigator funded by the Beatriz Galindo (senior modality) grant to the University of Granada by the Spanish Ministry of Education.

For peer review only - http://bmjopen.bmj.com/site/about/guidelines.xhtml

For peer review only

Data Sharing Statement

Release of data will be subject to a data use agreement between the contractor and the third party requesting the data. The data use agreement must detail the agreed use and appropriate management of the research data to be shared and include a requirement for recognition of the contribution of the researchers who generated the data and the original funder.

Competing interests

None

For peer review only - http://bmjopen.bmj.com/site/about/guidelines.xhtml

References

1. Metzger BE, Lowe LP, Dyer AR, et al. Hyperglycemia and adverse pregnancy outcomes. *N Engl J Med*. May 2008;358(19):1991-2002. doi:10.1056/NEJMoa0707943
2. Bellamy L, Casas JP, Hingorani AD, Williams D. Type 2 diabetes mellitus after gestational diabetes: a systematic review and meta-analysis. *Lancet*. May 2009;373(9677):1773-9. doi:10.1016/S0140-6736(09)60731-5
3. Boney CM, Verma A, Tucker R, Vohr BR. Metabolic syndrome in childhood: association with birth weight, maternal obesity, and gestational diabetes mellitus. *Pediatrics*. Mar 2005;115(3):e290-6. doi:10.1542/peds.2004-1808
4. Rogozińska E, Chamillard M, Hitman GA, Khan KS, Thangaratinam S. Nutritional manipulation for the primary prevention of gestational diabetes mellitus: a meta-analysis of randomised studies. *PLoS One*. 2015;10(2):e0115526. doi:10.1371/journal.pone.0115526
5. Vitagliano A, Saccone G, Cosmi E, et al. Inositol for the prevention of gestational diabetes: a systematic review and meta-analysis of randomized controlled trials. *Arch Gynecol Obstet*. 01 2019;299(1):55-68. doi:10.1007/s00404-018-5005-0
6. Crawford TJ, Crowther CA, Alsweiler J, Brown J. Antenatal dietary supplementation with myo-inositol in women during pregnancy for preventing gestational diabetes. *Cochrane Database Syst Rev*. Dec 2015;(12):CD011507. doi:10.1002/14651858.CD011507.pub2
7. Eldridge SM, Chan CL, Campbell MJ, et al. CONSORT 2010 statement: extension to randomised pilot and feasibility trials. *Pilot Feasibility Stud*. 2016;2:64. doi:10.1186/s40814-016-0105-8
8. Amaefule CE, Drymoussi, Z., Dodds, J., Sweeney, L., Pizzo, E., Daru, J., Robson, J., Poston, L., Khalil, A., Myers, J. and Harden, A.,. Effectiveness and acceptability of myo-inositol nutritional supplement in the prevention of gestational diabetes (EMmY): a protocol for a randomised, placebo-controlled, double-blind pilot trial. *BMJ open*. 2018;8(9):p.e022831.
9. National Institute for Health and Care Excellence. Diabetes in pregnancy: management from pre-conception to the post-natal period. Accessed 26th November, 2019. https://www.nice.org.uk/guidance/ng3/chapter/1-Recommendations#gestational-diabetes-2
10. Braun, V. and Clarke, V. Using thematic analysis in psychology. Qualitative research in psychology. *Qualitative Research in Psychology*. 2006;*3* (2):pp.77-101.
11. Amaefule CE, Sweeney, L., Harden, A., Thangaratinam, S.,. Pilot trial on myo-inositol supplementation in pregnancy: A qualitative study with women and research staff on the feasibility and acceptability of the trial and intervention. *To be published at BMJ open*. 2020;
12. Moss N, Daru J, Lanz D, Thangaratinam S, Khan KS. Involving pregnant women, mothers and members of the public to improve the quality of women's health research. *BJOG*. Feb 2017;124(3):362-365. doi:10.1111/1471-0528.14419
13. Husain S, Allotey J, Drymoussi Z, et al. Effects of oral probiotic supplements on vaginal microbiota during pregnancy: a randomised, double-blind, placebo-controlled trial with microbiome analysis. *BJOG*. 01 2020;127(2):275-284. doi:10.1111/1471-0528.15675
14. Poston L, Bell R, Croker H, et al. Effect of a behavioural intervention in obese pregnant women (the UPBEAT study): a multicentre, randomised controlled trial. *Lancet Diabetes Endocrinol*. Oct 2015;3(10):767-77. doi:10.1016/S2213-8587(15)00227-2
15. D'Anna R, Scilipoti A, Giordano D, et al. myo-Inositol supplementation and onset of gestational diabetes mellitus in pregnant women with a family history of type 2 diabetes: a prospective, randomized, placebo-controlled study. *Diabetes Care*. Apr 2013;36(4):854-7. doi:10.2337/dc12-1371
16. D'Anna R, Di Benedetto A, Scilipoti A, et al. Myo-inositol Supplementation for Prevention of Gestational Diabetes in Obese Pregnant Women: A Randomized Controlled Trial. *Obstet Gynecol*. Aug 2015;126(2):310-5. doi:10.1097/AOG.0000000000000958
17. Laughland AaK, L.,. Should participants be left to their own devices? Comparing paper and smartphone diaries in psychological research. *Journal of applied research in memory and cognition*. 2018;7(4):pp.552-563.

For peer review only - http://bmjopen.bmj.com/site/about/guidelines.xhtml

18. Brennan M, Clarke M, Devane D, Dowling M. A qualitative study of the factors influencing recruitment to a pilot trial on the prevention of striae gravidarum. *BMC Pregnancy Childbirth*. Feb 2020;20(1):103. doi:10.1186/s12884-020-2781-x

19. Fraticelli F, Celentano C, Zecca IA, et al. Effect of inositol stereoisomers at different dosages in gestational diabetes: an open-label, parallel, randomized controlled trial. *Acta Diabetol*. Aug 2018;55(8):805-812. doi:10.1007/s00592-018-1157-4

20. Corrado F, D'Anna R, Di Vieste G, et al. The effect of myoinositol supplementation on insulin resistance in patients with gestational diabetes. *Diabet Med*. Aug 2011;28(8):972-5. doi:10.1111/j.1464-5491.2011.03284.x

21. Miñambres I, Cuixart G, Gonçalves A, Corcoy R. Effects of inositol on glucose homeostasis: Systematic review and meta-analysis of randomized controlled trials. *Clin Nutr*. 06 2019;38(3):1146-1152. doi:10.1016/j.clnu.2018.06.957

22. Arnold DM, Burns KE, Adhikari NK, et al. The design and interpretation of pilot trials in clinical research in critical care. *Crit Care Med*. Jan 2009;37(1 Suppl):S69-74. doi:10.1097/CCM.0b013e3181920e33

Figure Legends, Tables and Appendices

Figure 1 CONSORT diagram for the EMmY study

Figure 2 Rate of recruitment and randomisation in all participating sites in the EMmY trial

Table 1 Baseline characteristics of EMmY participants randomised to *myo*-inositol vs placebo

Table 2 Glycaemic outcomes in the intervention and control arms of the EMmY trial

Table 3 Maternal and perinatal clinical outcomes in the EMmY study

Appendix 1 Clinical maternal and perinatal outcomes assessed within the EMmY study

Appendix 2 Criteria for progression to a full-scale trial

Appendix 3 Characteristics of the 15 women interviewed using pseudonyms

Appendix 4 Proportion of screened, recruited and randomised women in each participating site

Appendix 5 Costs and QALYS in both intervention and control groups

Appendix 6 Impact of adherence at 28 weeks on key clinical and laboratory outcomes

Appendix 7 Completeness of clinical outcome data

For peer review only

For peer review only - http://bmjopen.bmj.com/site/about/guidelines.xhtml

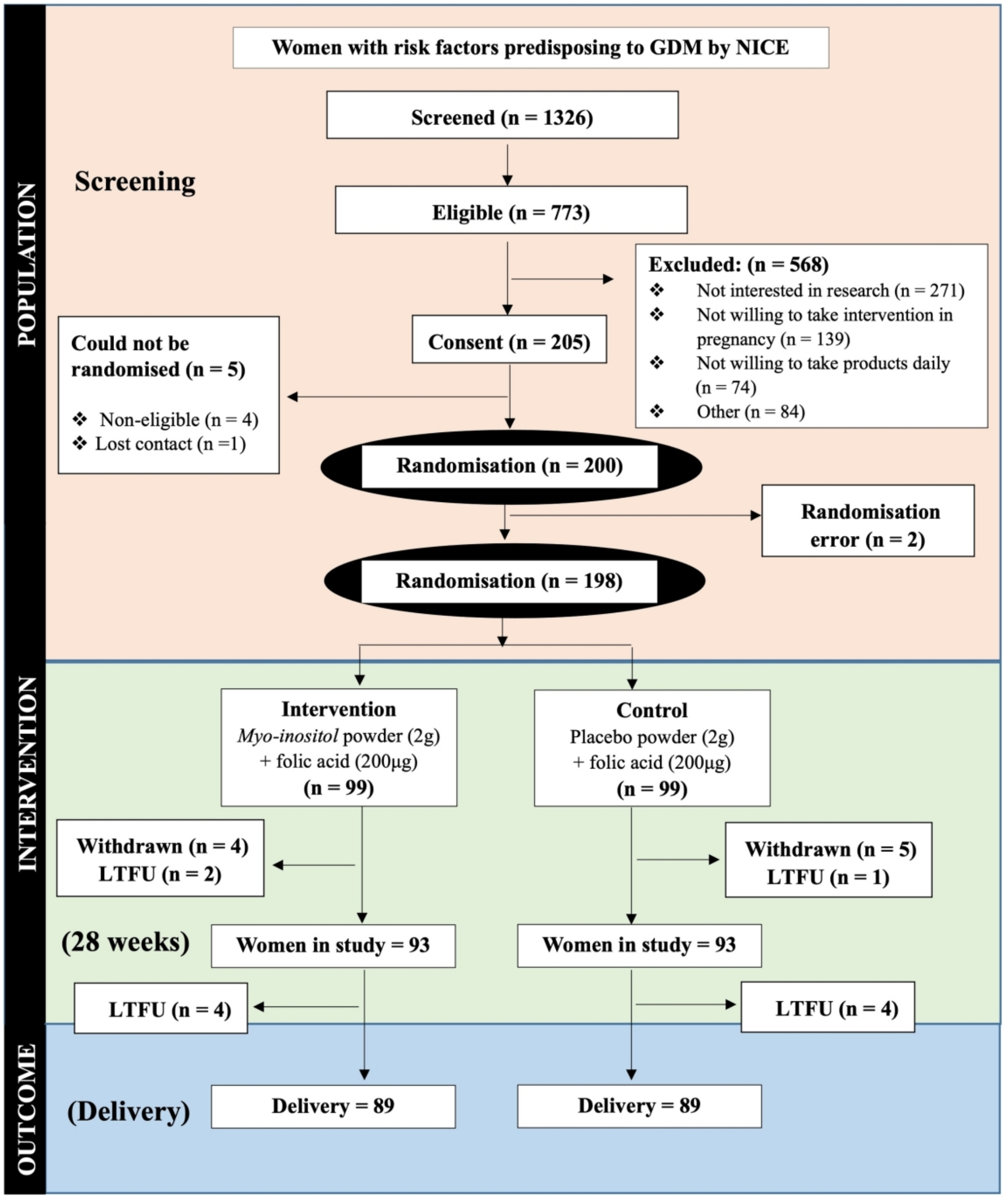

Figure 1. Flow chart of participants in the EMmY study

196x233mm (300 x 300 DPI)

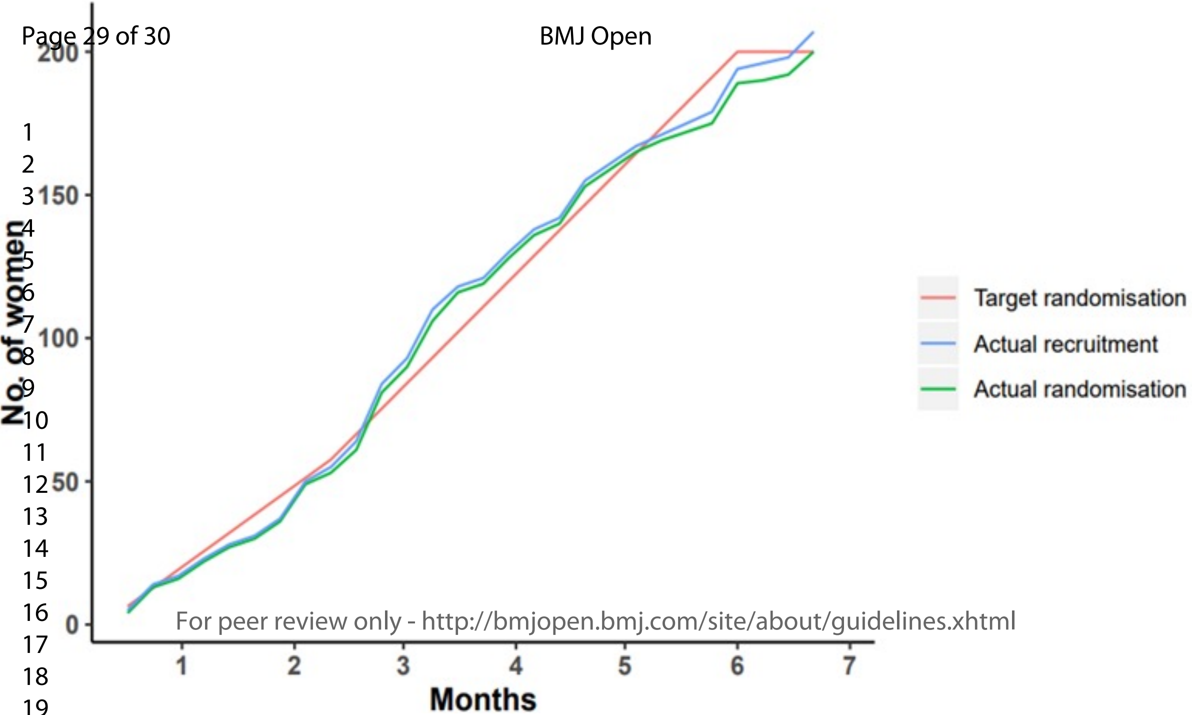

For peer review only - http://bmjopen.bmj.com/site/about/guidelines.xhtml

CONSORT 2010 checklist of information to include when reporting a pilot or feasibility trial*

Section/Topic	Item No	Checklist item	Reported on page No
**Title and abstract**			
	1a	Identification as a pilot or feasibility randomised trial in the title	1
	1b	Structured summary of pilot trial design, methods, results, and conclusions (for specific guidance see CONSORT abstract extension for pilot trials)	3
**Introduction**			
Background and objectives	2a	Scientific background and explanation of rationale for future definitive trial, and reasons for randomised pilot trial	5
	2b	Specific objectives or research questions for pilot trial	5
**Methods**			
Trial design	3a	Description of pilot trial design (such as parallel, factorial) including allocation ratio	6
	3b	Important changes to methods after pilot trial commencement (such as eligibility criteria), with reasons	n/a
Participants	4a	Eligibility criteria for participants	6
	4b	Settings and locations where the data were collected	6
	4c	How participants were identified and consented	7-8
Interventions	5	The interventions for each group with sufficient details to allow replication, including how and when they were actually administered	6
Outcomes	6a	Completely defined prespecified assessments or measurements to address each pilot trial objective specified in 2b, including how and when they were assessed	6-7
	6b	Any changes to pilot trial assessments or measurements after the pilot trial commenced, with reasons	n/a
	6c	If applicable, prespecified criteria used to judge whether, or how, to proceed with future definitive trial	8
Sample size	7a	Rationale for numbers in the pilot trial	7
	7b	When applicable, explanation of any interim analyses and stopping guidelines	n/a
Randomisation:			
Sequence generation	8a	Method used to generate the random allocation sequence	7
	8b	Type of randomisation(s); details of any restriction (such as blocking and block size)	7-8
Allocation concealment mechanism	9	Mechanism used to implement the random allocation sequence (such as sequentially numbered containers), describing any steps taken to conceal the sequence until interventions were assigned	7-8

For peer review only

Implementation	10	Who generated the random allocation sequence, who enrolled participants, and who assigned participants to interventions	7-8
Blinding	11a	If done, who was blinded after assignment to interventions (for example, participants, care providers, those assessing outcomes) and how	8
	11b	If relevant, description of the similarity of interventions	6
Statistical methods	12	Methods used to address each pilot trial objective whether qualitative or quantitative	8-9
**Results**			
Participant flow (a diagram is strongly recommended)	13a	For each group, the numbers of participants who were approached and/or assessed for eligibility, randomly assigned, received intended treatment, and were assessed for each objective	10
	13b	For each group, losses and exclusions after randomisation, together with reasons	10
Recruitment	14a	Dates defining the periods of recruitment and follow-up	10
	14b	Why the pilot trial ended or was stopped	n/a
Baseline data	15	A table showing baseline demographic and clinical characteristics for each group	10
Numbers analysed	16	For each objective, number of participants (denominator) included in each analysis. If relevant, these numbers should be by randomised group	10-11
Outcomes and estimation	17	For each objective, results including expressions of uncertainty (such as 95% confidence interval) for any estimates. If relevant, these results should be by randomised group	10-11
Ancillary analyses	18	Results of any other analyses performed that could be used to inform the future definitive trial	12-15
Harms	19	All important harms or unintended effects in each group (for specific guidance see CONSORT for harms)	11
	19a	If relevant, other important unintended consequences	n/a
**Discussion**			
Limitations	20	Pilot trial limitations, addressing sources of potential bias and remaining uncertainty about feasibility	15-16
Generalisability	21	Generalisability (applicability) of pilot trial methods and findings to future definitive trial and other studies	17
Interpretation	22	Interpretation consistent with pilot trial objectives and findings, balancing potential benefits and harms, and considering other relevant evidence	16-17
	22a	Implications for progression from pilot to future definitive trial, including any proposed amendments	17-18
**Other information**			
Registration	23	Registration number for pilot trial and name of trial registry	3
Protocol	24	Where the pilot trial protocol can be accessed, if available	n/a
Funding	25	Sources of funding and other support (such as supply of drugs), role of funders	21
	26	Ethical approval or approval by research review committee, confirmed with reference number	21

For peer review only

Citation: Eldridge SM, Chan CL, Campbell MJ, Bond CM, Hopewell S, Thabane L, et al. CONSORT 2010 statement: extension to randomised pilot and feasibility trials. BMJ. 2016;355.
*We strongly recommend reading this statement in conjunction with the CONSORT 2010, extension to randomised pilot and feasibility trials, Explanation and Elaboration for important clarifications on all the items. If relevant, we also recommend reading CONSORT extensions for cluster randomised trials, non-inferiority and equivalence trials, non-pharmacological treatments, herbal interventions, and pragmatic trials. Additional extensions are forthcoming: for those and for up to date references relevant to this checklist, see www.consort-statement.org.

For peer review only

BMJ Open

Myo-inositol nutritional supplement for prevention of gestational diabetes (EMmY): a randomised, placebo-controlled, double-blind pilot trial with nested qualitative study

Journal:	*BMJ Open*
Manuscript ID	bmjopen-2021-050110.R2
Article Type:	Original research
Date Submitted by the Author:	22-Jan-2022
Complete List of Authors:	Amaefule, Chiamaka; Queen Mary University of London Barts and The London School of Medicine and Dentistry, BARC (Barts Research Centre for Women’s Health), Institute of Population Health Sciences<br>Drymoussi, Zoe; Queen Mary University of London Barts and The London School of Medicine and Dentistry, (BARC) Barts Research Centre for Women’s Health, Institute for Population Health Sciences<br>Gonzalez Carreras , Francisco; Queen Mary University of London Barts and The London School of Medicine and Dentistry, BARC (Barts Research Centre for Women’s Health), Institute of Population Health Sciences<br>Pardo Llorente , Maria del Carmen; Complutense University of Madrid, Department of Statistics and Operational Research<br>Lanz, Doris; Queen Mary University of London Barts and The London School of Medicine and Dentistry, BARC (Barts Research Centre for Women’s Health), Institute of Population Health Sciences<br>Dodds, Julie; Queen Mary University of London Barts and The London School of Medicine and Dentistry, BARC (Barts Research Centre for Women’s Health), Institute of Population Health Sciences<br>Sweeney, Lorna; University of East London, Institute for Health and Human Development<br>Pizzo, Elena; University College London, NIHR ARC (Applied Health Collaborations) for North Thames London, Department of Applied Health Research<br>Thomas, Amy; Queen Mary University of London Barts and The London School of Medicine and Dentistry, BARC (Barts Research Centre for Women’s Health), Institute of Population Health Sciences; The Royal London Hospital, Women’s and Children’s Health, Barts Health NHS Trust<br>Heighway, James; Queen Mary University of London Barts and The London School of Medicine and Dentistry, BARC (Barts Research Centre for Women’s Health), BARC (Barts Research Centre for Women’s Health), Institute of Population Health Sciences<br>Daru, Jahnavi; Queen Mary University of London Barts and The London School of Medicine and Dentistry, (BARC) Barts Research Centre for Women’s Health, Institute for Population Health Sciences<br>Sobhy, Soha ; Queen Mary University of London Barts and The London School of Medicine and Dentistry, (BARC) Barts Research Centre for Women’s Health, Institute for Population Health Sciences; The Royal London Hospital, Women’s and Children’s Health, Barts Health NHS Trust<br>Poston, Lucilla; King's College London, Department of Women and

	Children’s Health, Faculty of Life Sciences and Medicine<br>Khalil, Asma; St George's Hospital, Department of Obstetrics and Gynaecology; St George's University of London, Vascular Biology Research Centre, Molecular and Clinical Sciences Research Institute<br>Myers, Jenny; The University of Manchester, Maternal and Fetal Health Research Centre, Manchester Academic Health Science Centre<br>Harden, Angela; University of East London, Institute for Health and Human Development<br>Hitman, Graham; Queen Mary University of London Barts and The London School of Medicine and Dentistry, Centre for Genomics and Child Health, Blizard Institute<br>Khan , Khalid; Universidad de Granada, Department of Preventive Medicine and Public Health<br>Zamora, Javier; IRYCIS, Clinical Biostatistics Unit and CIBER Epidemiology and Public Health; University of Birmingham, WHO Collaborating Centre for Global Women’s Health, Institute of Metabolism and Systems Research<br>Pérez, Teresa ; Queen Mary University of London Barts and The London School of Medicine and Dentistry, BARC (Barts Research Centre for Women’s Health), Institute of Population Health Sciences; Complutense University of Madrid, Department of Statistics and Data Science<br>Huda, Mohammed SB; The Royal London Hospital Diabetes Diabetes and Metabolism, Barts Health NHS Trust<br>Thangaratinam, Shakila; University of Birmingham, WHO Collaborating Centre for Global Women’s Health, Institute of Metabolism and Systems Research; Birmingham Women's and Children's NHS Foundation Trust
**Primary Subject Heading**:	Obstetrics and gynaecology
Secondary Subject Heading:	Qualitative research, Diabetes and endocrinology
Keywords:	Diabetes in pregnancy < DIABETES & ENDOCRINOLOGY, HEALTH ECONOMICS, Maternal medicine < OBSTETRICS, QUALITATIVE RESEARCH, PUBLIC HEALTH

SCHOLARONE™
Manuscripts

I, the Submitting Author has the right to grant and does grant on behalf of all authors of the Work (as defined in the below author licence), an exclusive licence and/or a non-exclusive licence for contributions from authors who are: i) UK Crown employees; ii) where BMJ has agreed a CC-BY licence shall apply, and/or iii) in accordance with the terms applicable for US Federal Government officers or employees acting as part of their official duties; on a worldwide, perpetual, irrevocable, royalty-free basis to BMJ Publishing Group Ltd ("BMJ") its licensees and where the relevant Journal is co-owned by BMJ to the co-owners of the Journal, to publish the Work in this journal and any other BMJ products and to exploit all rights, as set out in our licence.

The Submitting Author accepts and understands that any supply made under these terms is made by BMJ to the Submitting Author unless you are acting as an employee on behalf of your employer or a postgraduate student of an affiliated institution which is paying any applicable article publishing charge ("APC") for Open Access articles. Where the Submitting Author wishes to make the Work available on an Open Access basis (and intends to pay the relevant APC), the terms of reuse of such Open Access shall be governed by a Creative Commons licence – details of these licences and which Creative Commons licence will apply to this Work are set out in our licence referred to above.

Other than as permitted in any relevant BMJ Author's Self Archiving Policies, I confirm this Work has not been accepted for publication elsewhere, is not being considered for publication elsewhere and does not duplicate material already published. I confirm all authors consent to publication of this Work and authorise the granting of this licence.

For peer review only

Myo-inositol nutritional supplement for prevention of gestational diabetes (EMmY): a randomised, placebo-controlled, double-blind pilot trial with nested qualitative study

Chiamaka Esther Amaefule[1] Email: c.e.amaefule@qmul.ac.uk; Zoe Drymoussi[1] Email: zdrymoussi@gmail.com; Francisco Jose Gonzalez Carreras [1] Email: f.j.gonzalez.carreras@gmail.com; Maria del Carmen Pardo Llorente [1,2] Email: mcapardo@mat.ucm.es; Doris Lanz[1] Email: d_lanz@hotmail.com; Julie Dodds[1] Email: jpdodds247@gmail.com; Lorna Sweeney[3] Email: lorna.christine@gmail.com; Elena Pizzo[4] Email: e.pizzo@ucl.ac.uk; Amy Thomas[1,5] Email: amy.thomas28@nhs.net; James Heighway[1] Email: j.heighway@qmul.ac.uk; Jahnavi Daru[1] Email: j.daru@qmul.ac.uk; Soha Sobhy[1,5] Email: soha.sobhy@qmul.ac.uk; Lucilla Poston[6] Email: lucilla.poston@kcl.ac.uk; Asma Khalil[7,8] Email: akhalil@sgul.ac.uk; Jenny Myers[9] Email: Jenny.Myers@manchester.ac.uk; Angela Harden[3] Email: a.harden@uel.ac.uk; Graham A Hitman[10] Email: g.a.hitman@qmul.ac.uk; Khalid Saeed Khan[11] Email: profkkhan@gmail.com; Javier Zamora [12, 13] Email: javier.zamora@hrc.es; Teresa Pérez [1,14] Email: teperez@estad.ucm.es; Mohammed SB Huda[15] Email: bobby.huda1@nhs.net; Shakila Thangaratinam [13,16]* Email: s.thangaratinam.1@bham.ac.uk

* Corresponding author

[1] BARC (Barts Research Centre for Women's Health), Institute of Population Health Sciences, Barts and the London School of Medicine and Dentistry, Queen Mary University of London, London, UK

[2] Department of Statistics and Operational Research, Complutense University of Madrid, Madrid Spain.

[3]Institute for Health and Human Development, University of East London, London, UK

[4] NIHR ARC (Applied Research Collaborations) for North Thames London, Department of Applied Health Research, University College London, London, UK.

For peer review only

[5]Women's and Children's Health, Royal London Hospital, Barts Health NHS Trust, London UK.

[6] Department of Women and Children's Health, Faculty of Life Sciences and Medicine, King's College London, London UK.

[7] Department of Obstetrics and Gynaecology, St George's Hospital, London UK.

[8] Vascular Biology Research Centre, Molecular and Clinical Sciences Research Institute, St George's University of London, Cranmer Terrace, London UK.

[9] Maternal and Fetal Health Research Centre, University of Manchester, Manchester Academic Health Science Centre, UK.

[10] Centre for Genomics and Child Health, Blizard Institute, Barts and the London School of Medicine and Dentistry, Queen Mary University of London, London, UK.

[11] Department of Preventive Medicine and Public Health, Universidad de Granada, Granada, Spain.

[12] Clinical Biostatistics Unit (IRYCIS) and CIBER Epidemiology and Public Health, Madrid, Spain.

[13] WHO Collaborating Centre for Global Women's Health, Institute of Metabolism and Systems Research, University of Birmingham, Birmingham, UK.

[14] Department of Statistics and Data Science, Complutense University of Madrid, Madrid, Spain.

[15] Department of Diabetes & Metabolism, Barts Health NHS Trust, Royal London Hospital, London, UK.

[16] Birmingham Women's and Children's NHS Foundation Trust, Birmingham, UK.

WORD COUNT: 4076

For peer review only

For peer review only - http://bmjopen.bmj.com/site/about/guidelines.xhtml

ABSTRACT

Objectives: To determine the feasibility and acceptability of conducting a randomised trial on the effects of *myo*-inositol in preventing gestational diabetes in high-risk pregnant women.

Design: A multicentre, double-blind, placebo-controlled, pilot randomised trial with nested qualitative evaluation.

Setting: Five inner city UK National Health Service (NHS) hospitals

Participants: Multi-ethnic pregnant women at 12^{+0} and 15^{+6} weeks' gestation with risk factors for gestational diabetes.

Interventions: 2g of *myo*-inositol or placebo, both included 200µg folic acid, twice daily until delivery.

Primary outcome measures: Rates of recruitment, randomisation, adherence and follow-up.

Secondary outcome measures: Glycaemic indices (including homeostatic model assessment-insulin resistance HOMA-IR), gestational diabetes (diagnosed using oral glucose tolerance test at 28 weeks and by delivery), maternal, perinatal outcomes, acceptability of intervention, and costs.

Results: Of the 1326 women screened, 58% (773/1326) were potentially eligible, and 27% (205/773) were recruited. We randomised 97% (198/205) of all recruited women (99 each in intervention and placebo arms), and ascertained outcomes in 90% of women (178/198) by delivery. The mean adherence was 52% (SD 44) at 28weeks' and 34% (SD 41) at 36 weeks' gestation. HOMA-IR and serum insulin levels were lower in the myo-inositol vs placebo arm (Mean Difference -0.6, 95% CI -1.2 to 0.0 and -2.69, 95% CI -5.26 to -0.18 respectively). The study procedures were acceptable to women and healthcare professionals. Women who perceived themselves at high risk of gestational diabetes were more likely to participate and adhere to the intervention. The powder form of *myo*-inositol and placebo, along with nausea in pregnancy were key barriers to adherence.

For peer review only

Conclusions: A future trial on *myo*-inositol vs placebo to prevent gestational diabetes is feasible. The intervention will need to be delivered in a non-powder form to improve adherence. There is a signal for efficacy in reducing insulin resistance in pregnancy with *myo*-inositol.

Trial registration: ISRCTN48872100

Funding: Funded by Barts Charity, grant number MGU0373

Key words: diabetes in pregnancy, maternal medicine, qualitative research, public health, health economics

Abbreviations: CONSORT (Consolidated Standards of Reporting Trials); EQ-5D-5L (European Quality of life 5-Dimensions 5-Level scale); gestational diabetes; IADPSG (International Association of Diabetes and Pregnancy Study Groups); LGA (Large for Gestational Age); LTFU (Lost To Follow-Up); NHS (National Health Service); NICE (National Institute for Health and Care Excellence); NIHR (National Institute for Health Research); QALY (Quality-Adjusted Life-Year)

STRENGTHS AND LIMITATIONS

- Clinically relevant multi-centre trial involving ethnically diverse high-risk women from inner city NHS hospitals in the UK, looking at *myo*-inositol vs placebo during pregnancy.
- This pilot study, with a qualitative component, is based on a prospective protocol aimed at informing the feasibility of a full scale RCT on the effect of *myo*-inositol vs placebo in preventing gestational diabetes in high-risk women.
- Process outcomes relevant to recruitment, adherence and retention have been assessed in detail.
- Self-reported adherence rather than objective methods (e.g. pill-counting) were used.

For peer review only - http://bmjopen.bmj.com/site/about/guidelines.xhtml

For peer review only

99 • The study allowed only limited evaluation of clinical outcomes due to the small sample size.

For peer review only

For peer review only - http://bmjopen.bmj.com/site/about/guidelines.xhtml

INTRODUCTION

Primary prevention of gestational diabetes, a condition with high glucose levels first identified in pregnancy, is key to minimising maternal and perinatal complications associated with the condition.[1] Prevention of gestational diabetes can also reduce the risk of type 2 diabetes in mothers and their children in the long term. [2,3] Simple, easy to administer, and acceptable interventions are needed to prevent gestational diabetes.

Myo-inositol, a nutritional supplement that is present in fruits and fibre rich food and available as over-the-counter nutritional supplement, has the potential to prevent gestational diabetes through its insulin sensitising action. Few small trials have shown a promising role for *myo*-inositol with reductions in rates of gestational diabetes by up to 60%.[4, 5] But, in addition to small sample sizes (< 300 women), these trials were from a single country (Italy) and involved only Caucasian women.[5] The generalisability of these findings to high risk multi-ethnic populations who are most at risk of gestational diabetes is not known. A Cochrane review has called for further large trials on the effects of *myo*-inositol on gestational diabetes and complications in multi-ethnic populations. We also need to determine the costs and health service use associated with the intervention.[6]

Prior to undertaking a full-scale definitive trial on the effect of *myo*-inositol in preventing gestational diabetes, which requires substantial resources, we need to assess the feasibility of conducting such a trial beforehand. We undertook a pilot randomised trial comparing *myo*-inositol vs placebo, with a nested qualitative study, to assess the feasibility of the trial design, explore the acceptability and contextual issues around intervention delivery and trial procedures, and the potential effects on glycaemic, maternal, perinatal, cost, and quality of life outcomes.

For peer review only - http://bmjopen.bmj.com/site/about/guidelines.xhtml

METHODS

The EMmY trial was a multicentre, randomised, placebo-controlled, double-blind pilot trial with nested qualitative study and economic evaluation conducted with a prospective protocol and reported using CONSORT (Consolidated Standards of Reporting Trials) guidelines.[7] The full details of the study protocol are published elsewhere.[8]

Participants and setting

We screened pregnant women attending five inner city maternity units in London and Manchester. Women were eligible for recruitment if they were at least 16 years of age with a viable singleton pregnancy between 12^{+0} and 15^{+6} weeks' gestation, were able to provide written informed consent in English, and were considered to be at high risk of gestational diabetes as per the NICE (National Institute for health and Care Excellence) criteria.[9] We excluded women with type 2 diabetes, and those taking metformin or corticosteroids. Recruited women with previous history of gestational diabetes underwent additional testing with OGTT (oral glucose tolerance test) or HbA1c, and were randomised after ruling out undiagnosed type 2 diabetes (OGTT fasting ≥5.6 mmol/l, 2hour glucose ≥7.8 mol/l or HbA1c ≥48 mmol/mol).

Intervention and control group allocation

The intervention group were provided with *myo*-inositol 2g supplement with 200μg folic acid, to be taken twice a day in a powder form mixed in water, approximately 1 hour before or after a meal. The placebo was an identical looking and tasting powder of Xylitol filler with 200μg folic acid to be taken twice a day.

Outcomes

The primary outcomes were the proportion of screened women who were eligible, the proportion of eligible women who were recruited and randomised, rates of adherence and follow-up. The

For peer review only - http://bmjopen.bmj.com/site/about/guidelines.xhtml

secondary outcomes were acceptability of the intervention and trial to women and health professionals; laboratory outcomes related to glycaemia such as fasting and 2-hour plasma glucose levels in 75g OGTT, HOMA-IR, serum insulin, adiponectin, leptin and urinary inositol at 28 weeks' gestation, and maternal and cord blood c-peptide at delivery. Other secondary outcomes included diagnosis of gestational diabetes at 28 weeks' gestation by modified IADPSG (International Association of Diabetes and Pregnancy Study Groups) and NICE criteria, maternal and perinatal morbidity and mortality (Appendix 1). The economic outcomes included costs (NHS health and social care resources use) and quality of life. Other process outcomes included deviations from study protocol, completeness of data collection and the level of support required by the site team for trial delivery.

For peer review only

Sample size

We expected that 1500 women would be booked for antenatal care each month at the participating hospitals, and at least 300 of those would be eligible. Assuming 1000 eligible women were approached, we expected 25% (250/1000) to be consented. We expected that 20% (50) of these would be women with a previous history of gestational diabetes, with abnormal HbA1C and/ or OGTT and would be excluded. This would result in 200 women randomised.

Study conduct

We approached pregnant women at booking and screened them for eligibility. After obtaining written informed consent from eligible women, we collected baseline information on demographic and clinical characteristics, and women completed the validated European Quality of life 5-Dimensions 5-Level scale (EQ-5D-5L) questionnaire on quality of life measures. Women were randomised between 12^{+0} and 15^{+6} weeks' gestation using an online randomisation system (administered by epiGenesys, University of Sheffield) to either the *myo*-inositol or placebo treatment arm. We used a randomisation scheme based on permuted blocks of random

For peer review only - http://bmjopen.bmj.com/site/about/guidelines.xhtml

block size (sizes 4, 6 and 8) stratified by participating site. No adaptive or minimisation strategies were used. Participants, healthcare providers and researchers were blinded to the group allocation. We followed-up women in person or by phone at 20, 28- and 36-weeks' gestation and at delivery. All women were offered OGTT at 28 weeks' gestation to screen for gestational diabetes. Women self-reported adherence to the intervention using a paper-based diary, or an optional mobile application. Where possible, we supplemented this information with a count of unused sachets. The trial was overseen monthly by a Trial Management Group and biannually by the Project Steering Committee (combining functions from a more traditional set up of a separate Data Monitoring Committee and Trial Steering Committee) reviewing trial progress and conduct. We prespecified the criteria for progression to a full-scale trial (Appendix 2).

Analysis

Using an intention-to-treat (ITT) approach, we summarised the feasibility outcomes using proportions with 95% CI (confidence interval) and other descriptive statistics. For continuous laboratory outcomes, we calculated the effect sizes (e.g. mean differences) with 95% CI. We reported the rates of dichotomous clinical outcomes and costs in the two arms of the study. The impact of a previous history of gestational diabetes and the use of the mobile app on adherence were conducted as a post-hoc exploratory analysis. All analyses were performed using R, version 3.6.1.

Qualitative study

We explored the experiences and perspectives of women and healthcare professionals on the intervention and trial procedures, using observations and interview methods. Recruitment appointments were observed to ascertain women's specific motivations or concerns regarding participating in the trial, as well as contextual factors that may optimise or discourage participation. We invited eligible women who declined trial participation to give their reasons

For peer review only - http://bmjopen.bmj.com/site/about/guidelines.xhtml

verbally to the researcher or complete an open-ended questionnaire for reasons for declining consent. We interviewed women and healthcare professionals involved in trial delivery to explore the barriers and facilitators of participation, adherence and retention in the study as well as trial delivery in interviews with health professionals. Interview data was transcribed and analysed using a thematic analytical approach.[10, 11] Purposive sampling was used to ensure the women interviewed were from diverse age groups, ethnic backgrounds, clinical characteristics and levels of adherence (Appendix 3). We contacted women who withdrew from the trial, and midwives to discuss in depth the reasons for withdrawal.

Economic evaluation

A cost-utility analysis of *myo*-inositol supplementation versus placebo "within trial" period was undertaken. The cost utility measures were the incremental cost per unit of change per Quality-Adjusted Life-Year (QALY) gained. Cost was assessed from the perspective of the NHS and personal social services. The unit costs were retrieved from published sources, calculated in pound sterling based on 2018 costs. Resource use data was collected retrospectively. For each participant, their EQ-5D-5L health state was converted to a single summary index (utility value), constructing a utility profile based on the assumption of a straight-line relation between their utility values and each measurement point. For each participant, QALYs from baseline to 28 weeks were calculated as the area under the utility profile.

Patient and Public Involvement (PPI)

We sought input from Katie's Team, a women's health and childbirth PPI advisory group[12], who contributed to the study design and patient-facing documents for the pilot trial, and the interview schedule for the qualitative study.[8] Patients and members of the public were not involved in the recruitment to and conduct of the study. We plan to disseminate results from the EMmY study to

For peer review only - http://bmjopen.bmj.com/site/about/guidelines.xhtml

participants and members of Katie's Team, who can then circulate further through their networks and social media platforms.

RESULTS

Over six months (Feb - Sep 2018), we screened 1326 women across all five participating sites, and randomised 198 women to either intervention or control arm (Figure 1). The majority of participants in both arms were of ethnic minority origin (73% intervention; 60% control), over a third were obese (37% intervention; 36% control), and around half (51% intervention; 40% control) were nulliparous. About 60% of all women had a first degree relative with type 2 diabetes, and one in ten (8% intervention; 11% control) had a history of previous gestational diabetes (Table 1). Urinary inositol levels were higher in the *myo*-inositol than the placebo arm (MD 70.76 mg/L, 95% CI 11.2 to 130.8) (Table 2).

Table 1 Baseline characteristics of EMmY participants randomised to *myo*-inositol vs placebo

Baseline characteristics	Intervention (*Myo*-inositol) N=99 n (%) or Mean(SD)	Control (Placebo) N=99 N (%) or Mean(SD)
**Demography**		
Age (years)	31.4 (5.8)	31.9 (5.6)
Gestational age at recruitment (weeks)	12.4 (1.3)	12.3 (1.5)
BMI (kg/m^2)	28.2 (6.4)	27.9 (5.6)
Higher education	73 (73.7%)	76 (76.8%)
**Ethnicity**		
White	25 (25.3%)	40 (40.4%)
Asian	49 (49.5%)	48 (48.5%)
Black	16 (16.2%)	8 (8.1%)
Mixed/others	9 (9.1%)	3 (3%)
**Risk factors for GDM**		
GDM in previous pregnancy	8 (8.1%)	11 (11.1%)
Obesity (BMI ≥30 kg/m^2)	37 (37.4%)	36 (36.4%)
Minority Ethnic origin	72 (72.7%)	59 (59.6%)
Polycystic Ovary Syndrome	9 (9.1%)	11 (11.1%)
Previous macrosomic baby (>4.5kg)	4 (4%)	2 (2%)
Family history of diabetes (1st degree)	60 (60.6%)	59 (59.6%)
**Current pregnancy**		
Parity	0.8 (1)	0.9 (0.9)
Nulliparous	50 (50.5%)	40 (40.4%)
Alcohol use	1 (1%)	0 (0%)
Tobacco use	1 (1%)	3 (3%)
Current use of supplements	85 (85.9%)	89 (89.9%)
Current use of medication	24 (24.2%)	18 (18.2%)
Aspirin	8 (8.1%)	7 (7.1%)
**General clinical history**		

For peer review only - http://bmjopen.bmj.com/site/about/guidelines.xhtml

*Pre-existing medical condition	4 (4%)	4 (4%)
Family history of raised lipids (1st degree)	7 (7.1%)	8 (8.1%)

*GDM- Gestational Diabetes Mellitus, BMI- Body Mass Index; *Pre-existing medical conditions include autoimmune disease, blood/clotting disorder, cardiac problems, liver disease, lung disease, chronic hypertension, and renal disease.*

Table 2 Glycaemia related outcomes in the intervention and control arms of the EMmY trial

**Outcomes (unit) (intervention;control)***	**Intervention (*myo*-inositol) Mean (SD)**	**Control (placebo) Mean (SD)**	**MD (95% CI)**
OGTT fasting (mmol/l) (81;81)	4.5 (0.5)	4.6 (0.4)	-0.0 (-0.2;0.1)
OGTT 2 hours (mmol/l) (80;81)	6.1 (2)	6.0 (1.3)	0.0 (-0.5;0.6)
HOMA-IR (50;62)	2.13 (1.32)	2.8 (2.05)	**-0.6 (-1.2;0.0)**
Insulin (mlU/L) (53;63)	10.63 (6.11)	13.72 (8.89)	**-2.69 (-5.26; -0.18)**
Leptin (µg/L) (53;63)	42.7 (21.6)	38.3 (17.1)	4.4 (-2.7;11.4)
Adiponectin (mg/L) (53;63)	6.6 (3.5)	6.1 (3.8)	0.5 (-0.9;1.8)
Urinary inositol (mg/L) (53;65)	265.7 (209.7)	194.7 (118.7)	70.76 (11.2;130.8)
c-peptide (µg/L) (53;63)	1538.5 (679.6)	1804.3 (807.8)	-253.6 (-522.7;10.4)
c-peptide in cord blood (µg/L) (10;6)	457 (268.04)	535 (296.02)	-101.74 (-358.17; 202.17)

**Number of women in the intervention and control group respectively; MD – Mean Difference*

PRIMARY OUTCOMES

Of the 773 eligible women, 27% (205/773) consented to be involved in the study. Of these, 98% (198/205) were randomised to the trial after ruling out type 2 diabetes (Figure 1). Figure 2 and Appendix 4 show the rate of recruitment and randomisation across all and individual participating sites respectively. The commonest reason for women declining consent was a lack of interest in research (48%, 271/568) (Figure 1). Outcomes were assessed in 75% (148/198) of women at 28 weeks, and 90% (178/198) of women were followed-up at delivery. The rates of adherence to the intervention in the *myo*-inositol (mean 53%, SD 45) and placebo (mean 50%, SD 43) groups were similar at 28 weeks. A sensitivity analysis showed that 75% (12/16) of women with gestational diabetes in previous pregnancies took more than 75% of *myo*-inositol sachets compared to 41% of women without a history of this condition (60/145). Six of the eight App users also took more than 75% of *myo*-inositol sachets.

SECONDARY OUTCOMES

For peer review only - http://bmjopen.bmj.com/site/about/guidelines.xhtml

Glycaemic outcomes

We found significant reductions in HOMA-IR (MD -0.6; 95% CI -1.2 to 0.0) and levels of insulin (MD -2.69; 95% CI -5.26 to -0.18) in the intervention group vs control. There were no differences between the groups in other glycaemic estimates (Table 2). Sensitivity analyses of women with over 50% and over 75% adherence to the intervention showed larger reductions in HOMA-IR and insulin levels (Appendix 5).

Clinical outcomes

The proportion of women diagnosed with gestational diabetes were 14.1% (14/99) and 13.1% (13/99) in the intervention and control arms respectively at 28 weeks by modified IADPSG criteria. The overall diagnosis of gestational diabetes by delivery were 24.2% (24/99) and 20.2% (20/99) using any criteria (Table 3). The rates of preterm birth in the *myo*-inositol and placebo groups were 6% (6/99) and 10.2% (10/99) respectively, and large-for-gestational age (LGA) were 6.1% (6/99) in intervention and 11.2% (11/99) in control arm. There were no reports of any maternal anaphylactic reaction or maternal death (Table 3).

Table 3 Maternal and perinatal clinical outcomes in the EMmY study

**Outcomes**	**Intervention (*myo*-inositol) N = 99 n (%)**	**Control (placebo) N = 99 n (%)**
**Maternal**		
GDM (IADPSG) by 28 weeks	14 (14.1%)	13 (13.1%)
GDM (NICE) by 28 weeks	14 (14.1%)	9 (9.1%)
GDM (all definitions) by delivery	24 (24.2%)	20 (20.2%)
Pre-eclampsia	3 (3%)	9 (9.1%)
Hyperemesis	3 (3%)	3 (3%)
3rd/ 4th degree tear	2 (2%)	1 (1%)
Postpartum haemorrhage	17 (17.2%)	15 (15.2%)
GA at delivery (weeks; mean SD) (88;89)*	38.9 (3.2)	38.8 (2.8)
Preterm delivery (<37 weeks)	6 (6%)	10 (10.2%)
Spontaneous vaginal delivery	44 (44%)	45 (45.9%)
**Perinatal**		
Birth weight (g; mean SD) (87;88)*	3260 (533.3)	3251 (598)
Apgar score at 10 min (69;70)*	10 (0.5)	10 (0.8)
Respiratory distress syndrome	1 (1%)	1 (1%)

For peer review only - http://bmjopen.bmj.com/site/about/guidelines.xhtml

Large for gestational age	6 (6.1%)	11 (11.2%)
Small for gestational age	18 (18.4 %)	22 (22.4 %)
Admission to neonatal unit	5 (5.1%)	3 (3%)

**Number of women in the intervention and control group respectively; GDM- Gestational Diabetes Mellitus, NICE- National Institute for Health and Care Excellence, IADPSG- International Association of Diabetes and Pregnancy Study Groups, GA- Gestational Age,*

Adverse outcomes

Vomiting was reported by 15.1% (15/99) of women in the intervention arm and by 9.1% (9/99) in the control arm. Other gastrointestinal symptoms were similar between groups: diarrhoea was reported by 5.1% (5/99) and 4% (4/99) in the intervention and placebo arms respectively, and constipation by 5.1% (10/198) in both groups.

Cost outcomes

Baseline EQ-5D-5L questionnaires were completed by all women randomised, and end of trial EQ-5D-5L questionnaires by 57% (56/99) in the intervention arm and 63% (62/99) in the control group. The total cost of trial (including the supplements, diagnostic tests, clinic visits and cost of birth/delivery) was very similar in both groups (£344,127 versus £344,814 in the intervention and control group respectively) and on average around £3500 per woman. The QALYs were similar in both groups with 0.53 QALYs over 28 weeks in the control and 0.51 QALYs in the intervention group. With no major difference in costs and QALYs between groups, an incremental cost-effectiveness ratio (ICER) was not calculated and further sensitivity analysis was not explored. The costs of resources used by women in both groups as well as QALYs are reported in Appendix 6.

QUALITATIVE FINDINGS

We observed the recruitment of 28 participants across three sites. Nine women who did not want to participate in the study provided input on reasons for declining consent. Fifteen women

For peer review only

For peer review only - http://bmjopen.bmj.com/site/about/guidelines.xhtml

consented to a qualitative research interview. Fourteen healthcare professionals were interviewed, including research midwives with responsibility for recruitment at each participating site, the academic trial coordinator, the research coordinator at participating sites and a clinical academic.

Recruitment and participation

Women responded positively in the interviews about their experience of the recruitment process. They felt they received adequate information about the study to make a decision about participating, and that their questions and concerns were addressed. From our observations and interviews with women and research midwives, perceived risk of developing gestational diabetes was identified as a key factor influencing the decision about whether or not to participate in the trial. Women were more motivated to join the study if they believed they were at risk of developing gestational diabetes in their pregnancy, either due to a previous experience with the condition in an earlier pregnancy, or due to their awareness of gestational diabetes risk factors such as family history of diabetes or weight gain in pregnancy. Women without a previous history of gestational diabetes, or a family history of diabetes and those who felt they already engaged in healthy lifestyle practices did not typically view themselves to be at risk of developing gestational diabetes and were more likely to decline consent (even if the study had identified them as at-risk due to their ethnic background).

> *"… in my first pregnancy I suffered a lot. I wasn't able to have anything…even if I had an apple…I used to climb the stairs up and down,…to…bring the sugar low…it's been a nightmare for me…I don't want that to happen again…I'm just following it religiously, just to make sure…"* (Farihah, South Asian with previous gestational diabetes)

> *"I've never had a patient with previous diabetes who declined the study."* (Midwife A, Site 1)

For peer review only - http://bmjopen.bmj.com/site/about/guidelines.xhtml

For peer review only

The "natural" make-up of *myo*-inositol as a vegetarian food supplement, rather than a drug, appeared to encourage trial participation. Many women felt reassured that it was safe to take the supplement in pregnancy and research midwives said it was ideal for the predominantly Muslim target population of the study, who only eat Halal foods.

> *"... It was something natural…a natural ingredient, nothing too chemical or new or anything. So it didn't worry me".* (Amber, White European with higher education level).

Nevertheless, some women cited *"fear of supplements"* on the decliners' questionnaire as their reason for declining consent and some were concerned that participating in research involved testing the effect of new drugs, which could potentially harm the baby. Partners with these concerns sometimes discouraged participation even when the woman had indicated interest in being part of the research. High staff turnover rates in most participating sites affected study conduct. The midwives, research coordinator and trial coordinator suggested an increase in staff capacity or provision of support staff during more intense periods of study recruitment and follow-up. Research midwives highlighted that recruitment success was dependent on dissemination of trial information and engagement with the wider antenatal team.

Adherence and retention

Several of the women interviewed experienced prolonged nausea and vomiting in pregnancy and discontinued the supplements when they felt these symptoms were made worse by drinking the supplement (several mouthfuls of *"sweet"*-tasting, *"sandy"*-textured liquid). Research midwives also cited pregnancy sickness as a major reason for participant non-adherence and withdrawal. A tablet form of the supplement was suggested by women and midwives as a preferable option in future studies. The twice-daily intake of the supplement and the time restrictions on taking it one hour before or after a meal were also major barriers to long-term adherence, as women struggled to embed the regimen into their busy daily routines.

For peer review only - http://bmjopen.bmj.com/site/about/guidelines.xhtml

"…if it was in tablet form it would be easier for them to just swallow it, just like the Pregnacare... But because it's in liquid they have to take a mouthful and mouthful and mouthful... And the main reason for the withdrawal again is the fact that they were having very bad morning sickness". (Midwife G, Site 4)

Notably, adherence was reduced for some women after a normal or abnormal OGTT at the 28 weeks' visit. Those with a normal result felt the supplements were no longer needed and those with an abnormal result felt the strict regimen they had been following had not been effective. This was less of an issue for women who felt most at risk of developing gestational diabetes.

We were not able to fully evaluate the use of the App, due to difficulties in the NHS Wi-Fi connectivity, which in many cases prevented the download of the app at the point of randomisation. The few women who downloaded the App felt the automated reminders were helpful with the regimen and critical to their adherence, while other participants without the app felt they would have adhered to the sachets more consistently if they had been supported with App reminders. They also felt they would have been more consistent in recording their adherence if they could have done so on their personal mobile phones via an App, rather than in a diary, which was likened to administrative work. By contrast, some women who were not as comfortable with using technology felt the diary was easier and more straightforward.

"… I think an App or something would have been better… something that reminded me to take it… I just saw the booklet, it's just like an admin thing. That didn't help me. So an App might have done because I've got all the pregnancy Apps on my phone to remind me to take other things … (Amber, in full time employment)

DISCUSSION

Summary of key findings

For peer review only - http://bmjopen.bmj.com/site/about/guidelines.xhtml

Our multicentre pilot trial showed that it is feasible to recruit, randomise, adequately follow-up, and collect the relevant outcomes in a multi-ethnic inner-city pregnant population comprising of high-risk women, to evaluate the effects of *myo*-inositol on gestational diabetes. The trial procedures are acceptable to women and healthcare professionals. The powder form of the intervention was a barrier to adherence and women requested alternative forms such as a tablet. We observed a positive signal for efficacy in the *myo*-inositol arm than placebo for insulin resistance.

Strengths and limitations

Our pilot randomised trial was based on a clear prospective protocol,[8] and we reported the findings using recommended guidelines.[7] We showed that it is feasible to recruit the required numbers of participants within planned time frames, and to cost, across multiple maternity units. More than half of our participants were from black and ethnic minority backgrounds, who are at most risk of developing gestational diabetes. We identified the barriers and facilitators to recruitment as reported by participating mothers and healthcare professionals. The trial procedures were robustly carried out, with good accuracy in data collection (Appendix 7). The outcomes were ascertained in over 80% of women in the control and intervention arms. The qualitative study identified the main factors to sub-optimal adherence, which will need to be addressed in a full-scale trial. We showed that other outcomes such as clinical and cost-related outcomes can be collected to inform the full-scale trial.

Some of the limitations to the study included varying levels of recruitment rates across sites. Something to consider when planning the full-scale trial will be to increase staffing, to ensure that sites are fully supported during intense periods of participant recruitment and follow-up. There were more women of black and ethnic minority backgrounds in the intervention than control arms, who are more likely to have dysglycaemia in pregnancy. In a future full-scale trial,

For peer review only

For peer review only - http://bmjopen.bmj.com/site/about/guidelines.xhtml

416 we plan to minimise by ethnicity to ensure both groups are fully balanced. Some of the
417 qualitative insights suggested that adherence may have been improved if the App reminders had
418 been made available for more women. Another limitation was not being able to assess the cord
419 blood levels of the glycaemic markers in a larger proportion of participants.

420

421 *Interpretation of findings and comparison with existing literature*

422 Our consent rates were consistent with other pregnancy trials involving multi-ethnic groups,
423 citing similar reasons why eligible participants declined consent.[13, 14] Previous trials on *myo*-
424 inositol supplementation in pregnancy reported consent rates over 70% but had a predominantly
425 Caucasian population.[15, 16] Other pregnancy trials reported similar attrition rates to the EMmY
426 study, [15, 16] and made reference to phone-based reminders and diaries as crucial to adherence.[17, 18]
427 The EMmY study was not powered to detect any statistical differences in clinical or laboratory
428 outcomes, including for gestational diabetes. Hence, we refrained from reporting comparative
429 estimates for dichotomous outcomes. We identified the expected rates of gestational diabetes in
430 the control arm that will inform the sample size calculations for the full-scale trial. We also
431 observed that women continued to be diagnosed with gestational diabetes beyond 28 weeks,
432 therefore a definitive trial would need to assess onset of gestational diabetes at any time in
433 pregnancy along with critically important clinical outcomes as main outcomes.

434

435 *Clinical and research implications*

436 Overall, the pilot study achieved 'green' status in rates of recruitment, attrition, and data
437 collection, and 'amber' in adherence to the intervention at 28 weeks, as markers of progression
438 to full-scale trial. Our qualitative study has identified the main reason for reduced adherence to
439 be dissatisfaction with the powder form of the intervention. The findings were further discussed
440 with the independent Steering Committee and Katie's Team, the patient and public involvement

For peer review only - http://bmjopen.bmj.com/site/about/guidelines.xhtml

support arm of the study, who recommended use of *myo*-inositol tablets instead of powder when progressing to a full-scale trial.

We explored the differences in the continuous laboratory outcomes for signals of efficacy, as these would inform the choice of main outcomes for the full-scale trial. Despite lower than anticipated overall adherence to the intervention, the higher levels of urinary inositol in the intervention group makes the comparison of outcomes valid. In randomised trials on *myo*-inositol in pregnancy to prevent or treat gestational diabetes, *myo*-inositol showed a significant reduction in levels of fasting and postprandial blood glucose, HOMA-IR and insulinaemia.[19-21] We found a reduction in insulin and HOMA-IR in the intervention arm vs control, indicating a potentially increased insulin sensitivity with *myo*-inositol. Insulin levels and HOMA-IR appeared to reduce further with improved adherence. Our findings are in line with previous observations that *myo*-inositol reduces blood glucose levels by acting as an insulin sensitising agent. [16, 21] We did not observe any differences in the fasting or 2-hour blood glucose levels, but improved adherence showed the potential to reduce 2-hour glucose levels in the intervention group. It is likely that with improved adherence, any potential differences between groups will become clearer in any future studies.

Although there were no differences in the levels of c-peptide between the two arms, women who adhered to the intervention appeared to have a reduction in c-peptide levels, indicative of a reduction in insulin resistance. Participants in our study continued taking *myo*-inositol even after the diagnosis of gestational diabetes. We observed that fewer women in the intervention arm had large for gestational age (LGA) babies than in the control arm. The small sample size of this pilot trial means no statistical significance can be attributed to these estimates.[22] A future large scale trial will be needed to evaluate if the improvement in insulin sensitivity with *myo*-inositol contributed to the observed reduction in LGA babies.

For peer review only - http://bmjopen.bmj.com/site/about/guidelines.xhtml

467 *Conclusions*

468 The EMmY pilot trial is the first UK based multi-centre trial involving ethnically diverse high-
469 risk women from inner city NHS hospitals in a trial of *myo*-inositol vs placebo. We have
470 reported on acceptability, cost and quality outcomes, as well as the potential effect of *myo*-
471 inositol on gestational diabetes. There is an indication of efficacy of *myo*-inositol in lowering
472 insulin resistance, with the potential for preventing gestational diabetes. In order to tackle the
473 observed barriers to adherence, we plan to use a tablet form of *myo*-inositol in a large-scale
474 definitive trial, with an internal pilot first demonstrating the intended levels of adherence.
475 We now need a large-scale definitive trial adequately powered to ascertain the effect of *myo*-
476 inositol in a tablet form on gestational diabetes and adverse maternal and perinatal outcomes.
477

478 **Authors' contributions**

479 CEA- wrote the first and final version of the manuscript, collected and analysed the qualitative
480 data.
481 ZD, DL, JDO, JH- coordinated the study, contributed to the methodology and the final version
482 of the manuscript.
483 LS and AH- supervised the qualitative evaluation and has contributed to the final version of the
484 manuscript.
485 EP- developed and conducted the economic evaluation. EP also contributed to the final version
486 of the manuscript.
487 AT, JDa, SS, LP, AK, JM, GH, KSK and MSH- provided clinical input and contributed to the
488 final version of the manuscript.
489 JZ, MDCP, TP- developed and lead the statistical evaluation and contributed to the final version
490 of the manuscript.
491 FJGC- conducted the statistical analysis and contributed to the final version of the manuscript.

For peer review only - http://bmjopen.bmj.com/site/about/guidelines.xhtml

ST- designed and developed the trial, reviewed the manuscript and has contributed to the final version of the manuscript.

Acknowledgements

The authors acknowledge Pharmasure for the donation of *myo*-inositol and placebo tablets to the EMmY study and Montuno software for the discounted Dosecast app. The authors are grateful to the patient advisers and site midwives for their contributions towards the study.

The EMmY pilot trial was supported by the National Institute for Health Research (NIHR) Programme Grants for Applied Research. The views expressed are those of the authors and not necessarily those of the NHS, the NIHR or the Department of Health and Social Care.

KSK is a Distinguished Investigator funded by the Beatriz Galindo (Senior Modality) Program grant given to the University of Granada by the Ministry of Science, Innovation, and Universities of the Spanish Government.

Ethical approval and funding statement

The EMmY trial is sponsored by Queen Mary University of London and funded by Barts Charity, grant number MGU0373. Ethical approval was obtained from the London Queen Square Research Ethics Committee (17/LO/1741). The sponsor and the funder have no role in the study conduct, analysis and interpretation of the findings, and dissemination of the results. EP and AH are also supported by the NIHR Collaboration for Leadership in Applied Health Research at Barts Health NHS Foundation Trust (NIHR ARC North Thames). Professor Khalid S. Khan is Distinguished Investigator funded by the Beatriz Galindo (senior modality) grant to the University of Granada by the Spanish Ministry of Education.

Data Sharing Statement

For peer review only - http://bmjopen.bmj.com/site/about/guidelines.xhtml

Release of data will be subject to a data use agreement between the contractor and the third party requesting the data. The data use agreement must detail the agreed use and appropriate management of the research data to be shared and include a requirement for recognition of the contribution of the researchers who generated the data and the original funder.

Competing interests

None

For peer review only - http://bmjopen.bmj.com/site/about/guidelines.xhtml

References

1. Metzger BE, Lowe LP, Dyer AR, et al. Hyperglycemia and adverse pregnancy outcomes. *N Engl J Med*. May 2008;358(19):1991-2002. doi:10.1056/NEJMoa0707943
2. Bellamy L, Casas JP, Hingorani AD, Williams D. Type 2 diabetes mellitus after gestational diabetes: a systematic review and meta-analysis. *Lancet*. May 2009;373(9677):1773-9. doi:10.1016/S0140-6736(09)60731-5
3. Boney CM, Verma A, Tucker R, Vohr BR. Metabolic syndrome in childhood: association with birth weight, maternal obesity, and gestational diabetes mellitus. *Pediatrics*. Mar 2005;115(3):e290-6. doi:10.1542/peds.2004-1808
4. Rogozińska E, Chamillard M, Hitman GA, Khan KS, Thangaratinam S. Nutritional manipulation for the primary prevention of gestational diabetes mellitus: a meta-analysis of randomised studies. *PLoS One*. 2015;10(2):e0115526. doi:10.1371/journal.pone.0115526
5. Vitagliano A, Saccone G, Cosmi E, et al. Inositol for the prevention of gestational diabetes: a systematic review and meta-analysis of randomized controlled trials. *Arch Gynecol Obstet*. 01 2019;299(1):55-68. doi:10.1007/s00404-018-5005-0
6. Crawford TJ, Crowther CA, Alsweiler J, Brown J. Antenatal dietary supplementation with myo-inositol in women during pregnancy for preventing gestational diabetes. *Cochrane Database Syst Rev*. Dec 2015;(12):CD011507. doi:10.1002/14651858.CD011507.pub2
7. Eldridge SM, Chan CL, Campbell MJ, et al. CONSORT 2010 statement: extension to randomised pilot and feasibility trials. *Pilot Feasibility Stud*. 2016;2:64. doi:10.1186/s40814-016-0105-8
8. Amaefule CE, Drymoussi, Z., Dodds, J., Sweeney, L., Pizzo, E., Daru, J., Robson, J., Poston, L., Khalil, A., Myers, J. and Harden, A.,. Effectiveness and acceptability of myo-inositol nutritional supplement in the prevention of gestational diabetes (EMmY): a protocol for a randomised, placebo-controlled, double-blind pilot trial. *BMJ open*. 2018;8(9):p.e022831.
9. National Institute for Health and Care Excellence. Diabetes in pregnancy: management from pre-conception to the post-natal period. Accessed 26th November, 2019. https://www.nice.org.uk/guidance/ng3/chapter/1-Recommendations#gestational-diabetes-2
10. Braun, V. and Clarke, V. Using thematic analysis in psychology. Qualitative research in psychology. *Qualitative Research in Psychology*. 2006;*3* (2):pp.77-101.
11. Amaefule CE, Sweeney, L., Harden, A., Thangaratinam, S.,. Pilot trial on myo-inositol supplementation in pregnancy: A qualitative study with women and research staff on the feasibility and acceptability of the trial and intervention. *To be published at BMJ open*. 2020;
12. Moss N, Daru J, Lanz D, Thangaratinam S, Khan KS. Involving pregnant women, mothers and members of the public to improve the quality of women's health research. *BJOG*. Feb 2017;124(3):362-365. doi:10.1111/1471-0528.14419
13. Husain S, Allotey J, Drymoussi Z, et al. Effects of oral probiotic supplements on vaginal microbiota during pregnancy: a randomised, double-blind, placebo-controlled trial with microbiome analysis. *BJOG*. 01 2020;127(2):275-284. doi:10.1111/1471-0528.15675
14. Poston L, Bell R, Croker H, et al. Effect of a behavioural intervention in obese pregnant women (the UPBEAT study): a multicentre, randomised controlled trial. *Lancet Diabetes Endocrinol*. Oct 2015;3(10):767-77. doi:10.1016/S2213-8587(15)00227-2
15. D'Anna R, Scilipoti A, Giordano D, et al. myo-Inositol supplementation and onset of gestational diabetes mellitus in pregnant women with a family history of type 2 diabetes: a prospective, randomized, placebo-controlled study. *Diabetes Care*. Apr 2013;36(4):854-7. doi:10.2337/dc12-1371
16. D'Anna R, Di Benedetto A, Scilipoti A, et al. Myo-inositol Supplementation for Prevention of Gestational Diabetes in Obese Pregnant Women: A Randomized Controlled Trial. *Obstet Gynecol*. Aug 2015;126(2):310-5. doi:10.1097/AOG.0000000000000958
17. Laughland AaK, L.,. Should participants be left to their own devices? Comparing paper and smartphone diaries in psychological research. *Journal of applied research in memory and cognition*. 2018;7(4):pp.552-563.

For peer review only

For peer review only - http://bmjopen.bmj.com/site/about/guidelines.xhtml

18. Brennan M, Clarke M, Devane D, Dowling M. A qualitative study of the factors influencing recruitment to a pilot trial on the prevention of striae gravidarum. *BMC Pregnancy Childbirth*. Feb 2020;20(1):103. doi:10.1186/s12884-020-2781-x

19. Fraticelli F, Celentano C, Zecca IA, et al. Effect of inositol stereoisomers at different dosages in gestational diabetes: an open-label, parallel, randomized controlled trial. *Acta Diabetol*. Aug 2018;55(8):805-812. doi:10.1007/s00592-018-1157-4

20. Corrado F, D'Anna R, Di Vieste G, et al. The effect of myoinositol supplementation on insulin resistance in patients with gestational diabetes. *Diabet Med*. Aug 2011;28(8):972-5. doi:10.1111/j.1464-5491.2011.03284.x

21. Miñambres I, Cuixart G, Gonçalves A, Corcoy R. Effects of inositol on glucose homeostasis: Systematic review and meta-analysis of randomized controlled trials. *Clin Nutr*. 06 2019;38(3):1146-1152. doi:10.1016/j.clnu.2018.06.957

22. Arnold DM, Burns KE, Adhikari NK, et al. The design and interpretation of pilot trials in clinical research in critical care. *Crit Care Med*. Jan 2009;37(1 Suppl):S69-74. doi:10.1097/CCM.0b013e3181920e33

Figure Legends, Tables and Appendices

Figure 1 CONSORT diagram for the EMmY study

Figure 2 Rate of recruitment and randomisation in all participating sites in the EMmY trial

Table 1 Baseline characteristics of EMmY participants randomised to *myo*-inositol vs placebo

Table 2 Glycaemic outcomes in the intervention and control arms of the EMmY trial

Table 3 Maternal and perinatal clinical outcomes in the EMmY study

Appendix 1 Clinical maternal and perinatal outcomes assessed within the EMmY study

Appendix 2 Criteria for progression to a full-scale trial

Appendix 3 Characteristics of the 15 women interviewed using pseudonyms

Appendix 4 Proportion of screened, recruited and randomised women in each participating site

Appendix 5 Costs and QALYS in both intervention and control groups

Appendix 6 Impact of adherence at 28 weeks on key clinical and laboratory outcomes

Appendix 7 Completeness of clinical outcome data

For peer review only - http://bmjopen.bmj.com/site/about/guidelines.xhtml

For peer review only

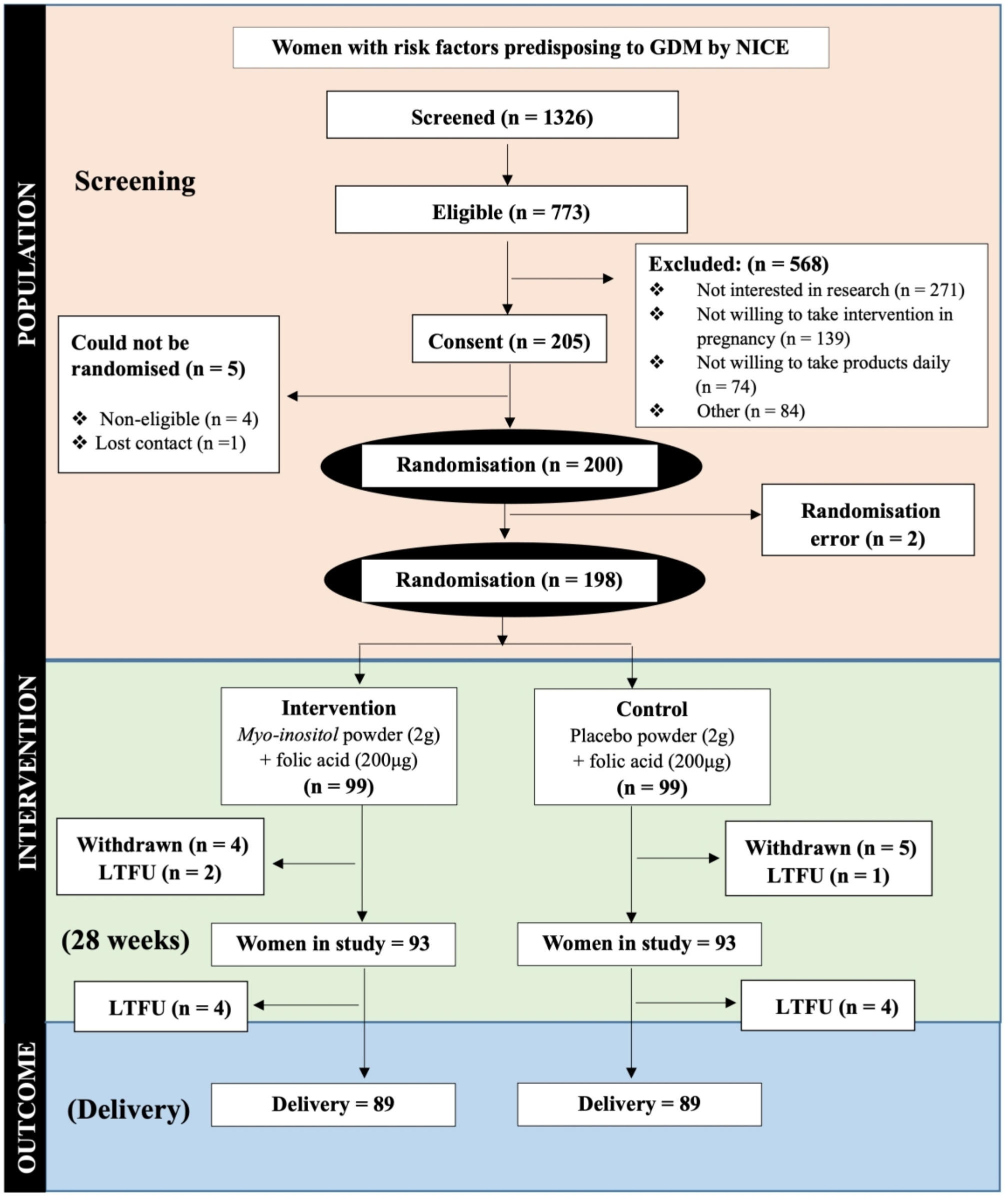

Figure 1. Flow chart of participants in the EMmY study

196x233mm (330 x 330 DPI)

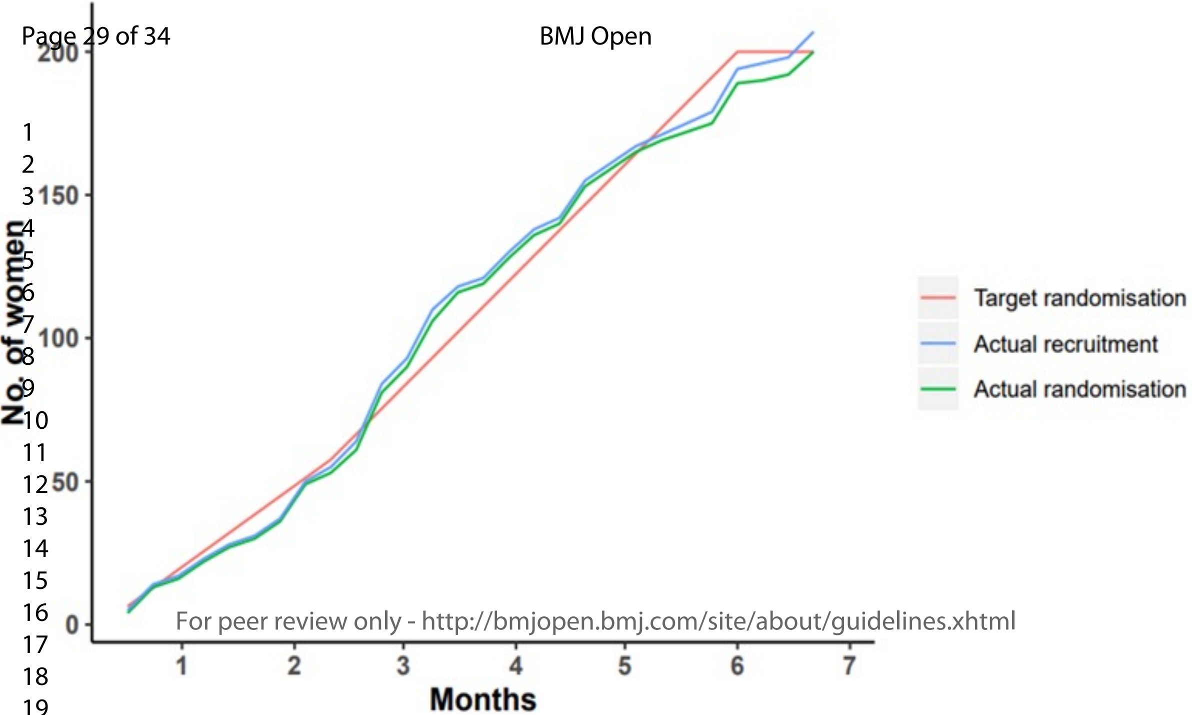

For peer review only - http://bmjopen.bmj.com/site/about/guidelines.xhtml

CONSORT 2010 checklist of information to include when reporting a pilot or feasibility trial*

Section/Topic	Item No	Checklist item	Reported on page No
**Title and abstract**			
	1a	Identification as a pilot or feasibility randomised trial in the title	1
	1b	Structured summary of pilot trial design, methods, results, and conclusions (for specific guidance see CONSORT abstract extension for pilot trials)	3
**Introduction**			
Background and objectives	2a	Scientific background and explanation of rationale for future definitive trial, and reasons for randomised pilot trial	5
	2b	Specific objectives or research questions for pilot trial	5
**Methods**			
Trial design	3a	Description of pilot trial design (such as parallel, factorial) including allocation ratio	6
	3b	Important changes to methods after pilot trial commencement (such as eligibility criteria), with reasons	n/a
Participants	4a	Eligibility criteria for participants	6
	4b	Settings and locations where the data were collected	6
	4c	How participants were identified and consented	7-8
Interventions	5	The interventions for each group with sufficient details to allow replication, including how and when they were actually administered	6
Outcomes	6a	Completely defined prespecified assessments or measurements to address each pilot trial objective specified in 2b, including how and when they were assessed	6-7
	6b	Any changes to pilot trial assessments or measurements after the pilot trial commenced, with reasons	n/a
	6c	If applicable, prespecified criteria used to judge whether, or how, to proceed with future definitive trial	8
Sample size	7a	Rationale for numbers in the pilot trial	7
	7b	When applicable, explanation of any interim analyses and stopping guidelines	n/a
Randomisation:			
Sequence generation	8a	Method used to generate the random allocation sequence	7
	8b	Type of randomisation(s); details of any restriction (such as blocking and block size)	7-8
Allocation concealment mechanism	9	Mechanism used to implement the random allocation sequence (such as sequentially numbered containers), describing any steps taken to conceal the sequence until interventions were assigned	7-8

For peer review only

Implementation	10	Who generated the random allocation sequence, who enrolled participants, and who assigned participants to interventions	7-8
Blinding	11a	If done, who was blinded after assignment to interventions (for example, participants, care providers, those assessing outcomes) and how	8
	11b	If relevant, description of the similarity of interventions	6
Statistical methods	12	Methods used to address each pilot trial objective whether qualitative or quantitative	8-9
**Results**			
Participant flow (a diagram is strongly recommended)	13a	For each group, the numbers of participants who were approached and/or assessed for eligibility, randomly assigned, received intended treatment, and were assessed for each objective	10
	13b	For each group, losses and exclusions after randomisation, together with reasons	10
Recruitment	14a	Dates defining the periods of recruitment and follow-up	10
	14b	Why the pilot trial ended or was stopped	n/a
Baseline data	15	A table showing baseline demographic and clinical characteristics for each group	10
Numbers analysed	16	For each objective, number of participants (denominator) included in each analysis. If relevant, these numbers should be by randomised group	10-11
Outcomes and estimation	17	For each objective, results including expressions of uncertainty (such as 95% confidence interval) for any estimates. If relevant, these results should be by randomised group	10-11
Ancillary analyses	18	Results of any other analyses performed that could be used to inform the future definitive trial	12-15
Harms	19	All important harms or unintended effects in each group (for specific guidance see CONSORT for harms)	11
	19a	If relevant, other important unintended consequences	n/a
**Discussion**			
Limitations	20	Pilot trial limitations, addressing sources of potential bias and remaining uncertainty about feasibility	15-16
Generalisability	21	Generalisability (applicability) of pilot trial methods and findings to future definitive trial and other studies	17
Interpretation	22	Interpretation consistent with pilot trial objectives and findings, balancing potential benefits and harms, and considering other relevant evidence	16-17
	22a	Implications for progression from pilot to future definitive trial, including any proposed amendments	17-18
**Other information**			
Registration	23	Registration number for pilot trial and name of trial registry	3
Protocol	24	Where the pilot trial protocol can be accessed, if available	n/a
Funding	25	Sources of funding and other support (such as supply of drugs), role of funders	21
	26	Ethical approval or approval by research review committee, confirmed with reference number	21

For peer review only

Citation: Eldridge SM, Chan CL, Campbell MJ, Bond CM, Hopewell S, Thabane L, et al. CONSORT 2010 statement: extension to randomised pilot and feasibility trials. BMJ. 2016;355.

*We strongly recommend reading this statement in conjunction with the CONSORT 2010, extension to randomised pilot and feasibility trials, Explanation and Elaboration for important clarifications on all the items. If relevant, we also recommend reading CONSORT extensions for cluster randomised trials, non-inferiority and equivalence trials, non-pharmacological treatments, herbal interventions, and pragmatic trials. Additional extensions are forthcoming: for those and for up to date references relevant to this checklist, see www.consort-statement.org.

For peer review only

Appendix 1 Clinical maternal and perinatal outcomes assessed in the EMmY study.

**Clinical outcomes**	
**Maternal**	**Fetal and neonatal**
Gestational diabetes	Hypocalcaemia
Pre-eclampsia	Hypoglycaemia
Postpartum haemorrhage	Birth weight
Gestational age at delivery	Macrosomia (birth weight >4.5Kg),
Preterm delivery (< 34 and < 37 weeks)	small for gestational age (<10th centile)
Mode of delivery	Large for gestational age (>90th Centile)
Perineal trauma	Respiratory distress syndrome
Admission to the ITU	Shoulder dystocia
Admission to HDU	Apgar score at 10 minutes
Maternal infection	Birth trauma
Maternal death	Hyperbilirubinaemia
	Septicaemia
	Admission to NICU
	Still birth
	Neonatal death.

NB: Gestational diabetes is diagnosed based on the National Institute for Care and Excellence (NICE) and International Association of Diabetes and Pregnancy Study Groups (IADPSG) criteria.
ITU - intensive care unit, HDU - High dependency unit, NICU - neonatal intensive care unit.

Appendix 2 Criteria for progression to a full-scale trial

**Feasibility objectives and related data to be collected**	**GREEN zone**	**AMBER zone**	**RED zone**
i) Consent rates of eligible women	At least 25% of eligible women agreeing to participate.	Rate between 11 and 24% women agreeing to participate	Rate <10% of eligible women agreeing to participate
ii) Attrition rate after randomisation by 28 weeks, and by delivery (withdrawal or loss to follow up)	No more than 20% of randomised women lost or withdrawn	21-50% of randomised women lost or withdrawn	More than 50% of randomised women lost or withdrawn
iii) Rate of women who take more than 75% of sachets by 28 weeks and by delivery (of those who remain on the study)	Adherence to allocated treatment in >80% of women remaining on the study.	Adherence to allocated treatment in between 51-79% of women remaining on the study.	Adherence to allocated treatment in <50% of women
iv) Collection of data on clinical outcomes.	Complete data available of >80% of study sample.	Missing data between 21-50% of study sample.	Data missing of >50% of study sample.

For peer review

Appendix 3 Characteristics of the 15 women interviewed

Ethnicity	Educational level	History of GDM (Y/N)	Adherence level (%)
Middle Eastern	Higher	N	50-75
South Asian	Higher	Y	>75
Middle Eastern	Higher	N	>75
White European	Higher	Y	>75
White European	Secondary	N	<50
South Asian	Higher	N	>75
South Asian	Higher	Y	>75
Black African/ Caribbean	Higher	N	<50
South Asian	Higher	N	>75
South Asian	Secondary	N	<50
White European	Higher	N	<50
White European	Secondary	N	>75
White European	Higher	N	>75
White European	Higher	N	<50
Middle Eastern	Higher	Y	>75

Appendix 4 Proportion of screened, recruited and randomised women in each participating site

Sites	Women screened (N)	Eligible of those screened n(%)	Recruited of those eligible n(%)	Randomised of those recruited n(%)
MRI	85	78 (91.8%)	46 (59%)	46 (100%)
NUH	144	115 (79.9%)	22 (19.1%)	22 (100%)
RLH	320	192 (60%)	53 (27.6%)	49 (92.5%)
STH	177	145 (81.9%)	41 (28.3%)	38 (92.7%)
WXH	600	243 (40.5%)	43 (17.7%)	43 (100%)
Overall	1326	773 (58.3%)	205	198

NB: MRI- Manchester Royal Infirmary (St Mary's Hospital) in Manchester, NUH- Newham Hospital, London, RLH- Royal London Hospital, London, STH- St George's Hospital, London, WXH- Whipps Cross Hospital, London.

For peer review only

Appendix 5 Impact of adherence at 28 weeks on glycaemia related outcomes

**Glycaemia related outcomes (unit) (intervention; control)***			
**≥ 50%**			
OGTT fasting (mmol/l) (40;40)	4.57 (0.6)	4.57 (0.5)	0.00 (0.2;0.2)
OGTT 2 hours (mmol/l) (39;40)	6.15 (2.2)	6.27 (1.5)	-0.11 (-0.9;0.7)
C-peptide (µg/L) (53;63)	1608.65 (723.2)	1907.71 (868.4)	-288.79 (-644.1;59.7)
Leptin (µg/L) (53;63)	42.81 (23.4)	39.31 (15.0)	4.09 (-4.8;12.7)
Insulin (mlU/L) (37;35)	11.23 (6.74)	14.81 (9.67)	**-3.34 (-6.61; -0.12)**
Adiponectin (mg/L) (53;63)	6.5 (3.6)	6.16 (3.6)	0.21 (-1.3;2.0)
Urinary inositol (mg/L) (53;65)	261 (164.6)	191.02 (117.7)	69.52 (3.2;136.8)
HOMA-IR (50;62)	2.25 (1.4)	3.06 (2.3)	**-0.8 (-1.6;-0.0)**
**≥ 75%**			
OGTT fasting (mmol/l) (34;32)	4.58 (0.6)	4.58 (0.5)	0.00 (0.3;0.3)
OGTT 2 hours (mmol/l) (33;32)	6.12 (2.35)	6.22 (1.53)	-0.1 (-1;0.9)
C-peptide (µg/L) (31;28)	1517.42 (573.9)	1937.86 (932.9)	**-425.4 (-808.8; -45.6)**
Leptin (µg/L) (31;28)	41.92 (24.4)	37.51 (11.9)	4.8 (-5.6;14.4)
Insulin (mlU/L) (31;28)	10.75 (5.78)	15.15 (10.4)	-4.57 (-8.2;0.99)
Adiponectin (mg/L) (31;28)	6.57 (3.6)	5.7 (3.4)	0.87 (-0.9;2.7)
Urinary inositol (mg/L) (32;27)	249.41 (152.7)	189.17 (110.3)	57.2 (-8.7;129.1)
HOMA-IR (28;27)	2.12 (1.1)	3.12 (2.5)	**-1.07 (-2; -0.2)**

NB: *GDM- Gestational Diabetes Mellitus, NICE- National Institute for Health and Care Excellence, IADPSG- International Association of Diabetes and Pregnancy Study Groups, GA- Gestational Age*
**Number of women in the intervention and control group respectively.*

Appendix 6 Costs and QALYS in both intervention and control groups

**Resource use costs**	**Intervention (*myo*-inositol) (£)**	**Control (placebo) (£)**
Supplements	2527	492
Laboratory tests	518	574
- Average costs (n)	5.51 (94)	6.17 (93)
Clinic visits	31,815	39,995
- Average costs per woman (n. of visits)	321.4 (390)	404 (389)
Adverse events	n/a	n/a
Delivery	309,268	303,753
- Average costs per woman (n)	3,514 (88)	3,472 (89)
**Total costs**	**312,121**	**344,814**
QALYs		
- Average QALY per woman (n)	0.51 (56)	0.53 (62)
**Total QALYs**	**28.4**	**32.6**

NB: QALYs – Quality Adjusted Life Years

For peer review only

Appendix 7 Completeness of clinical outcome data

Proportion of data missing			
**Clinical outcomes**	**Intervention (Myo-inositol) N = 99 n/N (%)**	**Control (Placebo) n = 99 n/N (%)**	**Total n = 198 n/N (%)**
GDM (NICE)- 28 weeks	14 (14.1%)	17 (17.2%)	31 (15.7%)
GDM (IADPSG)- 28 weeks	15 (15.2%)	17 (17.2%)	32 (16.2%)
GDM (all definitions)- by delivery	8 (8.1%)	7 (7.1%)	15 (7.6%)

***NB**: some GDM diagnosis were made between 28 weeks gestation and delivery, so would be missing at 28 weeks but not at delivery*

For peer review only
